# Factors associated with local herb use during pregnancy and labor among women in Kigoma region, Tanzania, 2014–2016

**DOI:** 10.1186/s12884-020-2735-3

**Published:** 2020-02-21

**Authors:** R. Fukunaga, D. Morof, C. Blanton, A. Ruiz, G. Maro, F. Serbanescu

**Affiliations:** 1 Division of Reproductive Health, National Center for Chronic Disease Prevention and Health Promotion, Centers for Disease Control and Prevention, Atlanta, Georgia USA; 2 Epidemic Intelligence Service, Atlanta, Georgia USA; 3 United States Public Health Service, Atlanta, USA; 4 Bloomberg Philanthropies, New York, New York, United States

**Keywords:** Maternal health, Local herb use, Adverse pregnancy outcomes, Prevalence, Population-based survey, Kigoma, Tanzania

## Abstract

**Background:**

Despite research suggesting an association between certain herb use during pregnancy and delivery and postnatal complications, herbs are still commonly used among pregnant women in sub-Sahara Africa (SSA). This study examines the factors and characteristics of women using local herbs during pregnancy and/or labor, and the associations between local herb use and postnatal complications in Kigoma, Tanzania.

**Methods:**

We analyzed data from the 2016 Kigoma Tanzania Reproductive Health Survey (RHS), a regionally representative, population-based survey of reproductive age women (15–49 years). We included information on each woman’s most recent pregnancy resulting in a live birth during January 2014–September 2016. We calculated weighted prevalence estimates and used multivariable logistic regression to calculate adjusted odds ratios (aOR) and 95% confidence intervals (CI) for factors associated with use of local herbs during pregnancy and/or labor, as well as factors associated with postnatal complications.

**Results:**

Of 3530 women, 10.9% (CI: 9.0–13.1) used local herbs during their last pregnancy and/or labor resulting in live birth. The most common reasons for taking local herbs included stomach pain (42.9%) and for the health of the child (25.5%). Adjusted odds of local herb use was higher for women reporting a home versus facility-based delivery (aOR: 1.6, CI: 1.1–2.2), having one versus three or more prior live births (aOR: 1.8, CI: 1.4–2.4), and having a household income in the lowest versus the highest wealth tercile (aOR: 1.4, CI: 1.1–1.9). Adjusted odds of postnatal complications were higher among women who used local herbs versus those who did not (aOR: 1.5, CI: 1.2–1.9), had four or more antenatal care visits versus fewer (aOR: 1.4, CI: 1.2–1.2), and were aged 25–34 (aOR: 1.1, CI: 1.0–1.3) and 35–49 (aOR: 1.3, CI: 1.0–1.6) versus < 25 years.

**Conclusions:**

About one in ten women in Kigoma used local herbs during their most recent pregnancy and/or labor and had a high risk of postnatal complications. Health providers may consider screening pregnant women for herb use during antenatal and delivery care as well as provide information about any known risks of complications from herb use.

## Background

The World Health Organization (WHO) estimates that traditional and complementary medicine, accounts for 80% of health-care in the world [1]. Despite its frequency, there is a lack of standards ensuring the safe use of herbs for health purposes and literature linking herb use to various forms of adverse health events [2, 3] such as higher rates of pregnancy losses, increased use of caesarean section, increased frequency of maternal complications after delivery [4–6], increased occurrence of congenital malformations [7], congestive heart failure in newborns [8], and perinatal deaths [9].

In Tanzania, between 60 and 70% of the population seeks health care through the use of traditional medicine [10, 11], and tends to rely on traditional medicinal products to treat a wide-range of health conditions [12] including management of HIV/AIDS [13, 14], malaria [15], hypertension [16], sexually transmitted infections [17], and infant and child illnesses [18]. Studies on the estimated prevalence of local herbal use during pregnancy in sub-Saharan Africa (SSA) range between 40 and 90% [19, 20], but the prevalence of local herb use in Tanzania remains largely unknown. However, it is widely understood that traditional medicine, which is commonly comprised of herbal products, is popular in Tanzania due to the accessibility, availability, and low cost of herbal products compared with modern health care services across this region [21]. Use of local herbs during pregnancy may be particularly high as traditional healers and birth attendants remain key players in the delivery of care and are known to integrate traditional medicine into their practices [22]. Healthcare providers in SSA also continue to recommend local herbal products for treating a myriad of health issues during pregnancy [23–25], despite insufficient data to inform the safe use of herbal products during pregnancy [26, 27].

Previous global health studies have identified a number of maternal characteristics associated with herb use including maternal age [28, 29], marital status [28–31], education level [32, 33], birth order [29, 34], antenatal care [35–38], socioeconomic status [28, 32, 36], and rural residence [39–41].

Most of the current literature on the use of herbs in Tanzania has focused on conducting pharmacological studies on medicinal plants found in the region [14–18]. To date, no studies in this setting have examined local herb use (LHU) among pregnant women in relation to both maternal characteristics and pregnancy outcomes. Our study examines LHU in a representative sample of women with recent births in Kigoma Region, including the patterns of LHU, socio-demographic characteristics, and factors associated with LHU during pregnancy and/or labor. In a separate analysis, we explore the association of LHU during pregnancy and/or labor and early postnatal obstetric complications.

## Methods

### Study setting and design

Kigoma Region, located in the northwest corner of Tanzania by Lake Tanganika, covers 45,066 km^2^ and had a population of 2,127,930 in 2012. Approximately 83% of the population is classified as rural with farming as the primary economic activity [42]. In 2015, emergency obstetric and neonatal care facilities provided care for 83% of all direct obstetric complications. Eight out of ten maternal deaths in facilities were due to direct obstetric causes in 2011–2015 [43].

A regionally representative multistage survey of reproductive age women (15–49 years) was conducted in July–September 2016 in Kigoma, as part of a larger evaluation effort of the *Project to Reduce Maternal Deaths in Tanzania.* The project is a collaboration between Centers for Disease Control and Prevention (CDC) and the Tanzania Ministry of Health, Community Development, Gender, the Elderly and Children (MoHCDGEC), Thamini Uhai, Vital Strategies and EngenderHealth, with financial support from Bloomberg Philanthropies and the H&B Agerup Foundation.

The survey was approved by the CDC Institutional Review Board and the Tanzania National Institute for Medical Research (NIMR) as the main evaluation approach to assess the maternal, child and reproductive health status, health service utilization and behaviors of women ages 15–49 in Kigoma region. Informed consent was obtained from household respondents at the beginning of the household interviews, and separately from eligible respondents at the start of the individual interviews. The consent was given verbally and attested on the paper questionnaire by the interviewer’s signature, date and time of giving consent, which were shown to the respondent, in accordance with the Tanzania NIMR requirements for human subject participation in population surveys. No compensation of any kind was provided to respondents who agreed to voluntarily participate in the survey. A detailed description of the survey methods and procedures are available elsewhere [44].

The survey included a regionally representative probability sample of women ages 15–49 that was selected using the 2012 National Census as the sampling frame. Maps and household listings for each enumeration area were updated during the month prior to the data collection. Trained interviewers obtained informed consent prior to conducting household and individual interviews. If obtained, interviewers then conducted confidential, face-to-face interviews using standardized questionnaires to collect information on households and individual women. The individual questionnaire asked information about a woman’s background characteristics, contraceptive behaviors and use, fertility, and detailed information about the most recent births (i.e., births during January 2014–September 2016) (see Additional file 1).

For the 2016 Kigoma Reproductive Health Survey (RHS), a total of 6461 of 6630 sampled households (97.5%) completed an interview. Within the responding households, 7023 of 7506 women aged 15–49 years (93.6%) responded. Of these women, 3531 (50.1%) women reported at least one live birth between January 2014 and September 2016 and were included in the analysis.

This study derived its main findings from the birth histories, which contained detailed information on birth outcomes (live birth or stillbirth), antenatal care, place and type of delivery and health behaviors during and after pregnancy, including LHU.

### Inclusion criteria

For our analyses, we included information on LHU during the last pregnancy and/or labor resulting in live birth for all women who had a live born infant between January 2014 and September 2016. Details on the inclusion criteria and methodology of the survey in general are included in the 2016 Kigoma Region RHS Final Report [44].

### Assessment of sociodemographic indicators

The sociodemographic indicators of interests included these selected variables: 1) *Age* (under 25, 25 to 34, 35 to 49); 2) *Marital status* (currently in a union, previously in a union, never in a union); 3) *Residence* (urban, rural); 4) *Highest education completed* (none, some primary, completed primary and/or higher); and 5) *Household wealth index based on household assets* (low, middle, high).

### Characteristics of pregnancy and delivery

The characteristics of pregnancy and delivery among women reporting LHU were captured through the following variables: 1) *LHU during their pregnancy and/or labo*r (yes/no); 2) *Reasons for taking herbs* (Induce or sustain labor, treat malaria, treat cold/flu, treat headache, treat convulsions, treat vaginal bleeding, treat stomach pain, for the health of the child, to avoid miscarriage, other (specify)); 3) *When LHU was initiated and stopped* (1st trimester, 2nd trimester, 3rd trimester, just before delivery, during/after delivery, does not remember); 4) *Birth order* (continuous); 5) *Recommended antenatal care (ANC) received* (yes/no). ANC responses were recoded as a dichotomous variable where women either met or did not meet the Tanzanian national guidance recommendation of four or more ANC visits during pregnancy [45]; 5) *Gestational age at delivery* (continuous); 6) *Place of delivery* (hospital/health center/dispensary, home, unknown); and 7) *Postnatal complications* (yes/no): Included only pregnancy obstetric complications with onset during the first 6 weeks of the postnatal period. Postnatal complications included severe bleeding; vaginal discharge, surgical infection, fainting, coma, high fever, pelvic pain, urinary incontinence, and bowel incontinence. Two obstetrician/gynecologist (Ob/Gyn) epidemiologists reviewed the survey responses to include only obstetric complications in the final analysis.

### Statistical analysis

#### Descriptive statistics

We calculated prevalence estimates with 95% confidence intervals for the following selected characteristics: overall LHU, reasons for LHU, LHU by demographic factors and clinically-relevant postnatal obstetric complications by LHU. We also examined the average number of days local herbs were used during pregnancy and/or labor and the proportion of herb users receiving recommended ANC.

#### Chi-squared statistics

We used chi-squared statistics to assess whether LHU during pregnancy and/or labor varied by residence, age, education level, marital status, wealth, parity, recommended ANC visits, and place of delivery. Also, chi-square tests were performed on whether postnatal obstetric complications varied with LHU during pregnancy and/or labor and by residence, age, education level, marital status, wealth, parity, recommended ANC visits, and place of delivery.

#### Multivariable models

We constructed two multivariable logistic regression models. The first model examined factors associated with LHU. The second model examined the association between LHU during pregnancy/labor and postnatal obstetric complications, while adjusting for any potential confounders. For both models, only the significant associations (*p* < 0.05) in the bivariate analyses were included in the full model, which were then removed sequentially based on a threshold of a *p* < 0.05. For the final multivariable model, we included age in our final model a priori because it is a well-documented risk factor for postnatal complications [46]. The results are presented as adjusted odds ratios (aOR) and 95% confidence intervals (CI). We performed all analyses using SAS® software, Version 9.4 for Windows, using complex survey procedures to account for survey clustering and unequal sampling weights [47].

## Results

For their most recent pregnancy since January 2014 resulting in a live birth, 10.9% (95% CI: 9.0–13.1) of women reported use of herbs during pregnancy and/or labor. Of the 382 women who used herbs during pregnancy and/or labor, the most common reasons reported included stomach pain (42.9%), fetal health (25.5%), miscarriage avoidance (21.6%), and inducing or sustaining labor (12.2%). Among these same women, 17.8% used herbs for no more than 1 day, 41.1% 1–2 weeks, 14.1% between 1 to 2 months, and 21.2% between 3 to 9 months.

Bivariate analyses indicated that use of herbs during pregnancy and/or labor varied by a number of characteristics, including age group (*p* = 0.003), wealth tercile (*p* < 0.001), parity (*p* < 0.001), and place of delivery (*p* < 0.001). Herb use during pregnancy and/or labor did not differ significantly across groups by residence, education level, receipt of ANC, and marital status (Table 1).

**Table 1:** Association of selected characteristics with local herb use during pregnancy and/or labor in Kigoma Region, 2014–2016

Characteristic	Herb use (%)	95% CI *	Live Births	*p*-value**
Four or mo*r*e antenatal care visits *** ^†^				
Yes	10.3	(8.3–12.7)	1973	0.319
No	11.4	(9.2–14.1)	1525	
Residence				
Urban	8.2	(6.0–11.1)	491	0.078
Rural	11.3	(9.2–13.9)	3039	
Age group (yr)				
< 25	13.4	(10.9–16.3)	1396	0.003
25–34	9.2	(7.1–11.8)	1440	
35–49	9.3	(6.7–12.9)	694	
Education level				
None	10.6	(8.2–13.6)	1034	0.856
Some primary	11.7	(8.5–15.8)	555	
Completed primary and/or higher	10.8	(8.6–13.4)	1941	
Marital status				
Currently in union	10.5	(8.6–12.7)	3060	0.104
Previously in union	14.1	(10.3–19)	332	
Never in union	12.1	(7.8–18.4)	138	
Wealth tercile				
Low	13.7	(10.9–17.1)	1278	< 0.001
Middle	9.1	(7.1–11.6)	1165	
High	9.4	(7.5–11.9)	1087	
Birth order				
First	15.3	(12.4–18.7)	669	< 0.001
Second	10.2	(7.6–13.7)	583	
Third or More	9.7	(7.6–12.3)	2278	
Place of delivery				
Hospital, health center, dispensary	9.5	(7.6–11.8)	2099	< 0.001
Home	13.7	(10.7–17.4)	1322	
Other/Unknown	3.3	(1.2–8.7)	109	
**Total** ^**††**^	10.9	(9.0–13.1)	3530	

* CI = Confidence Interval

** Rao-Scott Chi Square

*** Women were counted as having received antenatal care (ANC) care if she made 4 or more ANC visits based on World Health Organization guidelines

^†^ 33 missing values

^††^ 1 missing value

Table 2 summarizes the full and reduced multivariable models for the association of selected characteristics with LHU during pregnancy and/or labor. The final reduced model indicated the odds of LHU during pregnancy and/or labor were higher for women reporting home versus facility-based delivery (aOR: 1.6, 95% CI: 1.1–2.2), having one versus multiple prior live births (aOR: 1.8, 95% CI: 1.4–2.4), and belonging to the lowest as compared to the highest household wealth tercile (aOR: 1.4, 95% CI: 1.0–1.9).

**Table 2:** Summary of factors associated with local herb use during pregnancy and/or labor in Kigoma Region, 2014–2016

Characteristic	Full Model		Final Model	
	Adjusted Odds Ratio (95% CI*)	Type III *p*-value	Adjusted Odds Ratio (95% CI*)	Type III *p*-value
Antenatal Care				
Yes	Reference	0.5461		
No	0.9 (0.7–1.2)			
Age group (yr)				
< 25	Reference	0.5114		
25–34	0.8 (0.5–1.2)			
35–49	0.8 (0.5–1.3)			
Wealth tercile				
Low	1.4 (1.1–1.9)	0.0128	1.4 (1.0–1.9)	< 0.001
Middle	0.9 (0.7–1.2)		0.9 (0.7–1.3)	
High	Reference		Reference	
Birth order				
First	1.5 (1.0–2.3)	0.0290	1.8 (1.4–2.4)	0.01
Second	0.9 (0.7–1.3)		1.0 (0.7–1.5)	
Third or More	Reference		Reference	
Place of delivery				
Hospital, health center, dispensary	Reference	0.0006	Reference	< 0.001
Home	1.5 (1.1–2.1)		1.6 (1.1–2.2)	
Other/Unknown	0.4 (0.1–1.0)		0.4 (0.1–1.0)	

Note: Only the significance associations in the bivariate analyses were included in the full model

**Fig. 1:**
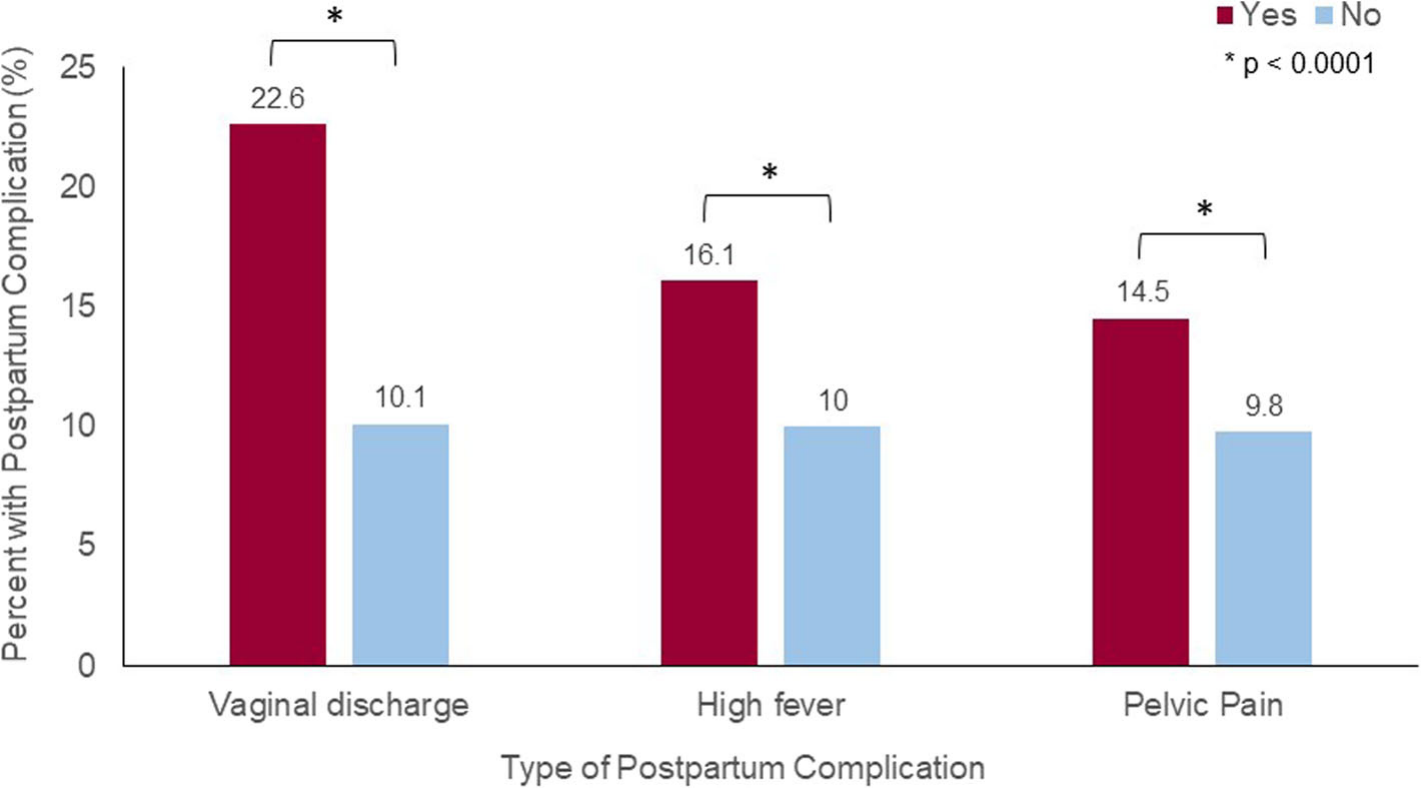
Association of selected clinical postnatal complications with local herb use during pregnancy and/or labor in Kigoma Region, 2014–2016

In the bivariate analyses, having a postnatal obstetric complication varied by whether or not a woman reported LHU during pregnancy and/or labor (*p* = 0.001), as well as by whether she reported receiving the recommended number of ANC visits (*p* < 0.001) (Table 3). We found no significant difference in the experience of postnatal obstetric complications by residence, birth order, delivery location, wealth tercile, age group, or educational level (Table 3). Additionally, LHU was higher among women who reported postnatal abnormal vaginal discharge (22.6%; 95% CI: 16.5–30.2; *p* = < 0. 001), high fever (16.1%; 95% CI: 12.6–20.3; *p* = < 0. 001), and pelvic pain (14.5%; 95% CI: 11.9–17.6; *p* = < 0.001) compared with those that did not use LHU during pregnancy and/or labor (Fig. 1).

**Table 3:** Association of selected characteristics with postnatal complications during pregnancy and/or labor in Kigoma Region, 2014–2016

Characteristic	Postnatal Complications (%)	95% CI *	Live Births	*p*-value**
Local herb use^†^				
Yes	43.1	(37.6–48.7)	382	0.001
No	34.3	(31.8–36.8)	3148	
Four or more antenatal care visits^††^				
Yes	39.3	(29.4–35.1)	1973	< 0.001
No	32.2	(36.0–42.5)	1526	
Residence				
Urban	38.5	(32.8–44.4)	492	0.240
Rural	34.7	(32.0–37.5)	3039	
Age group (yr)				
< 25	33.3	(30.4–36.4)	1396	0.061
25–34	35.5	(32.3–38.8)	1440	
35–49	38.7	(34.5–43.2)	695	
Education level				
None	35.4	(31.8–39.2)	1034	0.728
Some primary	36.8	(31.6–42.3)	556	
Completed primary and/or higher	34.7	(32.0–37.6)	1941	
Marital status				
Currently in union	34.9	(32.3–37.6)	3061	0.397
Previously in union	38.9	(32.7–45.6)	332	
Never in union	33.8	(26.3–42.3)	138	
Wealth tercile				
Low	36.1	(32.4–39.9)	1278	0.613
Middle	44.0	(30.7–37.4)	1165	
High	35.6	(32.0–39.4)	1088	
Parity				
First	31.7	(28.1–35.6)	669	0.117
Second	35.4	(31.2–39.9)	583	
Third or More	36.2	(33.3–39.3)	2279	
Place of delivery				
Hospital, health center, dispensary	34.7	(32.0–37.5)	2100	0.698
Home	36.3	(32.3–40.5)	1322	
Other/Unknown	33.9	(25.6–43.4)	109	
**Total**	35.2	(32.8–37.8)	3531	

* CI = Confidence Interval

** Rao-Scott Chi Square

^†^ 1 missing value

^††^ 33 missing values

In multivariable models the odds of having a postnatal complication was higher among women who reported LHU during pregnancy and/or labor versus those who did not (aOR: 1.5, 95% CI: 1.2–1.9), who had four or more ANC visits during pregnancy compared to those who did not (aOR: 1.4, 95% CI: 1.2–1.6) and were between the ages of 25–34 (aOR: 1.1, 95% CI: 1.0–1.3) or 35–49 (aOR: 1.3, 95% CI: 1.0–1.6) compared with the youngest age group < 25 (Table 4).

**Table 4:** Summary of factors associated with complications among pregnant women in Kigoma Region, 2014–2016

		Final Model	
Characteristic		Adjusted Odds Ratio (95% CI*)	Type III *p*-value
Local herb use			
	Yes	1.5 (1.2–1.9)	0.0012
	No	Reference	
Four or more antenatal care visits			
	Yes	1.4 (1.2–1.6)	< 0.0001
	No	Reference	
Age group (yr)			
	< 25	Reference	0.0447
	25–34	1.1 (1.0–1.3)	
	35–49	1.3 (1.0–1.6)	

Note: Only the significance associations in the bivariate analyses were included in the full model

## Discussion

This study examined the prevalence and characteristics of women with recent lives births who used local herbs during pregnancy and/or labor in Kigoma, Tanzania, as well as tested for associations between LHU during pregnancy and/or labor and postnatal obstetric complications.

Our study found lower reports of local herb use during pregnancy and/or labor among pregnant sub-Saharan African women than previously reported in the literature. Estimates from other studies have found the prevalence of traditional medicine in maternity care among African women to be as high as 80% [48]. One available study on the use of herbal products among pregnant women attending antenatal clinics in a rural district of Tanzania found a prevalence rate of 40.2% [20]. Studies conducted in clinical settings are not representative and may include more women who are seeking care because they are experiencing obstetric-related complications. Our study may undercount LHU during pregnancy due to the potential for recall and social desirability bias. However, this is the first study in SSA that documents LHU in a large and representative sample of women with recent births.

Consistent with other studies, LHU during pregnancy and/or delivery was higher among women with no prior births. These studies have shown that being a primigravida is associated with herbal use during pregnancy [20, 29, 34]. Primigravidas may lack information on the potential risks of herbal use in pregnancy. Communicating the health consequences of herbs used during pregnancy is limited due to the lack of empirical evidence needed to inform underlying health messages. These women may be prone to listen to family and/or friends that recommend herbal medicines during pregnancy, especially in regions where there are limited medical options for health care during pregnancy [29]. Furthermore, it is possible that the accessibility and availability may impact use of herbal products in resource-limited settings, particularly for home deliveries.

We found that the odds of using local herbs were the highest among women who belonged to the lowest household wealth group and had a home delivery; however, we also found a lack of association between LHU and several key sociodemographic factors. We did not find women’s educational level to be associated with LHU as was reported in a number of previous studies [49–53]. Similarly, we did not find factors associated with patterns of herb use during pregnancy related to marital status, or the gestational age of index pregnancy [34, 48, 54].

An association between LHU and postnatal obstetric complications at the population level in a representative sample of pregnant women was not previously documented in Tanzania. In our multivariable model, we found that postnatal obstetric complications were higher among women who were older than the age of 25, took herbs during pregnancy and/or labor, and completed four or more ANC visits during pregnancy. Women that experience pregnancy complications may consult in greater frequency alternative medicine practitioners during pregnancy [55]. More frequent ANC use may be associated with pregnancy complications that continue to manifest in postnatal period. Our study, however, cannot address a temporal association between the onset of and quantity ANC, complications, and herb use during pregnancy due to its cross-sectional design.

There are several key methodological limitations when considering these findings. First, elucidating patterns in deleterious health-related behaviors is often complex and may require special studies to explore contextual factors, such as the role of culture and tradition [56–60] and the interplay between individual, household and community characteristics. Though we recognize these factors are important, the study was not designed to explore these factors. Second, survey respondents were asked to provide information about past events and experiences going as far back as two years and nine months prior to the time of interview and thus, their reports may be subject to recall bias due to varying recall lengths. Steps were taken to improve data quality and mitigate measurement errors, including training interviewers to allow participants sufficient time for adequate recall of long-term memory, to ask prompting questions in relation to local or seasonal events, and to encourage responses in the local language to reduce poor understanding and communication between the interviewer and respondent. Also, women who experienced complications may be more likely to recall and report substances used during pregnancy. Third, social desirability bias or the tendency of respondents to provide answers they believe are more socially acceptable than responses that reflect their true behaviors may have also introduced measurement errors when participants were asked about their history of LHU during pregnancy [61]. Fourth, uterotonic potency has been shown by studies to vary depending on the plant-type as well as the preparation of herbs, quantity consumed, and frequency of intake [62–64]. Our study did not collect these details of use and we cannot explore further an association between LHU and experience of postnatal complications. How varying types, dosage, frequency and combination of herbs, affect pregnancy, delivery or postnatal complications remains unknown. Lastly, the survey did not collect data on patterns of LHU among women who did not carry the pregnancy out to term; due to this limitation in our data, we were not able to examine the associations between herb use during pregnancy and other pregnancy outcomes. Future studies may also consider examining associations between LHU and other markers of postnatal complications such as postnatal hospital re-admissions, blood transfusions, and antibiotic treatments, as well as more detailed accounts about puerperium and postnatal problems.

## Conclusion

Traditional medicine continues to play a significant role in maternal behaviors and experiences across SSA, including Tanzania. In Kigoma Region, approximately one in ten women used local herbs during their last pregnancy and/or labor and the use was associated with having postnatal obstetric complications. While all women need accurate information on the potential risk of herb use during pregnancy and/or labor that is communicated clearly and culturally accessible, tailored messaging may be needed among women more likely to use herbs during pregnancy surrounding the varying degrees of risk depending on the type of herb, how it is prepared and consumed, as well as the frequency of intake. Understanding why women rely on LHU in relation with their characteristics may help identify challenges and barriers surrounding the utilization of maternal health services. Further assessment of specific local herbs used, period and dosage taken, and the properties of the herbs is still needed to clarify the pharmacological safety and efficacy of the specific local herbs used by pregnant women in Kigoma Region.

## Supplementary information

**Additional file 1.** The 2016 Reproductive Health Survey Kigoma Region Individual Questionaire asked information about a woman's background characteristics, contraceptive behaviors and use, fertility, and detailed information about the most recent births.

## Data Availability

Data may be obtained from a third party and are not publicly available. All data that support the findings of this study are archived in the libraries of the CDC country office. While the data were not approved to be made publicly available by NIMR, there are standard procedures in place for other researchers to request the data that could be used with the permission of the Government of Tanzania. Requests for permission to access data can be submitted to NIMR to ensure these data are appropriate for the use sought, will be used consistent with any applicable legal restrictions on the data, and will be used for an appropriate purpose.

## Abbreviation

ANC: Antenatal care
aOR: Adjusted odds ratios
CDC: Centers for Disease Control and Prevention
CI: Confidence interval
HIV/AIDS: Human immunodeficiency virus infection/acquired immune deficiency syndrome
LHU: Local herb use
MoHCDGEC: Ministry of Health, Community Development, Gender, the Elderly and Children, formerly known as Ministry of Health and Welfare
NIMR: National Institute for Medical Research
Ob/Gyn: Obstetrician/Gynecologist
RCH: Reproductive and Child Health
RHS: Reproductive Health Survey
SSA: Sub-Saharan Africa
WHO: World Health Organization
