# Supplementary material for: Factors associated with local herb use during pregnancy and labor among women in Kigoma region, Tanzania, 2014–2016
Source: BMC Pregnancy Childbirth. 2020 Feb 21;20:122. doi: 10.1186/s12884-020-2735-3 (PMC7035699; doi:10.1186/s12884-020-2735-3)
Supplement: Supplementary file 1 — Additional file 1. The 2016 Reproductive Health Survey Kigoma Region Individual Questionaire asked information about a woman's background characteristics, contraceptive behaviors and use, fertility, and detailed information about the most recent births. [file 12884_2020_2735_MOESM1_ESM.pdf]

2016 REPRODUCTIVE HEALTH SURVEY
KIGOMA REGION
INDIVIDUAL QUESTIONNAIRE

Household Questionnaire # ☐☐☐☐ **Woman ID** ☐☐

TIME THE INTERVIEW STARTED: ☐☐ **HOUR (24 HOURS)** ☐☐ **MIN (60 MINUTES)**

SECTION I – RESPONDENT'S BACKGROUND

**101.**	In what month and year were you born?	☐☐ MONTH ☐☐☐☐ YEAR<br>88. DON'T KNOW MONTH 8888. DON'T KNOW YEAR
**102.**	How old were you on your last birthday?	☐☐ YEARS
**103.**	Have you ever attended school?	1. YES<br>2. NO → **Q105**
**104.**	What is the highest level of school you attended?	1. PREPRIMARY<br>2. PRIMARY<br>3. POST-PRIMARY TRAINING<br>4. SECONDARY<br>5. POST-SECONDARY TRAINING<br>6. UNIVERSITY
**104A**.	What is the highest grade you completed at that level?<br>**IF COMPLETED LESS THAN ONE GRADE AT THAT LEVEL, RECORD '00'.**	☐☐ GRADE
**104B.**	Are you still attending school?	1. YES → **Q105**<br>2. NO
**104C.**	How old were you when you last attended school?	☐☐ YEARS<br>88. DON'T KNOW
**105.**	How often do you attend religious services?	1. SEVERAL TIMES A DAY<br>2. ONCE A DAY<br>3. AT LEAST ONCE A WEEK<br>4. AT LEAST ONCE A MONTH<br>5. LESS THAN ONCE A MONTH<br>6. ONLY FOR SPECIAL OCCASIONS<br>7. DOES NOT ATTEND AT ALL<br>9. REFUSED
**106A**.	Aside from your household chores and work, did you do any work outside the home in the past 12 months for which you received money?	1. YES<br>2. NO
**106B.**	Aside from your household chores and work, did you do any work outside the home in the past 12 months for which you were paid in goods?<br>**IF BOTH Q106A <u>AND</u> Q106B = 2, SKIP TO Q108A.**	1. YES<br>2. NO

**107.** Would you say that the money that <u>you</u> earn covers none, some, or all/most of your household expenses?	1. NONE<br>2. SOME<br>3. ALL/MOST
**108A.** Now I would like to ask you some questions about your work and the ownership of goods in your household. Do you have any cash savings of your own?	1. YES<br>2. NO
**108B.** Do you have any land that is owned by you alone?	1. YES<br>2. NO
**108C.** Do you own any assets that could help you generate income?	1. YES<br>2. NO
**111.** How often do you listen to the radio?	1. ALMOST EVERY DAY<br>2. AT LEAST ONCE A WEEK<br>3. LESS THAN ONCE A WEEK<br>4. NOT AT ALL
**112.** How often do you watch television?	1. ALMOST EVERY DAY<br>2. AT LEAST ONCE A WEEK<br>3. LESS THAN ONCE A WEEK<br>4. NOT AT ALL

SECTION II – RELATIONSHIP STATUS AND PARTNERSHIP HISTORY

Now, I would like to ask you some questions about your steady relationships.

**201.** Are you currently married or living with a man as if married?	1. YES, CURRENTLY MARRIED → **Q203A**<br>2. YES, LIVING WITH A MAN → **Q203A**<br>3. NO, NOT IN UNION
**202.** Have you ever been married or lived together with a man as if married?	1. YES, FORMERLY MARRIED<br>2. YES, LIVED WITH A MAN<br>3. NO → **SECTION III**
**203.** What is your marital status now: are you widowed, divorced, or separated?	1. WIDOWED<br>2. DIVORCED<br>3. SEPARATED<br>} **Q207**
**203A.** Does your husband/partner have other wives or does he live with other women as if married?	1. YES<br>2. NO → **Q204**<br>8. DON'T KNOW → **Q204**
**203B.** Including yourself, in total, how many wives or partners does your husband live with now as if married?	[ ][ ] TOTAL NUMBER OF WIVES AND LIVE-IN PARTNERS<br>98. DON'T KNOW
**203C.** Are you the first, second, …wife?	RANK [ ][ ]
**204**. In what month and year did you start living with your (husband/partner)?	[ ][ ] MONTH [ ][ ][ ][ ] YEAR<br>88. DOES NOT REMEMBER MONTH<br>8888. DOES NOT REMEMBER YEAR
**205.** How old were you when you first started living with him?	[ ][ ] YEARS<br>88. DOES NOT REMEMBER<br>99. REFUSED

205A. Now I would like to ask you about who usually makes decisions in your household. Would this person be: you, your husband/partner, both you and your husband partner together, your husband/partner's parents, your own parents, or someone else?

Which member of your household usually makes decisions about: **(READ A-F)**

	WOMAN ONLY	HUSBAND/ PARTNER ONLY	BOTH WOMAN & HUSBAND/ PARTNER	HUSBAND/ PARTNER'S PARENTS	WOMAN'S PARENTS	SOMEONE ELSE	NA
A. Your health care?	1	2	3	4	5	6	9
B. Making large household purchases?	1	2	3	4	5	6	9
C. Making household purchases for daily needs?	1	2	3	4	5	6	9
D. How to use the money that you bring into the household?	1	2	3	4	5	6	9
E. How to use the money that your partner brings into the household?	1	2	3	4	5	6	9
F. Whether you are allowed to work to earn money?	1	2	3	4	5	6	9

**206.** Have you been married or lived with a man only once or more than once?	1. ONLY ONCE → **GO TO SECTION III**<br>2. MORE THAN ONCE
**207**. In what month and year did you start your first legal or common-law marriage?	[ ][ ] MONTH [ ][ ][ ][ ] YEAR<br>88. DOES NOT REMEMBER MONTH<br>8888. DOES NOT REMEMBER YEAR
**208.** How old were you when you first started living with him?	[ ][ ] YEARS<br>88. DOES NOT REMEMBER<br>99. REFUSED

SECTION III – FERTILITY

Now, we are going to talk about your history of menstruation and your pregnancy history. Some of the questions may not apply to you. In these cases, just say so.

**301.** How old were you when your first period (menstruation) started?<br><br>(PROBE: woman's monthly bleeding)	[ ][ ] YEARS<br><br>77. NEVER HAD A PERIOD → **Q303**
**302.** How long has it been since your last menstrual period?	[ ][ ] MONTHS<br><br>00. UP TO ONE MONTH<br>55. CURRENTLY HAVING A PERIOD<br>66. DO NOT HAVE A PERIOD ANY MORE<br>77. BEFORE LAST/ CURRENT PREGNANCY<br>88. DON'T REMEMBER
**303.** Are you currently pregnant?	1. YES<br>2. NO → **Q308**<br>8. NOT SURE → **Q308**
**304.** How many months pregnant are you now?	[ ][ ] MONTHS<br><br>88. NOT SURE
**305.** Just before you got pregnant, did you want to get pregnant then, did you want to get pregnant later, or did you not want to get pregnant then or any time in the future?	1. WANTED THE PREGNANCY THEN<br>2. WANTED THE PREGNANCY LATER<br>3. DID NOT WANT THE PREGNANCY<br>8. NOT SURE
**306.** Is this your first pregnancy?	1. YES<br>2. NO → **Q310**<br>8. NOT SURE
**307.** Have you ever had a stillbirth, ectopic pregnancy, miscarriage, or an induced abortion?	1. YES → **Q315**<br>2. NO → **SECTION V**
**308.** Have you ever been pregnant?	1. YES → **Q310**<br>2. NO<br>8. NOT SURE
**309.** Have you ever had a stillbirth, ectopic pregnancy, miscarriage, or an induced abortion?	1. YES → **Q315**<br>2. NO → **SECTION V**
**310.** How many children have you given birth to who live with you now?	[ ][ ] CHILDREN
**311.** How many children have you given birth to who live somewhere else?	[ ][ ] CHILDREN
**312.** Have you ever had a child born alive who later died, including those who may have died in the first hours or days after birth?	1. YES<br>2. NO → **Q314**
**313.** How many children were born alive but later died?	[ ][ ] CHILDREN
**314.** So altogether you had a total of (**ADD NUMBER OF CHILDREN FROM Q310+Q311+Q313)** live births?	[ ][ ] LIVE BIRTHS

PREGNANCY HISTORY

Now I would like to talk to you about all your pregnancies (not counting the current one). Please, make sure you include all pregnancies, it doesn't matter when they happened or how they ended, whether in a live birth, a stillbirth, an ectopic pregnancy, an abortion, or a miscarriage. Starting with your most recent pregnancy, please give me the following information:

#	**315**<br>How did that pregnancy end?	**316**<br>When did that pregnancy end? (month & year)	**317**<br>How many weeks or months had you been pregnant when that pregnancy ended?	**318**<br>**IF 315=1, 2, or 3**<br>What is the name of the child?	**319**<br>Was (NAME) a boy or a girl?	**320**<br>Is (NAME) still alive?	**321**<br>How old was (NAME) when he/she died? (RECORD DAYS IF LESS THAN 1 MONTH; MONTHS IF LESS THAN TWO YEARS; OR YRS.)
**1**	1. LIVE BIRTH (SINGLE)<br>2. MULTIPLE LIVE BIRTH<br>3. MULTIPLE (LB WITH SB)<br>4. STILLBIRTH (SINGLE)<br>5. MULTIPLE STILLBIRTH<br>6. MISCARRIAGE/ABORTION<br>7. ECTOPIC PREGNANCY	__ __ MTH<br>__ __ __ __YR<br>99. NR	1. ___ ___ WKS<br>OR<br>2. ___ ___ MTHS<br>8. 8 8 DNK<br>9. 9 9 NR/REF<br>**IF Q315=4,5,6, OR 7 GO TO NEXT ROW**	**IF 315=1, 2, or 3, RECORD NAME.**<br>____________	1. BOY<br>2. GIRL	1.YES-> **GO TO NEXT ROW**<br>2. NO	1. ___ ___ DAYS<br>2. ___ ___ MTHS<br>3. ___ ___ YEARS<br>9. 9 9 NR/REF
				**IF Q315=2 RECORD 2ND TWIN NAME.**<br>____________	**IF Q315=2**<br>2nd Twin:<br>1. BOY<br>2. GIRL	2nd Twin:<br>1.YES-> **GO TO NEXT ROW**<br>2. NO	1. ___ ___ DAYS<br>2. ___ ___ MTHS<br>3. ___ ___ YEARS<br>9. 9 9 NR/REF
**2**	1. LIVE BIRTH (SINGLE)<br>2. MULTIPLE LIVE BIRTH<br>3. MULTIPLE (LB WITH SB)<br>4. STILLBIRTH (SINGLE)<br>5. MULTIPLE STILLBIRTH<br>6. MISCARRIAGE/ABORTION<br>7. ECTOPIC PREGNANCY	__ __ MTH<br>__ __ __ __YR<br>99. NR	1. ___ ___ WKS<br>OR<br>2. ___ ___ MTHS<br>8. 8 8 DNK<br>9. 9 9 NR/REF<br>**IF Q315=4,5,6, OR 7 GO TO NEXT ROW**	**IF 315=1, 2, or 3, RECORD NAME.**<br>____________	1. BOY<br>2. GIRL	1.YES-> **GO TO NEXT ROW**<br>2. NO	1. ___ ___ DAYS<br>2. ___ ___ MTHS<br>3. ___ ___ YEARS<br>9. 9 9 NR/REF
				**IF Q315=2 RECORD 2ND TWIN NAME.**<br>____________	**IF Q315=2**<br>2nd Twin:<br>1. BOY<br>2. GIRL	2nd Twin:<br>1.YES-> **GO TO NEXT ROW**<br>2. NO	1. ___ ___ DAYS<br>2. ___ ___ MTHS<br>3. ___ ___ YEARS<br>9. 9 9 NR/REF
**3**	1. LIVE BIRTH (SINGLE)<br>2. MULTIPLE LIVE BIRTH<br>3. MULTIPLE (LB WITH SB)<br>4. STILLBIRTH (SINGLE)<br>5. MULTIPLE STILLBIRTH<br>6. MISCARRIAGE/ABORTION<br>7. ECTOPIC PREGNANCY	__ __ MTH<br>__ __ __ __YR<br>99. NR	1. ___ ___ WKS<br>OR<br>2. ___ ___ MTHS<br>8. 8 8 DNK<br>9. 9 9 NR/REF<br>**IF Q315=4,5,6, OR 7 GO TO NEXT ROW**	**IF 315=1, 2, or 3, RECORD NAME.**<br>____________	1. BOY<br>2. GIRL	1.YES-> **GO TO NEXT ROW**<br>2. NO	1. ___ ___ DAYS<br>2. ___ ___ MTHS<br>3. ___ ___ YEARS<br>9. 9 9 NR/REF
				**IF Q315=2 RECORD 2ND TWIN NAME.**<br>____________	**IF Q315=2**<br>2nd Twin:<br>1. BOY<br>2. GIRL	2nd Twin:<br>1.YES-> **GO TO NEXT ROW**<br>2. NO	1. ___ ___ DAYS<br>2. ___ ___ MTHS<br>3. ___ ___ YEARS<br>9. 9 9 NR/REF
**4**	1. LIVE BIRTH (SINGLE)<br>2. MULTIPLE LIVE BIRTH<br>3. MULTIPLE (LB WITH SB)<br>4. STILLBIRTH (SINGLE)<br>5. MULTIPLE STILLBIRTH<br>6. MISCARRIAGE/ABORTION<br>7. ECTOPIC PREGNANCY	__ __ MTH<br>__ __ __ __YR<br>99. NR	1. ___ ___ WKS<br>OR<br>2. ___ ___ MTHS<br>8. 8 8 DNK<br>9. 9 9 NR/REF<br>**IF Q315=4,5,6, OR 7 GO TO NEXT ROW**	**IF 315=1, 2, or 3, RECORD NAME.**<br>____________	1. BOY<br>2. GIRL	1.YES-> **GO TO NEXT ROW**<br>2. NO	1. ___ ___ DAYS<br>2. ___ ___ MTHS<br>3. ___ ___ YEARS<br>9. 9 9 NR/REF
				**IF Q315=2 RECORD 2ND TWIN NAME.**<br>____________	**IF Q315=2**<br>2nd Twin:<br>1. BOY<br>2. GIRL	2nd Twin:<br>1.YES-> **GO TO NEXT ROW**<br>2. NO	1. ___ ___ DAYS<br>2. ___ ___ MTHS<br>3. ___ ___ YEARS<br>9. 9 9 NR/REF

#	315<br>How did that pregnancy end?	316<br>When did that pregnancy end? (month & year)	317<br>How many weeks or months had you been pregnant when that pregnancy ended?	318<br>IF 315=1, 2, or 3<br>What is the name of the child?	319<br>Was (NAME) a boy or a girl?	320<br>Is (NAME) still alive?	321<br>How old was (NAME) when he/she died? (RECORD DAYS IF LESS THAN 1 MONTH; MONTHS IF LESS THAN TWO YEARS; OR YRS.)
**5**	1. LIVE BIRTH (SINGLE)<br>2. MULTIPLE LIVE BIRTH<br>3. MULTIPLE (LB WITH SB)<br>4. STILLBIRTH (SINGLE)<br>5. MULTIPLE STILLBIRTH<br>6. MISCARRIAGE/ABORTION<br>7. ECTOPIC PREGNANCY	__ __ MTH<br><br>__ __ __ __YR<br><br>99. NR	1. ___ ___ WKS<br>OR<br>2. ___ ___ MTHS<br>8. 8 8 DNK<br>9. 9 9 NR/REF<br>**IF Q315=4,5,6, OR 7 GO TO NEXT ROW**	**IF 315=1, 2, or 3, RECORD NAME.**<br>____________	1. BOY<br>2. GIRL	1.YES-> **GO TO NEXT ROW**<br>2. NO	1. ___ ___ DAYS<br>2. ___ ___ MTHS<br>3. ___ ___ YEARS<br>9. 9 9 NR/REF
				**IF Q315=2 RECORD 2ND TWIN NAME.**<br>____________	**IF Q315=2**<br>2nd Twin:<br>1. BOY<br>2. GIRL	2nd Twin:<br>1.YES-> **GO TO NEXT ROW**<br>2. NO	1. ___ ___ DAYS<br>2. ___ ___ MTHS<br>3. ___ ___ YEARS<br>9. 9 9 NR/REF
**6**	1. LIVE BIRTH (SINGLE)<br>2. MULTIPLE LIVE BIRTH<br>3. MULTIPLE (LB WITH SB)<br>4. STILLBIRTH (SINGLE)<br>5. MULTIPLE STILLBIRTH<br>6. MISCARRIAGE/ABORTION<br>7. ECTOPIC PREGNANCY	__ __ MTH<br><br>__ __ __ __YR<br><br>99. NR	1. ___ ___ WKS<br>OR<br>2. ___ ___ MTHS<br>8. 8 8 DNK<br>9. 9 9 NR/REF<br>**IF Q315=4,5,6, OR 7 GO TO NEXT ROW**	**IF 315=1, 2, or 3, RECORD NAME.**<br>____________	1. BOY<br>2. GIRL	1.YES-> **GO TO NEXT ROW**<br>2. NO	1. ___ ___ DAYS<br>2. ___ ___ MTHS<br>3. ___ ___ YEARS<br>9. 9 9 NR/REF
				**IF Q315=2 RECORD 2ND TWIN NAME.**<br>____________	**IF Q315=2**<br>2nd Twin:<br>1. BOY<br>2. GIRL	2nd Twin:<br>1.YES-> **GO TO NEXT ROW**<br>2. NO	1. ___ ___ DAYS<br>2. ___ ___ MTHS<br>3. ___ ___ YEARS<br>9. 9 9 NR/REF
**7**	1. LIVE BIRTH (SINGLE)<br>2. MULTIPLE LIVE BIRTH<br>3. MULTIPLE (LB WITH SB)<br>4. STILLBIRTH (SINGLE)<br>5. MULTIPLE STILLBIRTH<br>6. MISCARRIAGE/ABORTION<br>7. ECTOPIC PREGNANCY	__ __ MTH<br><br>__ __ __ __YR<br><br>99. NR	1. ___ ___ WKS<br>OR<br>2. ___ ___ MTHS<br>8. 8 8 DNK<br>9. 9 9 NR/REF<br>**IF Q315=4,5,6, OR 7 GO TO NEXT ROW**	**IF 315=1, 2, or 3, RECORD NAME.**<br>____________	1. BOY<br>2. GIRL	1.YES-> **GO TO NEXT ROW**<br>2. NO	1. ___ ___ DAYS<br>2. ___ ___ MTHS<br>3. ___ ___ YEARS<br>9. 9 9 NR/REF
				**IF Q315=2 RECORD 2ND TWIN NAME.**<br>____________	**IF Q315=2**<br>2nd Twin:<br>1. BOY<br>2. GIRL	2nd Twin:<br>1.YES-> **GO TO NEXT ROW**<br>2. NO	1. ___ ___ DAYS<br>2. ___ ___ MTHS<br>3. ___ ___ YEARS<br>9. 9 9 NR/REF
**8**	1. LIVE BIRTH (SINGLE)<br>2. MULTIPLE LIVE BIRTH<br>3. MULTIPLE (LB WITH SB)<br>4. STILLBIRTH (SINGLE)<br>5. MULTIPLE STILLBIRTH<br>6. MISCARRIAGE/ABORTION<br>7. ECTOPIC PREGNANCY	__ __ MTH<br><br>__ __ __ __YR<br><br>99. NR	1. ___ ___ WKS<br>OR<br>2. ___ ___ MTHS<br>8. 8 8 DNK<br>9. 9 9 NR/REF<br>**IF Q315=4,5,6, OR 7 GO TO NEXT ROW**	**IF 315=1, 2, or 3, RECORD NAME.**<br>____________	1. BOY<br>2. GIRL	1.YES-> **GO TO NEXT ROW**<br>2. NO	1. ___ ___ DAYS<br>2. ___ ___ MTHS<br>3. ___ ___ YEARS<br>9. 9 9 NR/REF
				**IF Q315=2 RECORD 2ND TWIN NAME.**<br>____________	**IF Q315=2**<br>2nd Twin:<br>1. BOY<br>2. GIRL	2nd Twin:<br>1.YES-> **GO TO NEXT ROW**<br>2. NO	1. ___ ___ DAYS<br>2. ___ ___ MTHS<br>3. ___ ___ YEARS<br>9. 9 9 NR/REF

#	315<br>How did that pregnancy end?	316<br>When did that pregnancy end? (month & year)	317<br>How many weeks or months had you been pregnant when that pregnancy ended?	318<br>**IF 315=1, 2, or 3**<br>What is the name of the child?	319<br>Was (NAME) a boy or a girl?	320<br>Is (NAME) still alive?	321<br>How old was (NAME) when he/she died? (RECORD DAYS IF LESS THAN 1 MONTH; MONTHS IF LESS THAN TWO YEARS; OR YRS.)
9	1. LIVE BIRTH (SINGLE)<br>2. MULTIPLE LIVE BIRTH<br>3. MULTIPLE (LB WITH SB)<br>4. STILLBIRTH (SINGLE)<br>5. MULTIPLE STILLBIRTH<br>6. MISCARRIAGE/ABORTION<br>7. ECTOPIC PREGNANCY	__ __ MTH<br><br>__ __ __ __YR<br><br>99. NR	1. ___ ___ WKS<br>OR<br>2. ___ ___ MTHS<br>8. 8 8 DNK<br>9. 9 9 NR/REF<br>**IF Q315=4,5,6, OR 7 GO TO NEXT ROW**	**IF 315=1, 2, or 3, RECORD NAME.**<br>____________	1. BOY<br>2. GIRL	1.YES-> **GO TO NEXT ROW**<br>2. NO	1. ___ ___ DAYS<br>2. ___ ___ MTHS<br>3. ___ ___ YEARS<br>9. 9 9 NR/REF
				**IF Q315=2 RECORD 2ND TWIN NAME.**<br>____________	**IF Q315=2**<br>2nd Twin:<br>1. BOY<br>2. GIRL	2nd Twin:<br>1.YES-> **GO TO NEXT ROW**<br>2. NO	1. ___ ___ DAYS<br>2. ___ ___ MTHS<br>3. ___ ___ YEARS<br>9. 9 9 NR/REF
10	1. LIVE BIRTH (SINGLE)<br>2. MULTIPLE LIVE BIRTH<br>3. MULTIPLE (LB WITH SB)<br>4. STILLBIRTH (SINGLE)<br>5. MULTIPLE STILLBIRTH<br>6. MISCARRIAGE/ABORTION<br>7. ECTOPIC PREGNANCY	__ __ MTH<br><br>__ __ __ __YR<br><br>99. NR	1. ___ ___ WKS<br>OR<br>2. ___ ___ MTHS<br>8. 8 8 DNK<br>9. 9 9 NR/REF<br>**IF Q315=4,5,6, OR 7 GO TO NEXT ROW**	**IF 315=1, 2, or 3, RECORD NAME.**<br>____________	1. BOY<br>2. GIRL	1.YES-> **GO TO NEXT ROW**<br>2. NO	1. ___ ___ DAYS<br>2. ___ ___ MTHS<br>3. ___ ___ YEARS<br>9. 9 9 NR/REF
				**IF Q315=2 RECORD 2ND TWIN NAME.**<br>____________	**IF Q315=2**<br>2nd Twin:<br>1. BOY<br>2. GIRL	2nd Twin:<br>1.YES-> **GO TO NEXT ROW**<br>2. NO	1. ___ ___ DAYS<br>2. ___ ___ MTHS<br>3. ___ ___ YEARS<br>9. 9 9 NR/REF
11	1. LIVE BIRTH (SINGLE)<br>2. MULTIPLE LIVE BIRTH<br>3. MULTIPLE (LB WITH SB)<br>4. STILLBIRTH (SINGLE)<br>5. MULTIPLE STILLBIRTH<br>6. MISCARRIAGE/ABORTION<br>7. ECTOPIC PREGNANCY	__ __ MTH<br><br>__ __ __ __YR<br><br>99. NR	1. ___ ___ WKS<br>OR<br>2. ___ ___ MTHS<br>8. 8 8 DNK<br>9. 9 9 NR/REF<br>**IF Q315=4,5,6, OR 7 GO TO NEXT ROW**	**IF 315=1, 2, or 3, RECORD NAME.**<br>____________	1. BOY<br>2. GIRL	1.YES-> **GO TO NEXT ROW**<br>2. NO	1. ___ ___ DAYS<br>2. ___ ___ MTHS<br>3. ___ ___ YEARS<br>9. 9 9 NR/REF
				**IF Q315=2 RECORD 2ND TWIN NAME.**<br>____________	**IF Q315=2**<br>2nd Twin:<br>1. BOY<br>2. GIRL	2nd Twin:<br>1.YES-> **GO TO NEXT ROW**<br>2. NO	1. ___ ___ DAYS<br>2. ___ ___ MTHS<br>3. ___ ___ YEARS<br>9. 9 9 NR/REF
12	1. LIVE BIRTH (SINGLE)<br>2. MULTIPLE LIVE BIRTH<br>3. MULTIPLE (LB WITH SB)<br>4. STILLBIRTH (SINGLE)<br>5. MULTIPLE STILLBIRTH<br>6. MISCARRIAGE/ABORTION<br>7. ECTOPIC PREGNANCY	__ __ MTH<br><br>__ __ __ __YR<br><br>99. NR	1. ___ ___ WKS<br>OR<br>2. ___ ___ MTHS<br>8. 8 8 DNK<br>9. 9 9 NR/REF<br>**IF Q315=4,5,6, OR 7 GO TO NEXT ROW**	**IF 315=1, 2, or 3, RECORD NAME.**<br>____________	1. BOY<br>2. GIRL	1.YES-> **GO TO NEXT ROW**<br>2. NO	1. ___ ___ DAYS<br>2. ___ ___ MTHS<br>3. ___ ___ YEARS<br>9. 9 9 NR/REF
				**IF 315=1, 2, or 3, RECORD NAME.**<br>____________	1. BOY<br>2. GIRL	1.YES-> **GO TO NEXT ROW**<br>2. NO	1. ___ ___ DAYS<br>2. ___ ___ MTHS<br>3. ___ ___ YEARS<br>9. 9 9 NR/REF

#	315<br>How did that pregnancy end?	316<br>When did that pregnancy end? (month & year)	317<br>How many weeks or months had you been pregnant when that pregnancy ended?	318<br>**IF 315=1, 2, or 3**<br>What is the name of the child?	319<br>Was (NAME) a boy or a girl?	320<br>Is (NAME) still alive?	321<br>How old was (NAME) when he/she died? (RECORD DAYS IF LESS THAN 1 MONTH; MONTHS IF LESS THAN TWO YEARS; OR YRS.)
**13**	1. LIVE BIRTH (SINGLE)<br>2. MULTIPLE LIVE BIRTH<br>3. MULTIPLE (LB WITH SB)<br>4. STILLBIRTH (SINGLE)<br>5. MULTIPLE STILLBIRTH<br>6. MISCARRIAGE/ABORTION<br>7. ECTOPIC PREGNANCY	__ __ MTH<br>__ __ __ __YR<br>99. NR	1. ___ ___ WKS<br>OR<br>2. ___ ___ MTHS<br>8. 8 8 DNK<br>9. 9 9 NR/REF<br>**IF Q315=4,5,6, OR 7 GO TO NEXT ROW**	**IF 315=1, 2, or 3, RECORD NAME.**<br>____________	1. BOY<br>2. GIRL	1.YES-> **GO TO NEXT ROW**<br>2. NO	1. ___ ___ DAYS<br>2. ___ ___ MTHS<br>3. ___ ___ YEARS<br>9. 9 9 NR/REF
				**IF Q315=2 RECORD 2ND TWIN NAME.**<br>____________	**IF Q315=2**<br>2nd Twin:<br>1. BOY<br>2. GIRL	2nd Twin:<br>1.YES-> **GO TO NEXT ROW**<br>2. NO	1. ___ ___ DAYS<br>2. ___ ___ MTHS<br>3. ___ ___ YEARS<br>9. 9 9 NR/REF
**14**	1. LIVE BIRTH (SINGLE)<br>2. MULTIPLE LIVE BIRTH<br>3. MULTIPLE (LB WITH SB)<br>4. STILLBIRTH (SINGLE)<br>5. MULTIPLE STILLBIRTH<br>6. MISCARRIAGE/ABORTION<br>7. ECTOPIC PREGNANCY	__ __ MTH<br>__ __ __ __YR<br>99. NR	1. ___ ___ WKS<br>OR<br>2. ___ ___ MTHS<br>8. 8 8 DNK<br>9. 9 9 NR/REF<br>**IF Q315=4,5,6, OR 7 GO TO NEXT ROW**	**IF 315=1, 2, or 3, RECORD NAME.**<br>____________	1. BOY<br>2. GIRL	1.YES-> **GO TO NEXT ROW**<br>2. NO	1. ___ ___ DAYS<br>2. ___ ___ MTHS<br>3. ___ ___ YEARS<br>9. 9 9 NR/REF
				**IF Q315=2 RECORD 2ND TWIN NAME.**<br>____________	**IF Q315=2**<br>2nd Twin:<br>1. BOY<br>2. GIRL	2nd Twin:<br>1.YES-> **GO TO NEXT ROW**<br>2. NO	1. ___ ___ DAYS<br>2. ___ ___ MTHS<br>3. ___ ___ YEARS<br>9. 9 9 NR/REF
**15**	1. LIVE BIRTH (SINGLE)<br>2. MULTIPLE LIVE BIRTH<br>3. MULTIPLE (LB WITH SB)<br>4. STILLBIRTH (SINGLE)<br>5. MULTIPLE STILLBIRTH<br>6. MISCARRIAGE/ABORTION<br>7. ECTOPIC PREGNANCY	__ __ MTH<br>__ __ __ __YR<br>99. NR	1. ___ ___ WKS<br>OR<br>2. ___ ___ MTHS<br>8. 8 8 DNK<br>9. 9 9 NR/REF<br>**IF Q315=4,5,6, OR 7 GO TO NEXT ROW**	**IF 315=1, 2, or 3, RECORD NAME.**<br>____________	1. BOY<br>2. GIRL	1.YES-> **GO TO NEXT ROW**<br>2. NO	1. ___ ___ DAYS<br>2. ___ ___ MTHS<br>3. ___ ___ YEARS<br>9. 9 9 NR/REF
				**IF Q315=2 RECORD 2ND TWIN NAME.**<br>____________	**IF Q315=2**<br>2nd Twin:<br>1. BOY<br>2. GIRL	2nd Twin:<br>1.YES-> **GO TO NEXT ROW**<br>2. NO	1. ___ ___ DAYS<br>2. ___ ___ MTHS<br>3. ___ ___ YEARS<br>9. 9 9 NR/REF
**16**	1. LIVE BIRTH (SINGLE)<br>2. MULTIPLE LIVE BIRTH<br>3. MULTIPLE (LB WITH SB)<br>4. STILLBIRTH (SINGLE)<br>5. MULTIPLE STILLBIRTH<br>6. MISCARRIAGE/ABORTION<br>7. ECTOPIC PREGNANCY	__ __ MTH<br>__ __ __ __YR<br>99. NR	1. ___ ___ WKS<br>OR<br>2. ___ ___ MTHS<br>8. 8 8 DNK<br>9. 9 9 NR/REF<br>**IF Q315=4,5,6, OR 7 GO TO NEXT ROW**	**IF 315=1, 2, or 3, RECORD NAME.**<br>____________	1. BOY<br>2. GIRL	1.YES-> **GO TO NEXT ROW**<br>2. NO	1. ___ ___ DAYS<br>2. ___ ___ MTHS<br>3. ___ ___ YEARS<br>9. 9 9 NR/REF
				**IF Q315=2 RECORD 2ND TWIN NAME.**<br>____________	1. BOY<br>2. GIRL	1.YES-> **GO TO NEXT ROW**<br>2. NO	1. ___ ___ DAYS<br>2. ___ ___ MTHS<br>3. ___ ___ YEARS<br>9. 9 9 NR/REF

#	315	316	317	318	319	320	321
	How did that pregnancy end?	When did that pregnancy end? (month & year)	How many weeks or months had you been pregnant when that pregnancy ended?	**IF 315=1, 2, or 3**<br>What is the name of the child?	Was (NAME) a boy or a girl?	Is (NAME) still alive?	How old was (NAME) when he/she died? (RECORD DAYS IF LESS THAN 1 MONTH; MONTHS IF LESS THAN TWO YEARS; OR YRS.)
**17**	1. LIVE BIRTH (SINGLE)<br>2. MULTIPLE LIVE BIRTH<br>3. MULTIPLE (LB WITH SB)<br>4. STILLBIRTH (SINGLE)<br>5. MULTIPLE STILLBIRTH<br>6. MISCARRIAGE/ABORTION<br>7. ECTOPIC PREGNANCY	__ __ MTH<br>__ __ __ __YR<br>99. NR	1. ___ ___ WKS<br>OR<br>2. ___ ___ MTHS<br>8. 8 8 DNK<br>9. 9 9 NR/REF<br>**IF Q315=4,5,6, OR 7 GO TO NEXT ROW**	**IF 315=1, 2, or 3, RECORD NAME.**<br>___________	1. BOY<br>2. GIRL	1.YES-> **GO TO NEXT ROW**<br>2. NO	1. ___ ___ DAYS<br>2. ___ ___ MTHS<br>3. ___ ___ YEARS<br>9. 9 9 NR/REF
				**IF Q315=2 RECORD 2ND TWIN NAME.**<br>___________	**IF Q315=2**<br>2nd Twin:<br>1. BOY<br>2. GIRL	2nd Twin:<br>1.YES-> **GO TO NEXT ROW**<br>2. NO	1. ___ ___ DAYS<br>2. ___ ___ MTHS<br>3. ___ ___ YEARS<br>9. 9 9 NR/REF
**18**	1. LIVE BIRTH (SINGLE)<br>2. MULTIPLE LIVE BIRTH<br>3. MULTIPLE (LB WITH SB)<br>4. STILLBIRTH (SINGLE)<br>5. MULTIPLE STILLBIRTH<br>6. MISCARRIAGE/ABORTION<br>7. ECTOPIC PREGNANCY	__ __ MTH<br>__ __ __ __YR<br>99. NR	1. ___ ___ WKS<br>OR<br>2. ___ ___ MTHS<br>8. 8 8 DNK<br>9. 9 9 NR/REF<br>**IF Q315=4,5,6, OR 7 GO TO NEXT ROW**	**IF 315=1, 2, or 3, RECORD NAME.**<br>___________	1. BOY<br>2. GIRL	1.YES-> **GO TO NEXT ROW**<br>2. NO	1. ___ ___ DAYS<br>2. ___ ___ MTHS<br>3. ___ ___ YEARS<br>9. 9 9 NR/REF
				**IF Q315=2 RECORD 2ND TWIN NAME.**<br>___________	**IF Q315=2**<br>2nd Twin:<br>1. BOY<br>2. GIRL	2nd Twin:<br>1.YES-> **GO TO NEXT ROW**<br>2. NO	1. ___ ___ DAYS<br>2. ___ ___ MTHS<br>3. ___ ___ YEARS<br>9. 9 9 NR/REF
**19**	1. LIVE BIRTH (SINGLE)<br>2. MULTIPLE LIVE BIRTH<br>3. MULTIPLE (LB WITH SB)<br>4. STILLBIRTH (SINGLE)<br>5. MULTIPLE STILLBIRTH<br>6. MISCARRIAGE/ABORTION<br>7. ECTOPIC PREGNANCY	__ __ MTH<br>__ __ __ __YR<br>99. NR	1. ___ ___ WKS<br>OR<br>2. ___ ___ MTHS<br>8. 8 8 DNK<br>9. 9 9 NR/REF<br>**IF Q315=4,5,6, OR 7 GO TO NEXT ROW**	**IF 315=1, 2, or 3, RECORD NAME.**<br>___________	1. BOY<br>2. GIRL	1.YES-> **GO TO NEXT ROW**<br>2. NO	1. ___ ___ DAYS<br>2. ___ ___ MTHS<br>3. ___ ___ YEARS<br>9. 9 9 NR/REF
				**IF Q315=2 RECORD 2ND TWIN NAME.**<br>___________	**IF Q315=2**<br>2nd Twin:<br>1. BOY<br>2. GIRL	2nd Twin:<br>1.YES-> **GO TO NEXT ROW**<br>2. NO	1. ___ ___ DAYS<br>2. ___ ___ MTHS<br>3. ___ ___ YEARS<br>9. 9 9 NR/REF
**20**	1. LIVE BIRTH (SINGLE)<br>2. MULTIPLE LIVE BIRTH<br>3. MULTIPLE (LB WITH SB)<br>4. STILLBIRTH (SINGLE)<br>5. MULTIPLE STILLBIRTH<br>6. MISCARRIAGE/ABORTION<br>7. ECTOPIC PREGNANCY	__ __ MTH<br>__ __ __ __YR<br>99. NR	1. ___ ___ WKS<br>OR<br>2. ___ ___ MTHS<br>8. 8 8 DNK<br>9. 9 9 NR/REF<br>**IF Q315=4,5,6, OR 7 GO TO NEXT ROW**	**IF 315=1, 2, or 3, RECORD NAME.**<br>___________	1. BOY<br>2. GIRL	1.YES-> **GO TO NEXT ROW**<br>2. NO	1. ___ ___ DAYS<br>2. ___ ___ MTHS<br>3. ___ ___ YEARS<br>9. 9 9 NR/REF
				**IF Q315=2 RECORD 2ND TWIN NAME.**<br>9<br>___________	1. BOY<br>2. GIRL	1.YES-> **GO TO NEXT ROW**<br>2. NO	1. ___ ___ DAYS<br>2. ___ ___ MTHS<br>3. ___ ___ YEARS<br>9. 9 9 NR/REF

SECTION IV: HEALTH CARE FOR BIRTHS SINCE JANUARY 2014
LAST BIRTH

**400.**	**INTERVIEWER, CHECK THE PREGNANCY HISTORY AND RECORD THE TOTAL NUMBER OF PREGNANCIES THAT ENDED IN EITHER LIVEBIRTHS OR STILLBIRTHS (Q315= 1,2,3,4, or 5) SINCE JANUARY 2014.**<br><br>**INTERVIEWER: THIS IS THE TOTAL NUMBER OF PREGNANCIES YOU SHOULD REGISTER IN SECTION IV (UP TO 3.)**	**1. TOTAL PREGNANCIES ENDING IN LIVEBIRTHS OR STILLBIRTHS SINCE JANUARY 2014** ☐<br><br>**2. IF NONE SINCE JANUARY 2014 → SECTION V**
**401.**	**COPY THE PREGNANCY NUMBER AND OUTCOME OF THE <u>LAST BIRTH</u> SINCE JANUARY 2014 ON PAGE 5**	**PREGNANCY # FOR LAST BIRTH** ☐<br>**PREGNANCY OUTCOME CODE:**<br>**"1", "2", "3", "4, or "5"** ☐<br><br>**IF CODE= 1, 2, or 3 FOR LAST BIRTH, RECORD:**<br>**NAME:** ____________________<br>**STILL ALIVE?**<br>**(RECORD IF Q320=1 FOR LAST BIRTH)**<br>1. YES<br>2. NO
**402.**	Now, I would like to talk to you about your last birth. <u>Just before you got pregnant</u>, did you want to get pregnant then, did you want to get pregnant later, or did you not want to get pregnant then or any time in the future?	1. WANTED THE PREGNANCY THEN<br>2. WANTED THE PREGNANCY LATER<br>3. DID NOT WANT THE PREGNANCY<br>8. NOT SURE
**405.**	Were you given any injection to prevent the baby from getting tetanus (i.e., *lock jaw*) during that pregnancy?	1. YES - FOR TETANUS<br>2. YES - DON'T KNOW WHAT FOR<br>3. NO → **Q407**
**406.**	How many injections were given?	☐ INJECTIONS<br>8. NOT SURE
**407.**	Did you see anyone for antenatal care during that pregnancy?	1. YES → **Q408**<br>2. NO
**407A.**	Why didn't you go for antenatal care?<br><br>PROBE: Any other reason?<br><br>**RECORD ALL MENTIONED, THEN GO TO Q414A**	A. COST TOO MUCH<br>B. FACILITY FAR AWAY<br>C. TRANSPORTATION UNAVAILABLE/EXPENSIVE<br>D. DON'T TRUST FACILITY/POOR QUALITY SERVICE<br>E. NO FEMALE PROVIDER AT FACILITY<br>F. HUSBAND/FAMILY DID NOT ALLOW<br>G. NOT NECESSARY<br>H. NOT CUSTOMARY<br>I. OTHER _________________<br><br>**GO TO Q414A**

**408.** Where did you go for antenatal care?<br>PROBE: Anywhere else?<br><br>**RECORD ALL MENTIONED.**	**KIGOMA HOSPITAL CODES:**<br><br>**BAPTIST: 128**<br>**HERI: 36**<br>**KABANGA: 48**<br>**KASULU (MUMANI): 41**<br>**KIBONDO: 96**<br>**MAWENI: 15**	**KIGOMA HEALTH CENTER CODES:**<br>**BIHARU: 32**<br>**BITALE: 3**<br>**BUHINGU: 81**<br>**GUNGU (KIKUKU): 10**<br>**GWANUMPU: 86**<br>**ILAGALA: 12**<br>**JANDA: 39**<br>**KAKONKO: 88**<br>**KIFURA: 99**<br>**KIGANAMO: 46**<br>**KIMWANYA: 129**<br>**MABAMBA: 110**<br>**MATYAZO: 14**	**MULERA: 60**<br>**MUYAMA: 63**<br>**MUYOVOZI: 130**<br>**MWAMGONGO: 30**<br>**NGURUKA: 21**<br>**NYWAKITONTO: 70**<br>**NYANZIGE: 118**<br>**NYENGE: 5**<br>**RUSESA: 76**<br>**SHUNGA: 77**<br>**UJIJI: 24**<br>**UVINZA: 27**	A. KIGOMA HOSPITAL: CODE ____ ____ ____<br>B. OTHER HOSPITAL (SPECIFY) _________________<br>C. KIGOMA HEALTH CENTER: CODE ___ ___ ___<br>D. DISPENSARY _____________________________<br>E. HOME<br>F. CBD WORKER<br>G. COMMUNITY HEALTH WORKER (CHW)<br>H. TRAINED BIRTH ATTENDANT<br>I. OTHER (SPECIFY) _____________________________

**409.** How many times did you go?	[ ][ ] TIMES<br>66. NOT SURE, BUT AT LEAST 4 TIMES<br>77. NOT SURE<br>88. DOES NOT REMEMBER
**410.** In what month of the pregnancy did the antenatal care begin?	[ ][ ] MONTH<br>88. DOES NOT REMEMBER
**411.** During this pregnancy, were any of the following done at least once:<br>**READ A-E. CIRCLE YES OR NO FOR EACH.**<br>A. Were you weighed?<br>B. Was your height measured?<br>C. Did you give a urine sample?<br>D. Did you give a blood sample?<br>E. Were you tested for HIV?	**YES** / **NO**<br>A. WEIGHT 1 2<br>B. HEIGHT 1 2<br>C. URINE SAMPLE 1 2<br>D. BLOOD SAMPLE 1 2<br>E. HIV TESTED 1 2
**412A.** Was your blood pressure ever checked during that pregnancy?	1. YES<br>2. NO → **Q413A**<br>8. DOES NOT REMEMBER → **Q413A**
**412B.** During this pregnancy, were you told that your blood pressure was high?	1. YES<br>2. NO<br>8. DOES NOT REMEMBER
**413A.** Were you told about the signs of pregnancy complications?	1. YES<br>2. NO<br>8. DOES NOT REMEMBER
**413A2.** In your opinion, what are some of the serious health problems that can occur during pregnancy and around labor and childbirth that could endanger the life of a pregnant woman?<br>PROBE: Any other health problem?<br>**RECORD ALL MENTIONED.**	A. SEVERE VAGINAL BLEEDING<br>B. SWOLLEN HANDS/FACE<br>C. BLURRED VISION<br>D. PROLONGED LABOR (>12 HOURS)<br>E. CONVULSIONS<br>F. RETAINED PLACENTA<br>G. FOUL SMELLING VAGINAL DISCHARGE<br>H. HIGH FEVER<br>I. OTHER (SPECIFY)______________________<br>Z. NONE MENTIONED
**413B.** Were you advised to develop a birth plan?	1. YES<br>2. NO<br>8. DOES NOT REMEMBER
**413B2.** What arrangements did you or your family make for the birth of this child?<br>**READ A-E. CIRCLE YES OR NO FOR EACH.**<br>Did you:<br>A. Identify transport?<br>B. Save money?<br>C. Identify a blood donor?<br>D. Identify a nurse, midwife, or doctor to deliver the baby?<br>E. Identify a companion or support person to accompany you to a facility for delivery?	**YES** / **NO**<br>A. TRANSPORT 1 2<br>B. SAVE MONEY 1 2<br>C. BLOOD DONOR 1 2<br>D. SKILLED ATTENDANT 1 2<br>E. COMPANION / SUPPORT FACILITY 1 2
**414A.** Did you sleep under insecticide-treated bed nets (ITN) most of the time?	1. YES<br>2. NO<br>8. DOES NOT REMEMBER
**414B.** During this pregnancy, did you take SP-Fansidar to keep you from getting malaria?	1. YES<br>2. NO → **Q414D**<br>8. DOES NOT REMEMBER → **Q414D**
**414C.** How many times did you take SP-Fansidar?	[ ][ ] TIMES
**414D.** Did you take any local herbs during your pregnancy and/or labor?	1. YES<br>2. NO → **Q415**<br>8. DOES NOT REMEMBER → **Q415**

**414E.** For what reasons did you take the local herbs?<br><br>PROBE: Any other reason?<br><br>**RECORD ALL MENTIONED**	A. TO INDUCE OR SUSTAIN LABOR (CONTRACTION THAT LED UP TO BIRTH)<br>B. TO TREAT MALARIA<br>C. TO TREAT COLD/FLU<br>D. TO TREAT HEADACHE<br>E. TO TREAT CONVULSIONS<br>F. TO TREAT VAGINAL BLEEDING<br>G. TO TREAT STOMACH PAIN<br>H. FOR THE HEALTH OF THE CHILD<br>I. TO AVOID MISCARRIAGE<br>J. OTHER (SPECIFY) ________________________
**414F.** Altogether, how many days did you take local herbs during the pregnancy and/or labor?<br><br>**IF ANSWER IS GIVEN IN MONTHS, CONVERT IT TO DAYS.**	___ ___ ___ DAYS<br><br>777. CONTINUOUSLY<br>888. DOES NOT REMEMBER
**414G.** In what **month of the pregnancy** did you begin using local herbs?	___ ___ MONTH<br><br>77. JUST BEFORE DELIVERY<br>88. DOES NOT REMEMBER
**414H.** In what **month of the pregnancy** did you stop using local herbs?	___ ___ MONTH<br><br>55. DURING LABOR / JUST BEFORE DELIVERY<br>66. DURING DELIVERY<br>77. AFTER DELIVERY<br>88. DOES NOT REMEMBER
**415. Before you delivered the baby,** how long had you been in labor? By labor, we mean strong, regular and frequent contractions at least 5 minutes apart.	___ ___ HOURS<br>76. MORE THAN 3 DAYS (>72 HOURS)<br>77. C-SECTION BEFORE LABOR<br>88. DOESN'T KNOW

**416A.** Where did you give birth to your last baby?	**KIGOMA HOSPITAL CODES:**<br><br>**BAPTIST: 128**<br>**HERI: 36**<br>**KABANGA: 48**<br>**KASULU (MUMANI): 41**<br>**KIBONDO: 96**<br>**MAWENI: 15**	**KIGOMA HEALTH CENTER CODES:**<br><br>**BIHARU: 32**<br>**BITALE: 3**<br>**BUHINGU: 81**<br>**GUNGU (KIKUKU): 10**<br>**GWANUMPU: 86**<br>**ILAGALA: 12**<br>**JANDA: 39**<br>**KAKONKO: 88**<br>**KIFURA: 99**<br>**KIGANAMO: 46**<br>**KIMWANYA: 129**<br>**MABAMBA: 110**<br>**MATYAZO: 14**<br>**MULERA: 60**<br>**MUYAMA: 63**<br>**MUYOVOZI: 130**<br>**MWAMGONGO: 30**<br>**NGURUKA: 21**<br>**NYWAKITONTO: 70**<br>**NYANZIGE: 118**<br>**NYENGE: 5**<br>**RUSESA: 76**<br>**SHUNGA: 77**<br>**UJIJI: 24**<br>**UVINZA: 27**	1. KIGOMA HOSPITAL: CODE ___ ___ ___<br>2. OTHER HOSPITAL (SPECIFY): ______________<br>3. KIGOMA HEALTH CENTER: CODE ___ ___ ___<br>4. DISPENSARY (SPECIFY): _________________<br>5. ON THE WAY TO A HEALTH FACILITY → **Q419B**<br>6. OWN HOME → **Q419A**<br>7. OTHER HOME → **Q419A**<br>8. OTHER (SPECIFY) _________________ → **Q419A**

**416A1.** Would you recommend this health facility to a friend or family member for maternal health services?	1. YES<br>2. NO<br>8. DON'T KNOW
**416A2.** Was this the closest health facility to your home?	1. YES → **Q416B**<br>2. NO<br>8. DON'T KNOW → **Q416B**
**416A3.** What are the reasons you did not go to the facility nearest to your home?<br><br>PROBE: "Anything else?"<br><br>**RECORD ALL MENTIONED**	A. INCONVENIENT OPERATING HOURS<br>B. BAD REPUTATION<br>C. DON'T LIKE STAFF<br>D. NO MEDICINE<br>E. PREFERS TO REMAIN ANONYMOUS (NO ONE KNOWS ME AT THIS FACILITY)<br>F. IT IS MORE EXPENSIVE<br>G. REFERRED FROM ANOTHER FACILITY<br>H. FACILITY NOT OPEN<br>I. FACILITY OF POOR QUALITY<br>J. FACILITY DOES NOT PROVIDE DESIRED SERVICES<br>K. PROVIDERS OFTEN AWAY<br>L. FACILITY DOES NOT ACCEPT INSURANCE<br>M. PROVIDER TREATS PATIENTS UNKINDLY<br>N. BAD EXPERIENCE AT THE FACILITY IN THE PAST<br>O. OTHER (SPECIFY) _________________________<br>Y. DON'T KNOW / REMEMBER

**416B.** Were you referred?	1. YES<br>2. NO
**417A.** How long did it take you, one-way, to get from your home to the health facility where you delivered?	1. ___ ___ MINUTES<br><br>OR<br><br>2. ___ ___ HOURS<br><br>888. DON'T REMEMBER
**417B.** How did you get to the health facility?<br><br>PROBE: Any other way?<br><br>**RECORD ALL MENTIONED**	A. WALKED / ON FOOT<br>B. AMBULANCE BICYCLE<br>C. AMBULANCE MOTORCYCLE<br>D. AMBULANCE CAR / TRUCK<br>E. SIMPLE BICYCLE<br>F. SIMPLE MOTORCYCLE<br>G. SIMPLE CAR / TRUCK<br>H. BOAT<br>I. BUS / MINIBUS<br>J. BAJAJ<br>K. CART<br>L. LITTER (HOMEMADE GURNEY)<br>M. OTHER (SPECIFY) _______________
**417C.** How much did you pay for transport to the facility, in total?	___ ___ ___ ___ ___ ___ TSH<br><br>000000. NONE<br><br>777777. PAID IN-KIND<br><br>888888. DOESN'T KNOW
**417D.** Did you pay any formal or informal fees for deliver care for your last birth?	1. YES<br>2. NO → **Q417G**
**417E.** How much did you pay in <u>formal fees</u> for delivery care services at the facility?	___ ___ ___ ___ ___ ___ TSH<br><br>000000. NONE<br><br>888888. DOESN'T KNOW
**417F.** How much did you pay in <u>informal fees</u> for delivery care services at the facility?	___ ___ ___ ___ ___ ___ TSH<br><br>000000. NONE<br><br>888888. DOESN'T KNOW
**417G.** Did the facility provide supplies for childbirth?	1. YES<br>2. NO → **Q417I**
**417H.** How much did you pay for supplies at the facility?	___ ___ ___ ___ ___ ___ TSH<br><br>000000. NONE<br><br>777777. PAID IN-KIND<br><br>888888. DOESN'T KNOW
**417I.** How much did you pay for any supplies that you brought?	___ ___ ___ ___ ___ ___ TSH<br><br>000000. NONE<br><br>777777. PAID IN-KIND<br><br>888888. DOESN'T KNOW
**417J.** How much did you pay for any medications?	___ ___ ___ ___ ___ ___ TSH<br><br>000000. NONE<br><br>777777. PAID IN-KIND<br><br>888888. DOESN'T KNOW

**417K.** How much did you pay for accommodation near the facility?	___ ___ ___ ___ ___ ___ TSH<br>000000. NONE<br>777777. PAID IN-KIND<br>888888. DOESN'T KNOW
**417L.** How much did you pay for food at the facility?	___ ___ ___ ___ ___ ___ TSH<br>000000. NONE<br>777777. PAID IN-KIND<br>888888. DOESN'T KNOW
**417M.** How much did you pay for care for your dependents back home?	___ ___ ___ ___ ___ ___ TSH<br>000000. NONE<br>777777. PAID IN-KIND<br>888888. DOESN'T KNOW
**417N.** How much did you pay for any other expenses related to your last delivery? (SPECIFY OTHER EXPENSES)<br>______________________________	___ ___ ___ ___ ___ ___ TSH<br>000000. NONE<br>777777. PAID IN-KIND<br>888888. DOESN'T KNOW
**417N2.** Did you give gifts or any non-monetary payments for services during your last delivery?	1. YES<br>2. NO<br>8. DOESN'T KNOW / REMEMBER
**417O.** Thinking back to the way staff treated you during your last delivery, would you say you were satisfied, somewhat satisfied, or not satisfied?	1. SATISFIED<br>2. SOMEWHAT SATISFIED<br>3. NOT SATISFIED<br>8. DOESN'T KNOW / REMEMBER

417P. When you came to the facility for delivery, did the staff….

READ A-L. CIRCLE YES OR NO FOR EACH.

Question		YES	NO
A. Care for you with a kind approach?	A. KIND APPROACH	1	2
B. Treat you in a friendly manner?	B. FRIENDLY MANNER	1	2
C. Show concern and empathy?	C. CONCERN/EMPATHY	1	2
D. Treat you with respect as an individual?	D. TREAT WITH RESPECT	1	2
E. Call you by my name?	E. CALL BY NAME	1	2
F. Respond to your needs whether or not you asked?	F. RESPOND TO NEEDS	1	2
G. Slap you during delivery for different reasons?	G. SLAP DURING DELIV.	1	2
H. Shout at you because you had not done what you were told?	H. SHOUT	1	2
I. Keep you waiting for a long time before receiving service?	I. WAITING LONG TIME	1	2
J. Allow you to practice cultural rituals in the facility?	J. ALLOW RITUALS	1	2
K. Delay service provision due to health facility's internal problems?	K. DELAY SERVICE	1	2
L. Not treat you well because of your personal attributes?	L. NOT TREAT WELL	1	2

**418.** Was the baby delivered by caesarean section; that is, did they cut your belly open to take the baby out?	1. YES<br>2. NO
**418A.** Did you have a companion or support person accompany you to the facility where you delivered?	1. YES<br>2. NO → **Q418F**<br>8. DOESN'T KNOW / REMEMBER → **Q418F**

418B. Was your companion or support person allowed to:

Question		YES	NO
A. Be with you during labor?	A. DURING LABOR	1	2
B. Be with you during delivery?	B. DURING DELIVERY	1	2
C. Be with you after delivery?	C. AFTER DELIVERY	1	2

**418C.** Who was your companion or support person?<br><br>PROBE: Anyone else?<br><br>**RECORD ALL MENTIONED.**	A. HUSBAND OR PARTNER<br>B. MOTHER<br>C. SISTER<br>D. MOTHER-IN-LAW<br>E. OTHER RELATIVE<br>F. TRADITIONAL BIRTH ATTENDANT<br>G. NEIGHBOR<br>H. FRIEND<br>I. OTHER (SPECIFY) ____________________
**418F. INTERVIEWER: DID THE DELIVER OCCUR IN A FACILITY? CHECK Q416A = 1, 2, 3, OR 4.**	**1. YES → Q419B**<br>**2. NO → CONTINUE**
**419A.** Why did you decide to not deliver in a health facility?<br><br>PROBE: Any other reason?<br><br>**RECORD ALL MENTIONED**	A. COST TOO MUCH TO DELIVER AT FACILITY<br>B. FACILITY NOT OPEN<br>C. FACILITY FAR AWAY<br>D. TRANSPORTATION UNAVAILABLE<br>E. TRANSPORTATION EXPENSIVE<br>F. DON'T TRUST FACILITY<br>G. EXPECT POOR QUALITY SERVICE/CARE<br>H. FEAR OF ABUSE/DISRESPECT AT FACILITY<br>I. LACK OF ALTERNATIVE CARE FOR FAMILY<br>J. NO ACCOMODATIONS NEAR FACILITY<br>K. NO FEMALE PROVIDER AT FACILITY<br>L. BABY CAME EARLIER THAN EXPECTED<br>M. HUSBAND/FAMILY DID NOT ALLOW<br>N. NOT NECESSARY<br>O. NOT CUSTOMARY<br>P. OTHER (SPECIFY) ____________________
**419A1.** What was the <u>most important</u> reason?<br>**WRITE THE LETTER FOR THE MOST IMPORTANT REASON MENTIONED IN Q419A.**	☐
**419B.** Who made the decision about where to deliver the baby?	1. PREGNANT WOMAN HERSELF<br>2. HUSBAND/PARTNER<br>3. BOTH WOMAN AND HUSBAND/PARTNER<br>4. HUSBAND'S FATHER<br>5. HUSBAND'S MOTHER<br>6. PREGNANT WOMAN'S FATHER<br>7. PREGNANT WOMAN'S MOTHER<br>8. OTHER (SPECIFY) ____________________
**420.** Who assisted with the delivery?<br>PROBE: Anyone else?<br><br>**RECORD ALL MENTIONED. IF RESPONDENT SAYS NO ONE ASSISTED, PROBE TO DETERMINE WHETHER ANY ADULTS WERE PRESENT AT DELIVERY.**	A. DOCTOR / ASSISTANT MEDICAL OFFICER<br>B. CLINICAL OFFICER<br>C. ASSISTANT CLINICAL OFFICER<br>D. TRAINED NURSE / MIDWIFE<br>E. MCH AIDE<br>F. MEDICAL ATTENDANT<br>G. NURSE ASSISTANT<br>H. VILLAGE HEALTH WORKER<br>I. TBA<br>J. RELATIVE/FRIEND<br>K. OTHER (SPECIFY) ____________________<br>L. NO ONE<br>M. DOESN'T KNOW

421. During the <u>first 6 weeks</u> after the birth, did you have any of the following complications?
READ A–K. CIRCLE YES OR NO FOR EACH OPTION.

		<u>YES</u>	<u>NO</u>
A. Severe Bleeding	A. SEVERE BLEEDING	1	2
B. Bad-smelling Vaginal Discharge	B. BAD-SMELLING VAGINAL DISCHARGE	1	2
C. Infection of Surgical Wound	C. INFECTION OF SURGICAL WOUND	1	2
D. Faint/coma	D. FAINT/COMA	1	2
E. High Fever (39-40c)	E. HIGH FEVER (39-40C)	1	2
F. Painful Urination	F. PAINFUL URINATION	1	2
G. Painful Uterus (pelvic pain)	G. PAINFUL UTERUS (PELVIC PAIN)	1	2
H. Breast Infection	H. BREAST INFECTION	1	2
I. Continuous leaking of urine from the vagina	I. CONTINUOUS LEAKING OF URINE FROM VAGINA	1	2
J. Continuous leaking of faeces from the vagina	J. CONTINUOUS LEAKING OF FAECES FROM VAGINA	1	2
K. Any other complication?	K. OTHER (SPECIFY) ______________	1	2

**422.** Did you have any seizures or convulsions while pregnant, in labor or immediately after delivery?<br>**PROBE: Were you told that you had eclampsia?**	1. YES<br>2. NO<br>8. DOES NOT REMEMBER
**423A. REVIEW 416A: IF DELIVERY OCCURRED IN A FACILITY (416A=1-4), ASK:** After you left the facility, did any health care provider or a traditional birth attendant check on your health?<br><br>**IF DELIVERY OCCURRED OUTSIDE A FACILITY (416A=5-8), ASK:** After the baby was born, did any health care provider or a traditional birth attendant check on your health?	1. YES<br>2. NO →**Q424A**<br>8. DOES NOT REMEMBER →**Q424A**
**423B.** How long after delivery did the first check take place?<br><br>**INTERVIEWER: IF 'SAME DAY', PROBE TO BE SURE THE CHECK OCCURRED DURING A SEPARATE VISIT.**	1. ___ ___ DAYS<br>2. ___ ___ WEEKS<br>000. SAME DAY<br>888. DON'T REMEMBER
**423C.** Where did the first check take place?	1. HOSPITAL (SPECIFY) ________________________<br>2. HEALTH CENTER (SPECIFY) ________________<br>3. DISPENSARY (SPECIFY) ______________________<br>4. HOME<br>5. CBD WORKER<br>6. OTHER (SPECIFY) ____________________________
**424A.** At any time during that pregnancy, including after delivery, were you counseled about family planning?	1. YES<br>2. NO → **Q425F**<br>8. DOES NOT KNOW → **Q425F**

**424B.** Did you receive counseling about family planning during…? **READ A-C.**		**YES**	**NO**
A. Antenatal care	A. ANTENATAL CARE	1	2
B. Time of delivery	B. AT THE TIME OF DELIVERY	1	2
C. Postpartum check-up	C. POSTPARTUM CHECK UP	1	2

425F. INTERVIEWER: REVIEW Q401 AND RECORD THE PREGNANCY OUTCOME OF THE LAST BIRTH.

PREGNANCY OUTCOME: ☐

425FF. INTERVIEWER: VERIFY 425F AND PREGNANCY HISTORY ON P.5, AND CIRCLE ONE ANSWER:

1. **IF THE PREGNANCY OUTCOME WAS NOT A LIVE BIRTH (CODE 4, 5, OR 3 IF FIRST TWIN WAS BORN DEAD), SKIP TO Q431.**
2. **IF THE PREGNANCY OUTCOME IS A LIVE BIRTH (CODE 1), CONTINUE.**
3. **IF THE PREGNANCY OUTCOME IS TWINS (BOTH ALIVE) (CODE 2), CONTINUE.**
4. **IF THE PREGNANCY OUTCOME IS TWINS (1 LIVE BIRTH, 1 STILLBIRTH) (CODE 3), CONTINUE IF THIS FIRST TWIN WAS BORN ALIVE (CHECK PREGNANCY HISTORY ON P. 5).**

**426A.** Was (NAME) weighed at birth?	1. YES<br>2. NO → **Q426C**<br>8. DOESN'T KNOW → **Q426C**
**426B.** How much did (NAME) weigh?	____ . _____ KILOGRAMS → **GO TO Q427**<br>88. DON'T KNOW
**426C.** Do you know if (NAME) weighed less than 2.5 kg or was considered too small?	1. YES, WAS LESS THAN 2.5 kg<br>2. NO, WAS MORE THAN 2.5 kg<br>8. DOESN'T KNOW / REMEMBER
**426D.** Immediately after the birth, was (NAME) put directly on the bare skin of your chest?	1. YES<br>2. NO<br>8. DOES NOT KNOW
**426E.** After you delivered, did the facility give you a birth notification form?	1. YES<br>2. NO<br>8. DOES NOT KNOW

**427.** Does (NAME) have a birth certificate?	1. YES<br>2. NO<br>8. DOES NOT KNOW
**428A.** Did you ever breastfeed (NAME)?	1. YES<br>2. NO → **Q429A**
**428B.** How long after birth did you first put (NAME) to the breast?	1. ___ ___ HOURS<br>OR<br>2. ___ ___ DAYS<br>000. WITHIN ONE HOUR<br>888. DO NOT REMEMBER
**428C. CHECK 320: IS THE <u>CHILD FROM THE LAST BIRTH</u> STILL LIVING?**	**1. YES**<br>**2. NO → Q429A**
**428D.** Are you still breastfeeding (NAME)?	1. YES<br>2. NO → **Q428H**
**428E.** Now I would like to ask you about liquids or foods (NAME) had <u>yesterday</u> during the day or night. Did (NAME) drink/eat:<br>**READ A–E. CIRCLE YES OR NO FOR EACH.**	<u>YES</u> <u>NO</u>
A. Plain water?	A. PLAIN WATER 1 2
B. Commercially produced infant formula?	B. FORMULA 1 2
C. Any [BRAND NAME OF COMMERCIALLY FORTIFIED BABY FOOD, e.g. Cerelac]?	C. BABY CEREAL 1 2
D. Any milk from animals?	D. ANIMAL MILK 1 2
E. Any (other) porridge like ugali?	E. PORRIDGE/UGALI 1 2
**428F. INTERVIEWER: CHECK Q428E. DID THE CHILD FROM THE LAST BIRTH HAVE ANY DRINK OR FOOD OTHER THAN BREAST MILK (ANY "YES" IN Q428E)?**	**1. YES → Q429A**<br>**2. NO**
**428G.** Aside from breastmilk, did (NAME) have anything at all to eat or drink yesterday or last night?<br>**RECORD RESPONSE. THEN GO TO Q429A.**	1. YES<br>2. NO<br>8. DOES NOT KNOW } **Q429A**
**428H.** For how many months did you breastfeed (NAME)?	[ ][ ] MONTHS<br>88. DO NOT REMEMBER
**429A.** In the two months after (NAME) was born, did any health care provider or traditional birth attendant check on <u>the baby's</u> health?	1. YES<br>2. NO → **Q431**<br>3. NO, BABY DIED → **Q431**<br>8. DOES NOT REMEMBER → **Q431**
**429B.** How many days or weeks after (NAME's) birth did the first health check take place?	1. ___ ___ DAYS OR<br>2. ___ ___ WEEKS<br>000. SAME DAY<br>888. DO NOT REMEMBER
**430.** Was the health check because (NAME) was sick or was it a routine health exam?	1. HEALTH CHECK FOR SICKNESS<br>2. ROUTINE HEALTH CHECK<br>8. DOES NOT REMEMBER
**431.** How many months after (NAME's) birth did your period (menstruation) return?	[ ][ ] MONTHS<br>66. NOT YET RESUMED
**432.** How many months after (NAME's) birth did you resume sexual relations?	[ ][ ] MONTHS<br>66. NOT YET RESUMED
**433F. INTERVIEWER, WAS THE LAST BIRTH A TWIN BIRTH (Q425F=2, 3, OR 5)?**	**1. YES**<br>**2. NO → Q400_2**
**433FF. INTERVIEWER, WAS THE <u>SECOND TWIN</u> FROM THIS BIRTH BORN ALIVE (Q425F=2, OR Q425F=3 IF SECOND TWIN WAS BORN ALIVE)?**	**1. BOTH TWINS ALIVE**<br>**2. FIRST TWIN DEAD, SECOND TWIN ALIVE**<br>**3. FIRST TWIN ALIVE, SECOND TWIN DEAD → Q400_2**<br>**4. BOTH TWINS DEAD → Q400_2**

**434A.** Now I would like to talk about the second twin, (NAME). Was (NAME) also weighed at birth?	1. YES<br>2. NO → **Q434C**<br>8. DOES NOT KNOW → **Q434C**
**434B.** How much did (NAME) weigh?	____ . ____ KILOGRAMS → **Q435**<br>88. DON'T KNOW
**434C.** Do you know if (NAME) weighed less than 2.5 kg or was considered too small?	1. YES, WAS LESS THAN 2.5 kg<br>2. NO, WAS MORE THAN OR EQUAL TO 2.5 kg<br>8. DOESN'T KNOW / REMEMBER
**434D.** Immediately after the birth, was (NAME) put directly on the bare skin of your chest?	1. YES<br>2. NO<br>8. DOES NOT KNOW
**434E.** After you delivered, did the facility give you a birth notification form?	1. YES<br>2. NO<br>8. DOES NOT KNOW
**435.** Does (NAME) have a birth certificate?	1. YES<br>2. NO<br>8. DOES NOT KNOW
**436A.** Did you ever breastfeed (NAME)?	1. YES<br>2. NO → **Q437A**
**436B.** How long after birth did you first put (NAME) to the breast?	1. ___ ___ HOURS OR<br>2. ___ ___ DAYS<br>000. WITHIN ONE HOUR<br>888. DO NOT REMEMBER
**436C. INTERVIEWER, CHECK Q320: IS THE SECOND TWIN OF THE LAST BIRTH STILL LIVING?**	1. YES<br>2. NO → **Q437A**
**436D.** Are you still breastfeeding (NAME)?	1. YES<br>2. NO → **Q436H**
**436E.** Now I would like to ask you about liquids or foods (NAME) had yesterday during the day or night. Did (NAME) drink/eat: **READ A–E. CIRCLE YES OR NO FOR EACH.**	<u>YES</u> <u>NO</u>
A. Plain water?	A. PLAIN WATER 1 2
B. Commercially produced infant formula?	B. FORMULA 1 2
C. Any milk from animals?	C. ANIMAL MILK 1 2
D. Tea, juice, or other liquids?	D. OTHER LIQUIDS 1 2
E. Any [BRAND NAME OF COMMERCIALLY FORTIFIED BABY FOOD, E.G. Cerelac]?	E. BABY CEREAL 1 2
F. Any (other) porridge like ugali?	F. PORRIDGE/UGALI 1 2
**436F. INTERVIEWER CHECK Q436E: DID THE CHILD FROM THE LAST BIRTH HAVE ANY DRINK OR FOOD OTHER THAN BREAST MILK (ANY "YES" IN Q436E)?**	**1. CHILD HAD ANY OF THE FOOD/DRINK → Q437A**<br>2. **CHILD DID NOT HAVE ANY OF THE FOOD/DRINK → CONTINUE**
**436G.** Aside from breastmilk, did (NAME) have anything at all to eat or drink yesterday or last night?<br>**RECORD RESPONSE. THEN GO TO Q437A.**	1. YES → **Q437A**<br>2. NO → **Q437A**<br>8. DOES NOT KNOW → **Q437A**
**436H.** For how many months did you breastfeed (NAME)?	[ ][ ] MONTHS<br>88. DO NOT REMEMBER
**437A.** In the <u>two months after</u> (NAME) was born, did any health care provider or traditional birth attendant check on his/her health?	1. YES<br>2. NO → **Q400_2**<br>3. NO, BABY DIED → **Q400_2**<br>8. DOES NOT REMEMBER → **Q400_2**
**437B.** How many days or weeks after (NAME's) birth did the first health check take place?	1. ___ ___ DAYS OR<br>2. ___ ___ WEEKS<br>000. SAME DAY<br>888. DO NOT REMEMBER
**438.** Was the health check because (NAME) was sick or was it a routine health exam?	1. HEALTH CHECK FOR SICKNESS<br>2. ROUTINE HEALTH CHECK<br>8. DOES NOT REMEMBER

SECTION IV: HEALTH CARE FOR BIRTHS SINCE JANUARY 2014
SECOND-TO-LAST BIRTH

**400_2.**	**INTERVIEWER, DID ANY SECOND-TO-LAST BIRTH OCCUR SINCE JANUARY 2014 (CHECK Q400 AND Q315)?**	**1. YES**<br>**2. NO → GO TO SECTION V**<br>**3. ONLY ONE BIRTH → GO TO SECTION V**
**401_2.**	**INTERVIEWER, COPY THE PREGNANCY NUMBER AND OUTCOME OF THE SECOND-TO-LAST SINCE JANUARY 2014 ON PAGE 5.**	**PREGNANCY # FOR SECOND-TO-LAST BIRTH:** ☐<br>**PREGNANCY OUTCOME CODE:**<br>**"1", "2", "3", "4, or "5"** ☐<br>**IF CODE= 1, 2, or 3 FOR SECOND-TO-LAST BIRTH, RECORD:**<br>**NAME:** ____________________<br>**STILL ALIVE?**<br>**(RECORD IF Q320=1 FOR SECOND-TO-LAST BIRTH)**<br>1. YES<br>2. NO
**402_2.**	Now, I would like to talk to you about your last birth. Just before you got pregnant, did you want to get pregnant then, did you want to get pregnant later, or did you not want to get pregnant then or any time in the future?	1. WANTED THE PREGNANCY THEN<br>2. WANTED THE PREGNANCY LATER<br>3. DID NOT WANT THE PREGNANCY<br>8. NOT SURE
**405_2.**	Were you given any injection to prevent the baby from getting tetanus (i.e., *lock jaw*) during that pregnancy?	1. YES - FOR TETANUS<br>2. YES - DON'T KNOW WHAT FOR<br>3. NO → **Q407_2**
**406_2.**	How many injections were given?	☐ INJECTIONS<br>8. NOT SURE
**407_2.**	Did you see anyone for antenatal care during that pregnancy?	1. YES → **Q408_2**<br>2. NO
**407A_2.**	Why didn't you go for antenatal care?<br>PROBE: Any other reason?<br>**RECORD ALL MENTIONED, THEN GO TO Q414A**	A. COST TOO MUCH<br>B. FACILITY FAR AWAY<br>C. TRANSPORTATION UNAVAILABLE/EXPENSIVE<br>D. DON'T TRUST FACILITY/POOR QUALITY SERVICE<br>E. NO FEMALE PROVIDER AT FACILITY<br>F. HUSBAND/FAMILY DID NOT ALLOW<br>G. NOT NECESSARY<br>H. NOT CUSTOMARY<br>I. OTHER ________________<br>**GO TO Q414A_2**

**408_2.**	Where did you go for antenatal care?<br>PROBE: Anywhere else?<br>**RECORD ALL MENTIONED.**	**KIGOMA HOSPITAL CODES:**<br>**BAPTIST: 128**<br>**HERI: 36**<br>**KABANGA: 48**<br>**KASULU (MUMANI): 41**<br>**KIBONDO: 96**<br>**MAWENI: 15**	**KIGOMA HEALTH CENTER CODES:**<br>**BIHARU: 32**<br>**BITALE: 3**<br>**BUHINGU: 81**<br>**GUNGU (KIKUKU): 10**<br>**GWANUMPU: 86**<br>**ILAGALA: 12**<br>**JANDA: 39**<br>**KAKONKO: 88**<br>**KIFURA: 99**<br>**KIGANAMO: 46**<br>**KIMWANYA: 129**<br>**MABAMBA: 110**<br>**MATYAZO: 14**<br>**MULERA: 60**<br>**MUYAMA: 63**<br>**MUYOVOZI: 130**<br>**MWAMGONGO: 30**<br>**NGURUKA: 21**<br>**NYWAKITONTO: 70**<br>**NYANZIGE: 118**<br>**NYENGE: 5**<br>**RUSESA: 76**<br>**SHUNGA: 77**<br>**UJIJI: 24**<br>**UVINZA: 27**	A. KIGOMA HOSPITAL: CODE ___ ___ ___<br>B. OTHER HOSPITAL (SPECIFY) ________________<br>C. KIGOMA HEALTH CENTER: CODE ___ ___ ___<br>D. DISPENSARY ___________________________<br>E. HOME<br>F. CBD WORKER<br>G. COMMUNITY HEALTH WORKER (CHW)<br>H. TRAINED BIRTH ATTENDANT<br>I. OTHER (SPECIFY) ___________________________

**409_2.** How many times did you go?	☐☐ TIMES<br><br>66. NOT SURE, BUT AT LEAST 4 TIMES<br>77. NOT SURE<br>88. DOES NOT REMEMBER
**410_2.** In what month of the pregnancy did the antenatal care begin?	☐☐ MONTH<br>88. DOES NOT REMEMBER
**411_2.** During this pregnancy, were any of the following done at least once:<br>**READ A-E. CIRCLE YES OR NO FOR EACH.**<br>A. Were you weighed?<br>B. Was your height measured?<br>C. Did you give a urine sample?<br>D. Did you give a blood sample?<br>E. Were you tested for HIV?	**YES** **NO**<br>A. WEIGHT 1 2<br>B. HEIGHT 1 2<br>C. URINE SAMPLE 1 2<br>D. BLOOD SAMPLE 1 2<br>E. HIV TESTED 1 2
**412A_2.** Was your blood pressure ever checked during that pregnancy?	1. YES<br>2. NO → **Q413A_2**<br>8. DOES NOT REMEMBER → **Q413A_2**
**412B_2.** During this pregnancy, were you told that your blood pressure was high?	1. YES<br>2. NO<br>8. DOES NOT REMEMBER
**413A_2.** Were you told about the signs of pregnancy complications?	1. YES<br>2. NO<br>8. DOES NOT REMEMBER
**413B_2.** Were you advised to develop a birth plan?	1. YES<br>2. NO<br>8. DOES NOT REMEMBER
**413B2_2.** What arrangements did you or your family make for the birth of this child?<br>**READ A-E. CIRCLE YES OR NO FOR EACH.**<br>Did you:<br>A. Identify transport?<br>B. Save money?<br>C. Identify a blood donor?<br>D. Identify a nurse, midwife, or doctor to deliver the baby?<br>E. Identify a companion or support person to accompany you to a facility for delivery?	**YES** **NO**<br>A. TRANSPORT 1 2<br>B. SAVE MONEY 1 2<br>C. BLOOD DONOR 1 2<br>D. SKILLED ATTENDANT 1 2<br>E. COMPANION / SUPPORT FACILITY 1 2
**414A_2.** Did you sleep under insecticide-treated bed nets (ITN) most of the time?	1. YES<br>2. NO<br>8. DOES NOT REMEMBER
**414B_2.** During this pregnancy, did you take SP-Fansidar to keep you from getting malaria?	1. YES<br>2. NO → **Q414D_2**<br>8. DOES NOT REMEMBER → **Q414D_2**
**414C_2.** How many times did you take SP-Fansidar?	☐☐ TIMES
**414D_2.** Did you take any local herbs during your pregnancy and/or labor?	1. YES<br>2. NO → **Q415_2**<br>8. DOES NOT REMEMBER → **Q415_2**
**414E_2.** For what reasons did you take the local herbs?<br><br>PROBE: Any other reason?<br><br>RECORD ALL MENTIONED	A. TO INDUCE OR SUSTAIN LABOR (CONTRACTION THAT LED UP TO BIRTH)<br>B. TO TREAT MALARIA<br>C. TO TREAT COLD/FLU<br>D. TO TREAT HEADACHE<br>E. TO TREAT CONVULSIONS<br>F. TO TREAT VAGINAL BLEEDING<br>G. TO TREAT STOMACH PAIN<br>H. FOR THE HEALTH OF THE CHILD<br>I. TO AVOID MISCARRIAGE<br>J. OTHER (SPECIFY) ________________________
**414F_2.** Altogether, how many days did you take local herbs during the pregnancy and/or labor?<br><br>**IF ANSWER IS GIVEN IN MONTHS, CONVERT IT TO DAYS.**	___ ___ ___ DAYS<br><br>777. CONTINUOUSLY<br>888. DOES NOT REMEMBER

**414G_2.** In what month of the pregnancy did you begin using local herbs?	___ ___ MONTH<br><br>77. JUST BEFORE DELIVERY<br>88. DOES NOT REMEMBER
**414H_2.** In what month of the pregnancy did you stop using local herbs?	___ ___ MONTH<br><br>55. DURING LABOR / JUST BEFORE DELIVERY<br>66. DURING DELIVERY<br>77. AFTER DELIVERY<br>88. DOES NOT REMEMBER
**415_2.** Before you delivered the baby, how long had you been in labor? By labor, we mean strong, regular and frequent contractions at least 5 minutes apart.	___ ___ HOURS<br>76. MORE THAN 3 DAYS (>72 HOURS)<br>77. C-SECTION BEFORE LABOR<br>88. DOESN'T KNOW
**416A_2.** Where did you give birth to this baby?<br><br>**KIGOMA HOSPITAL CODES:**<br>**BAPTIST: 128**<br>**HERI: 36**<br>**KABANGA: 48**<br>**KASULU (MUMANI): 41**<br>**KIBONDO: 96**<br>**MAWENI: 15**<br><br>**KIGOMA HEALTH CENTER CODES:**<br>**BIHARU: 32**<br>**BITALE: 3**<br>**BUHINGU: 81**<br>**GUNGU (KIKUKU): 10**<br>**GWANUMPU: 86**<br>**ILAGALA: 12**<br>**JANDA: 39**<br>**KAKONKO: 88**<br>**KIFURA: 99**<br>**KIGANAMO: 46**<br>**KIMWANYA: 129**<br>**MABAMBA: 110**<br>**MATYAZO: 14**<br>**MULERA: 60**<br>**MUYAMA: 63**<br>**MUYOVOZI: 130**<br>**MWAMGONGO: 30**<br>**NGURUKA: 21**<br>**NYWAKITONTO: 70**<br>**NYANZIGE: 118**<br>**NYENGE: 5**<br>**RUSESA: 76**<br>**SHUNGA: 77**<br>**UJIJI: 24**<br>**UVINZA: 27**	1. KIGOMA HOSPITAL: CODE ___ ___ ___<br>2. OTHER HOSPITAL (SPECIFY) ______________<br>3. KIGOMA HEALTH CENTER: CODE ___ ___ ___<br>4. DISPENSARY (SPECIFY) _________________<br>5. ON THE WAY TO A HEALTH FACILITY → **Q419B_2**<br>6. OWN HOME → **Q419A_2**<br>7. OTHER HOME → **Q419A_2**<br>8. OTHER (SPECIFY) → **Q419A_2**
**416A1_2.** Would you recommend this health facility to a friend or family member for maternal health services?	1. YES<br>2. NO<br>8. DON'T KNOW
**416A2_2.** Was this the closest health facility to your home?	1. YES → **Q416B_2**<br>2. NO<br>8. DON'T KNOW → **Q416B_2**
**416A3_2.** What are the reasons you did not go to the facility nearest to your home?<br><br>PROBE: "Anything else?"<br><br>**RECORD ALL MENTIONED**	A. INCONVENIENT OPERATING HOURS<br>B. BAD REPUTATION<br>C. DON'T LIKE STAFF<br>D. NO MEDICINE<br>E. PREFERS TO REMAIN ANONYMOUS (NO ONE KNOWS ME AT THIS FACILITY)<br>F. IT IS MORE EXPENSIVE<br>G. REFERRED FROM ANOTHER FACILITY<br>H. FACILITY NOT OPEN<br>I. FACILITY OF POOR QUALITY<br>J. FACILITY DOES NOT PROVIDE DESIRED SERVICES<br>K. PROVIDERS OFTEN AWAY<br>L. FACILITY DOES NOT ACCEPT INSURANCE<br>M. PROVIDER TREATS PATIENTS UNKINDLY<br>N. BAD EXPERIENCE AT THE FACILITY IN THE PAST<br>O. OTHER (SPECIFY) _________________________<br>Y. DON'T KNOW / REMEMBER
**416B_2.** Were you referred for the second-to-last-birth?	1. YES<br>2. NO
**417A_2.** How long did it take you, one-way, to get from your home to the health facility where you delivered?	1. ___ ___ MINUTES<br><br>OR<br><br>2. ___ ___ HOURS<br><br>888. DON'T REMEMBER

**417B_2.** How did you get to the health facility?<br><br>PROBE: Any other way?<br><br>**RECORD ALL MENTIONED**	A. WALKED / ON FOOT<br>B. AMBULANCE BICYCLE<br>C. AMBULANCE MOTORCYCLE<br>D. AMBULANCE CAR / TRUCK<br>E. SIMPLE BICYCLE<br>F. SIMPLE MOTORCYCLE<br>G. SIMPLE CAR / TRUCK<br>H. BOAT<br>I. BUS / MINIBUS<br>J. BAJAJ<br>K. CART<br>L. LITTER (HOMEMADE GURNEY)<br>M. OTHER (SPECIFY) ______________
**417C_2.** How much did you pay for transport to the facility, in total?	___ ___ ___ ___ ___ ___ TSH<br><br>000000. NONE<br><br>777777. PAID IN-KIND<br><br>888888. DOESN'T KNOW
**417D_2.** Did you pay any formal or informal fees for deliver care for your second-to-last birth?	1. YES<br>2. NO → **Q417G_2**
**417E_2.** How much did you pay in <u>formal fees</u> for delivery care services at the facility?	___ ___ ___ ___ ___ ___ TSH<br><br>000000. NONE<br><br>888888. DOESN'T KNOW
**417F_2.** How much did you pay in <u>informal fees</u> for delivery care services at the facility?	___ ___ ___ ___ ___ ___ TSH<br><br>000000. NONE<br><br>888888. DOESN'T KNOW
**417G_2.** Did the facility provide supplies for childbirth?	1. YES<br>2. NO → **Q417I_2**
**417H_2.** How much did you pay for supplies at the facility?	___ ___ ___ ___ ___ ___ TSH<br><br>000000. NONE<br><br>777777. PAID IN-KIND<br><br>888888. DOESN'T KNOW
**417I_2.** How much did you pay for any supplies that you brought?	___ ___ ___ ___ ___ ___ TSH<br><br>000000. NONE<br><br>777777. PAID IN-KIND<br><br>888888. DOESN'T KNOW
**417J_2.** How much did you pay for any medications?	___ ___ ___ ___ ___ ___ TSH<br><br>000000. NONE<br><br>777777. PAID IN-KIND<br><br>888888. DOESN'T KNOW
**417K_2.** How much did you pay for accommodation near the facility?	___ ___ ___ ___ ___ ___ TSH<br><br>000000. NONE<br><br>777777. PAID IN-KIND<br><br>888888. DOESN'T KNOW

**417L_2.** How much did you pay for food at the facility?	___ ___ ___ ___ ___ ___ TSH<br>000000. NONE<br>777777. PAID IN-KIND<br>888888. DOESN'T KNOW
**417M_2.** How much did you pay for care for your dependents back home?	___ ___ ___ ___ ___ ___ TSH<br>000000. NONE<br>777777. PAID IN-KIND<br>888888. DOESN'T KNOW
**417N_2.** How much did you pay for any other expenses related to your last delivery? (SPECIFY OTHER EXPENSES)<br>______________________________	___ ___ ___ ___ ___ ___ TSH<br>000000. NONE<br>777777. PAID IN-KIND<br>888888. DOESN'T KNOW
**417N2_2.** Did you give gifts or any non-monetary payments for services during your delivery?	1. YES<br>2. NO<br>8. DOESN'T KNOW / REMEMBER
**417O_2.** Thinking back to the <u>way staff treated you</u> during your second-to-last delivery, would you say you were satisfied, somewhat satisfied, or not satisfied?	1. SATISFIED<br>2. SOMEWHAT SATISFIED<br>3. NOT SATISFIED<br>8. DOESN'T KNOW / REMEMBER

**417P_2.** When you came to the facility for delivery, did the staff…. **READ A-L. CIRCLE YES OR NO FOR EACH.**		**YES**	**NO**
A. Care for you with a kind approach?	A. KIND APPROACH	1	2
B. Treat you in a friendly manner?	B. FRIENDLY MANNER	1	2
C. Show concern and empathy?	C. CONCERN/EMPATHY	1	2
D. Treat you with respect as an individual?	D. TREAT WITH RESPECT	1	2
E. Call you by my name?	E. CALL BY NAME	1	2
F. Respond to your needs whether or not you asked?	F. RESPOND TO NEEDS	1	2
G. Slap you during delivery for different reasons?	G. SLAP DURING DELIV.	1	2
H. Shout at you because you had not done what you were told?	H. SHOUT	1	2
I. Keep you waiting for a long time before receiving service?	I. WAITING LONG TIME	1	2
J. Allow you to practice cultural rituals in the facility?	J. ALLOW RITUALS	1	2
K. Delay service provision due to health facility's internal problems?	K. DELAY SERVICE	1	2
L. Not treat you well because of your personal attributes?	L. NOT TREAT WELL	1	2

**418_2.** Was the baby delivered by caesarean section; that is, did they cut your belly open to take the baby out?	1. YES<br>2. NO
**418A_2.** Did you have a companion or support person accompany you to the facility where you delivered?	1. YES<br>2. NO → **Q418F_2**<br>8. DOESN'T KNOW / REMEMBER → **Q418F_2**

**418B_2.** Was your companion or support person allowed to:		**YES**	**NO**
A. Be with you during labor?	A. DURING LABOR	1	2
B. Be with you during delivery?	B. DURING DELIVERY	1	2
C. Be with you after delivery?	C. AFTER DELIVERY	1	2

**418C_2.** Who was your companion or support person?<br>PROBE: Anyone else?<br>**RECORD ALL MENTIONED.**	A. HUSBAND OR PARTNER<br>B. MOTHER<br>C. SISTER<br>D. MOTHER-IN-LAW<br>E. OTHER RELATIVE<br>F. TRADITIONAL BIRTH ATTENDANT<br>G. NEIGHBOR<br>H. FRIEND<br>I. OTHER (SPECIFY) ___________________

**418F_2.**	**INTERVIEWER: DID THE DELIVER OCCUR IN A FACILITY?**<br>**CHECK Q416A_2 = 1, 2, 3, OR 4.**	**1. YES → Q419B_2**<br>**2. NO → CONTINUE**
**419A_2.**	Why did you decide to not deliver in a health facility?<br><br>PROBE: Any other reason?<br><br>**RECORD ALL MENTIONED**	A. COST TOO MUCH TO DELIVER AT FACILITY<br>B. FACILITY NOT OPEN<br>C. FACILITY FAR AWAY<br>D. TRANSPORTATION UNAVAILABLE<br>E. TRANSPORTATION EXPENSIVE<br>F. DON'T TRUST FACILITY<br>G. EXPECT POOR QUALITY SERVICE/CARE<br>H. FEAR OF ABUSE/DISRESPECT AT FACILITY<br>I. LACK OF ALTERNATIVE CARE FOR FAMILY<br>J. NO ACCOMODATIONS NEAR FACILITY<br>K. NO FEMALE PROVIDER AT FACILITY<br>L. BABY CAME EARLIER THAN EXPECTED<br>M. HUSBAND/FAMILY DID NOT ALLOW<br>N. NOT NECESSARY<br>O. NOT CUSTOMARY<br>P. OTHER (SPECIFY) ____________________
**419A1_2.**	What was the most important reason?<br>**WRITE THE LETTER FOR THE MOST IMPORTANT REASON MENTIONED IN Q419A_2.**	☐
**419B_2.**	Who made the decision about where to deliver the baby?	1. PREGNANT WOMAN HERSELF<br>2. HUSBAND/PARTNER<br>3. BOTH WOMAN AND HUSBAND/PARTNER<br>4. HUSBAND'S FATHER<br>5. HUSBAND'S MOTHER<br>6. PREGNANT WOMAN'S FATHER<br>7. PREGNANT WOMAN'S MOTHER<br>8. OTHER (SPECIFY) _____________________
**420_2.**	Who assisted with the delivery?<br>PROBE: Anyone else?<br><br>**RECORD ALL MENTIONED. IF RESPONDENT SAYS NO ONE ASSISTED, PROBE TO DETERMINE WHETHER ANY ADULTS WERE PRESENT AT DELIVERY.**	A. DOCTOR / ASSISTANT MEDICAL OFFICER<br>B. CLINICAL OFFICER<br>C. ASSISTANT CLINICAL OFFICER<br>D. TRAINED NURSE / MIDWIFE<br>E. MCH AIDE<br>F. MEDICAL ATTENDANT<br>G. NURSE ASSISTANT<br>H. VILLAGE HEALTH WORKER<br>I. TBA<br>J. RELATIVE/FRIEND<br>K. OTHER (SPECIFY) ____________________<br>L. NO ONE<br>M. DOESN'T KNOW

**421_2.**	During the first 6 weeks after the birth, did you have any of the following complications?<br>**READ A–K. CIRCLE YES OR NO FOR EACH OPTION.**		**YES**	**NO**
	A. Severe Bleeding	A. SEVERE BLEEDING	1	2
	B. Bad-smelling Vaginal Discharge	B. BAD-SMELLING VAGINAL DISCHARGE	1	2
	C. Infection of Surgical Wound	C. INFECTION OF SURGICAL WOUND	1	2
	D. Faint/coma	D. FAINT/COMA	1	2
	E. High Fever (39-40c)	E. HIGH FEVER (39-40C)	1	2
	F. Painful Urination	F. PAINFUL URINATION	1	2
	G. Painful Uterus (pelvic pain)	G. PAINFUL UTERUS (PELVIC PAIN)	1	2
	H. Breast Infection	H. BREAST INFECTION	1	2
	I. Continuous leaking of urine from the vagina	I. CONTINUOUS LEAKING OF URINE FROM VAGINA	1	2
	J. Continuous leaking of faeces from the vagina	J. CONTINUOUS LEAKING OF FAECES FROM VAGINA	1	2
	K. Any other complication?	K. OTHER (SPECIFY) _______________	1	2

**422_2.**	Did you have any seizures or convulsions while pregnant, in labor or immediately after delivery?<br>**PROBE: Were you told that you had eclampsia?**	1. YES<br>2. NO<br>8. DOES NOT REMEMBER

**423A_2. REVIEW 416A_2: IF DELIVERY OCCURRED IN A FACILITY (416A_2=1-4), ASK:** After you left the facility, did any health care provider or a traditional birth attendant check on your health?<br><br>**IF DELIVERY OCCURRED OUTSIDE A FACILITY (416A_2=5-8), ASK:** After the baby was born, did any health care provider or a traditional birth attendant check on your health?	1. YES<br>2. NO **→Q424A_2**<br>8. DOES NOT REMEMBER **→Q424A_2**
**423B_2.** How long after delivery did the first check take place?<br><br>**INTERVIEWER: IF 'SAME DAY', PROBE TO BE SURE THE CHECK OCCURRED DURING A SEPARATE VISIT.**	1. ___ ___ DAYS<br><br>2. ___ ___ WEEKS<br><br>000. SAME DAY<br><br>888. DON'T REMEMBER
**423C_2.** Where did the first check take place?	1. HOSPITAL (SPECIFY) ______________<br>2. HEALTH CENTER (SPECIFY) ______________<br>3. DISPENSARY (SPECIFY) ______________<br>4. HOME<br>5. CBD WORKER<br>6. OTHER (SPECIFY) ______________
**424A_2.** At any time during that pregnancy, including after delivery, were you counseled about family planning?	1. YES<br>2. NO **→ Q425F_2**<br>8. DOES NOT KNOW **→ Q425F_2**

**424B_2.** Did you receive counseling about family planning during…? **READ A-C.**		**YES**	**NO**
A. Antenatal care	A. ANTENATAL CARE	1	2
B. Time of delivery	B. AT THE TIME OF DELIVERY	1	2
C. Postpartum check-up	C. POSTPARTUM CHECK UP	1	2

425F_2. INTERVIEWER: REVIEW Q401_2 AND RECORD THE PREGNANCY OUTCOME OF THE SECOND-TO-LAST BIRTH.

PREGNANCY OUTCOME: ☐

425FF_2. INTERVIEWER: VERIFY 425F_2 AND PREGNANCY HISTORY ON P.5, AND CIRCLE ONE ANSWER:

1. **IF THE PREGNANCY OUTCOME WAS NOT A LIVE BIRTH (CODE 4, 5, OR 3 IF FIRST TWIN WAS BORN DEAD), SKIP TO Q431_2.**
2. **IF THE PREGNANCY OUTCOME IS A LIVE BIRTH (CODE 1), CONTINUE.**
3. **IF THE PREGNANCY OUTCOME IS TWINS (BOTH ALIVE) (CODE 2), CONTINUE.**
4. **IF THE PREGNANCY OUTCOME IS TWINS (1 LIVE BIRTH, 1 STILLBIRTH) (CODE 3), CONTINUE IF THIS FIRST TWIN WAS BORN ALIVE (CHECK PREGNANCY HISTORY ON P. 5).**

**426A_2.** Was (NAME) weighed at birth?	1. YES<br>2. NO **→ Q426C_2**<br>8. DOESN'T KNOW **→ Q426C_2**
**426B_2.** How much did (NAME) weigh?	____ . _____ KILOGRAMS **→ GO TO Q427_2**<br><br>88. DON'T KNOW
**426C_2.** Do you know if (NAME) weighed less than 2.5 kg or was considered too small?	1. YES, WAS LESS THAN 2.5 kg<br>2. NO, WAS MORE THAN 2.5 kg<br>8. DOESN'T KNOW / REMEMBER
**426D_2.** Immediately after the birth, was (NAME) put directly on the bare skin of your chest?	1. YES<br>2. NO<br>8. DOES NOT KNOW
**426E_2.** After you delivered, did the facility give you a birth notification form?	1. YES<br>2. NO<br>8. DOES NOT KNOW
**427_2.** Does (NAME) have a birth certificate?	1. YES<br>2. NO<br>8. DOES NOT KNOW
**428A_2.** Did you ever breastfeed (NAME)?	1. YES<br>2. NO **→ Q429A_2**

**428B_2.**	How long after birth did you first put (NAME) to the breast?	1. ___ ___ HOURS<br>OR<br>2. ___ ___ DAYS<br>000. WITHIN ONE HOUR<br>888. DO NOT REMEMBER
**428C_2.**	**CHECK 320: IS THE CHILD FROM THE SECOND-TO-LAST BIRTH STILL LIVING?**	1. YES<br>2. NO → **Q429A_2**
**428D_2.**	Are you still breastfeeding (NAME)?	1. YES<br>2. NO → **Q428H_2**
**428E_2.**	Now I would like to ask you about liquids or foods (NAME) had yesterday during the day or night. Did (NAME) drink/eat:<br>**READ A–E. CIRCLE YES OR NO FOR EACH.**	**YES** / **NO**
	A. Plain water?	A. PLAIN WATER 1 2
	B. Commercially produced infant formula?	B. FORMULA 1 2
	C. Any [BRAND NAME OF COMMERCIALLY FORTIFIED BABY FOOD, e.g. Cerelac]?	C. BABY CEREAL 1 2
	D. Any milk from animals?	D. ANIMAL MILK 1 2
	E. Any (other) porridge like ugali?	E. PORRIDGE/UGALI 1 2
**428F_2.**	**INTERVIEWER: CHECK Q428E_2. DID THE CHILD FROM THE LAST BIRTH HAVE ANY DRINK OR FOOD OTHER THAN BREAST MILK (ANY "YES" IN Q428E_2)?**	1. YES → **Q429A_2**<br>2. NO
**428G_2.**	Aside from breastmilk, did (NAME) have anything at all to eat or drink yesterday or last night?<br>**RECORD RESPONSE. THEN GO TO Q429A_2.**	1. YES<br>2. NO<br>8. DOES NOT KNOW<br>} **Q429A_2**
**428H_2.**	For how many months did you breastfeed (NAME)?	[ ][ ] MONTHS<br>88. DO NOT REMEMBER
**429A_2.**	In the two months after (NAME) was born, did any health care provider or traditional birth attendant check on the baby's health?	1. YES<br>2. NO → **Q431_2**<br>3. NO, BABY DIED → **Q431_2**<br>8. DOES NOT REMEMBER → **Q431_2**
**429B_2.**	How many days or weeks after (NAME's) birth did the first health check take place?	1. ___ ___ DAYS OR<br>2. ___ ___ WEEKS<br>000. SAME DAY<br>888. DO NOT REMEMBER
**430_2.**	Was the health check because (NAME) was sick or was it a routine health exam?	1. HEALTH CHECK FOR SICKNESS<br>2. ROUTINE HEALTH CHECK<br>8. DOES NOT REMEMBER
**431_2.**	How many months after (NAME's) birth did your period (menstruation) return?	[ ][ ] MONTHS<br>66. NOT YET RESUMED
**432_2.**	How many months after (NAME's) birth did you resume sexual relations?	[ ][ ] MONTHS<br>66. NOT YET RESUMED
**433F_2.**	**INTERVIEWER, WAS THE SECOND-TO-LAST BIRTH A TWIN BIRTH (Q425F_2=2, 3, OR 5)?**	**1. YES**<br>**2. NO → Q400_3**
**433FF_2.**	**INTERVIEWER, WAS THE SECOND TWIN FROM THIS BIRTH BORN ALIVE (Q425F_2=2, OR Q425F_2=3 IF SECOND TWIN WAS BORN ALIVE)?**	**1. BOTH TWINS ALIVE**<br>**2. FIRST TWIN DEAD, SECOND TWIN ALIVE**<br>**3. FIRST TWIN ALIVE, SECOND TWIN DEAD → Q400_3**<br>**4. BOTH TWINS DEAD → Q400_3**
**434A_2.**	Now I would like to talk about the second twin, (NAME). Was (NAME) also weighed at birth?	1. YES<br>2. NO → **Q434C_2**<br>8. DOES NOT KNOW → **Q434C_2**
**434B_2.**	How much did (NAME) weigh?	___ . ___ **KILOGRAMS → Q435_2**<br>88. DON'T KNOW
**434C_2.**	Do you know if (NAME) weighed less than 2.5 kg or was considered too small?	1. YES, WAS LESS THAN 2.5 kg<br>2. NO, WAS MORE THAN OR EQUAL TO 2.5 kg<br>8. DOESN'T KNOW / REMEMBER

**434D_2.** Immediately after the birth, was (NAME) put directly on the bare skin of your chest?	1. YES<br>2. NO<br>8. DOES NOT KNOW
**434E_2.** After you delivered, did the facility give you a birth notification form?	1. YES<br>2. NO<br>8. DOES NOT KNOW
**435_2.** Does (NAME) have a birth certificate?	1. YES<br>2. NO<br>8. DOES NOT KNOW
**436A_2.** Did you ever breastfeed (NAME)?	1. YES<br>2. NO → **Q437A_2**
**436B_2.** How long after birth did you first put (NAME) to the breast?	1. ___ ___ HOURS OR<br>2. ___ ___ DAYS<br>000. WITHIN ONE HOUR<br>888. DO NOT REMEMBER
**436C_2. INTERVIEWER, CHECK Q320: IS THE SECOND TWIN OF THE SECOND-TO-LAST BIRTH STILL LIVING?**	**1. YES**<br>**2. NO → Q437A_2**
**436D_2.** Are you still breastfeeding (NAME)?	1. YES<br>2. NO → Q436H_2
**436E_2.** Now I would like to ask you about liquids or foods (NAME) had yesterday during the day or night. Did (NAME) drink/eat: **READ A–E. CIRCLE YES OR NO FOR EACH.**	YES / NO
A. Plain water?	A. PLAIN WATER 1 2
B. Commercially produced infant formula?	B. FORMULA 1 2
C. Any milk from animals?	C. ANIMAL MILK 1 2
D. Tea, juice, or other liquids?	D. OTHER LIQUIDS 1 2
E. Any [BRAND NAME OF COMMERCIALLY FORTIFIED BABY FOOD, E.G. Cerelac]?	E. BABY CEREAL 1 2
F. Any (other) porridge like ugali?	F. PORRIDGE/UGALI 1 2
**436F_2. INTERVIEWER CHECK Q436E: DID THE CHILD FROM THE LAST BIRTH HAVE ANY DRINK OR FOOD OTHER THAN BREAST MILK (ANY "YES" IN Q436E_2)?**	**1. CHILD HAD ANY OF THE FOOD/DRINK → Q437A_2**<br>**2. CHILD DID NOT HAVE ANY FOOD/DRINK → CONTINUE**
**436G_2.** Aside from breastmilk, did (NAME) have anything at all to eat or drink yesterday or last night?<br>**RECORD RESPONSE. THEN GO TO Q437A_2.**	1. YES → **Q437A_2**<br>2. NO → **Q437A_2**<br>8. DOES NOT KNOW → **Q437A_2**
**436H_2.** For how many months did you breastfeed (NAME)?	[ ][ ] MONTHS<br>88. DO NOT REMEMBER
**437A_2.** In the <u>two months after</u> (NAME) was born, did any health care provider or traditional birth attendant check on his/her health?	1. YES<br>2. NO → **Q400_3**<br>3. NO, BABY DIED → **Q400_3**<br>8. DOES NOT REMEMBER → **Q400_3**
**437B_2.** How many days or weeks after (NAME's) birth did the first health check take place?	1. ___ ___ DAYS OR<br>2. ___ ___ WEEKS<br>000. SAME DAY<br>888. DO NOT REMEMBER
**438_2.** Was the health check because (NAME) was sick or was it a routine health exam?	1. HEALTH CHECK FOR SICKNESS<br>2. ROUTINE HEALTH CHECK<br>8. DOES NOT REMEMBER

SECTION IV: HEALTH CARE FOR BIRTHS SINCE JANUARY 2014
THIRD-TO-LAST BIRTH

**400_3. INTERVIEWER, DID ANY <u>THIRD-TO-LAST BIRTH</u> OCCUR SINCE JANUARY 2014 (CHECK Q400 AND Q315)?**	**1. YES**<br>**2. NO → GO TO SECTION V**<br>**3. ONLY ONE BIRTH → GO TO SECTION V**
**401_3. INTERVIEWER, COPY THE PREGNANCY NUMBER AND OUTCOME OF THE <u>THIRD-TO-LAST</u> SINCE JANUARY 2014 ON PAGE 5.**	**PREGNANCY # FOR THIRD-TO-LAST BIRTH:** ☐<br>**PREGNANCY OUTCOME CODE:**<br>**"1", "2", "3", "4, or "5"** ☐<br>**IF CODE= 1, 2, or 3 FOR THIRD-TO-LAST BIRTH, RECORD:**<br>**NAME:** ____________________<br>**STILL ALIVE?**<br>**(RECORD IF Q320=1 FOR THIRD-TO-LAST BIRTH)**<br>1. YES<br>2. NO
**402_3.** Now, I would like to talk to you about your last birth. <u>Just before you got pregnant</u>, did you want to get pregnant then, did you want to get pregnant later, or did you not want to get pregnant then or any time in the future?	1. WANTED THE PREGNANCY THEN<br>2. WANTED THE PREGNANCY LATER<br>3. DID NOT WANT THE PREGNANCY<br>8. NOT SURE
**405_3.** Were you given any injection to prevent the baby from getting tetanus (i.e., *lock jaw*) during that pregnancy?	1. YES - FOR TETANUS<br>2. YES - DON'T KNOW WHAT FOR<br>3. NO → **Q407_3**
**406_3.** How many injections were given?	☐ INJECTIONS<br>8. NOT SURE
**407_3.** Did you see anyone for antenatal care during that pregnancy?	1. YES → **Q408_3**<br>2. NO
**407A_3.** Why didn't you go for antenatal care?<br>PROBE: Any other reason?<br>**RECORD ALL MENTIONED, THEN GO TO Q414A**	A. COST TOO MUCH<br>B. FACILITY FAR AWAY<br>C. TRANSPORTATION UNAVAILABLE/EXPENSIVE<br>D. DON'T TRUST FACILITY/POOR QUALITY SERVICE<br>E. NO FEMALE PROVIDER AT FACILITY<br>F. HUSBAND/FAMILY DID NOT ALLOW<br>G. NOT NECESSARY<br>H. NOT CUSTOMARY<br>I. OTHER ________________<br>**GO TO Q414A_3**
**408_3.** Where did you go for antenatal care?<br>PROBE: Anywhere else?<br>**RECORD ALL MENTIONED.**<br><br>**KIGOMA HOSPITAL CODES:**<br>**BAPTIST: 128**<br>**HERI: 36**<br>**KABANGA: 48**<br>**KASULU (MUMANI): 41**<br>**KIBONDO: 96**<br>**MAWENI: 15**<br><br>**KIGOMA HEALTH CENTER CODES:**<br>**BIHARU: 32**<br>**BITALE: 3**<br>**BUHINGU: 81**<br>**GUNGU (KIKUKU): 10**<br>**GWANUMPU: 86**<br>**ILAGALA: 12**<br>**JANDA: 39**<br>**KAKONKO: 88**<br>**KIFURA: 99**<br>**KIGANAMO: 46**<br>**KIMWANYA: 129**<br>**MABAMBA: 110**<br>**MATYAZO: 14**<br>**MULERA: 60**<br>**MUYAMA: 63**<br>**MUYOVOZI: 130**<br>**MWAMGONGO: 30**<br>**NGURUKA: 21**<br>**NYWAKITONTO: 70**<br>**NYANZIGE: 118**<br>**NYENGE: 5**<br>**RUSESA: 76**<br>**SHUNGA: 77**<br>**UJIJI: 24**<br>**UVINZA: 27**	A. KIGOMA HOSPITAL: CODE ____ ____ ____<br>B. OTHER HOSPITAL (SPECIFY) ________________<br>C. KIGOMA HEALTH CENTER: CODE ___ ___ ___<br>D. DISPENSARY ____________________________<br>E. HOME<br>F. CBD WORKER<br>G. COMMUNITY HEALTH WORKER (CHW)<br>H. TRAINED BIRTH ATTENDANT<br>I. OTHER (SPECIFY) ____________________________
**409_3.** How many times did you go?	☐☐ TIMES<br>66. NOT SURE, BUT AT LEAST 4 TIMES<br>77. NOT SURE<br>88. DOES NOT REMEMBER

**410_3.** In what month of the pregnancy did the antenatal care begin?	[ ][ ] MONTH<br>88. DOES NOT REMEMBER
**411_3.** During this pregnancy, were any of the following done at least once:<br>**READ A-E. CIRCLE YES OR NO FOR EACH.**<br>A. Were you weighed?<br>B. Was your height measured?<br>C. Did you give a urine sample?<br>D. Did you give a blood sample?<br>E. Were you tested for HIV?	**YES** **NO**<br>A. WEIGHT 1 2<br>B. HEIGHT 1 2<br>C. URINE SAMPLE 1 2<br>D. BLOOD SAMPLE 1 2<br>E. HIV TESTED 1 2
**412A_3.** Was your blood pressure ever checked during that pregnancy?	1. YES<br>2. NO → **Q413A_3**<br>8. DOES NOT REMEMBER → **Q413A_3**
**412B_3.** During this pregnancy, were you told that your blood pressure was high?	1. YES<br>2. NO<br>8. DOES NOT REMEMBER
**413A_3.** Were you told about the signs of pregnancy complications?	1. YES<br>2. NO<br>8. DOES NOT REMEMBER
**413B_3.** Were you advised to develop a birth plan?	1. YES<br>2. NO<br>8. DOES NOT REMEMBER
**413B2_3.** What arrangements did you or your family make for the birth of this child?<br>**READ A-E. CIRCLE YES OR NO FOR EACH.**<br>Did you:<br>A. Identify transport?<br>B. Save money?<br>C. Identify a blood donor?<br>D. Identify a nurse, midwife, or doctor to deliver the baby?<br>E. Identify a companion or support person to accompany you to a facility for delivery?	**YES** **NO**<br>A. TRANSPORT 1 2<br>B. SAVE MONEY 1 2<br>C. BLOOD DONOR 1 2<br>D. SKILLED ATTENDANT 1 2<br>E. COMPANION / SUPPORT FACILITY 1 2
**414A_3.** Did you sleep under insecticide-treated bed nets (ITN) most of the time?	1. YES<br>2. NO<br>8. DOES NOT REMEMBER
**414B_3.** During this pregnancy, did you take SP-Fansidar to keep you from getting malaria?	1. YES<br>2. NO → **Q414D_3**<br>8. DOES NOT REMEMBER → **Q414D_3**
**414C_3.** How many times did you take SP-Fansidar?	[ ][ ] TIMES
**414D_3.** Did you take any local herbs during your pregnancy and/or labor?	1. YES<br>2. NO → **Q415_3**<br>8. DOES NOT REMEMBER → **Q415_3**
**414E_3.** For what reasons did you take the local herbs?<br>PROBE: Any other reason?<br>RECORD ALL MENTIONED	A. TO INDUCE OR SUSTAIN LABOR (CONTRACTION THAT LED UP TO BIRTH)<br>B. TO TREAT MALARIA<br>C. TO TREAT COLD/FLU<br>D. TO TREAT HEADACHE<br>E. TO TREAT CONVULSIONS<br>F. TO TREAT VAGINAL BLEEDING<br>G. TO TREAT STOMACH PAIN<br>H. FOR THE HEALTH OF THE CHILD<br>I. TO AVOID MISCARRIAGE<br>J. OTHER (SPECIFY) ________________________
**414F_3.** Altogether, how many days did you take local herbs during the pregnancy and/or labor?<br>IF ANSWER IS GIVEN IN MONTHS, CONVERT IT TO DAYS.	___ ___ ___ DAYS<br>777. CONTINUOUSLY<br>888. DOES NOT REMEMBER
**414G_3.** In what month of the pregnancy did you begin using local herbs?	___ ___ MONTH<br>77. JUST BEFORE DELIVERY<br>88. DOES NOT REMEMBER

**414H_3.** In what month of the pregnancy did you stop using local herbs?	___ ___ MONTH<br><br>55. DURING LABOR / JUST BEFORE DELIVERY<br>66. DURING DELIVERY<br>77. AFTER DELIVERY<br>88. DOES NOT REMEMBER
**415_3.** Before you delivered the baby, how long had you been in labor? By labor, we mean strong, regular and frequent contractions at least 5 minutes apart.	___ ___ HOURS<br>76. MORE THAN 3 DAYS (>72 HOURS)<br>77. C-SECTION BEFORE LABOR<br>88. DOESN'T KNOW
**416A_3.** Where did you give birth to this baby?<br><br>**KIGOMA HOSPITAL CODES:**<br>**BAPTIST: 128**<br>**HERI: 36**<br>**KABANGA: 48**<br>**KASULU (MUMANI): 41**<br>**KIBONDO: 96**<br>**MAWENI: 15**<br><br>**KIGOMA HEALTH CENTER CODES:**<br>**BIHARU: 32**<br>**BITALE: 3**<br>**BUHINGU: 81**<br>**GUNGU (KIKUKU): 10**<br>**GWANUMPU: 86**<br>**ILAGALA: 12**<br>**JANDA: 39**<br>**KAKONKO: 88**<br>**KIFURA: 99**<br>**KIGANAMO: 46**<br>**KIMWANYA: 129**<br>**MABAMBA: 110**<br>**MATYAZO: 14**<br>**MULERA: 60**<br>**MUYAMA: 63**<br>**MUYOVOZI: 130**<br>**MWAMGONGO: 30**<br>**NGURUKA: 21**<br>**NYWAKITONTO: 70**<br>**NYANZIGE: 118**<br>**NYENGE: 5**<br>**RUSESA: 76**<br>**SHUNGA: 77**<br>**UJIJI: 24**<br>**UVINZA: 27**	1. KIGOMA HOSPITAL: CODE ___ ___ ___<br>2. OTHER HOSPITAL (SPECIFY) ______________<br>3. KIGOMA HEALTH CENTER: CODE ___ ___ ___<br>4. DISPENSARY (SPECIFY) _________________<br>5. ON THE WAY TO A HEALTH FACILITY → **Q419B_3**<br>6. OWN HOME → **Q419A_3**<br>7. OTHER HOME → **Q419A_3**<br>8. OTHER (SPECIFY) _________________ → **Q419A_3**
**416A1_3.** Would you recommend this health facility to a friend or family member for maternal health services?	1. YES<br>2. NO<br>8. DON'T KNOW
**416A2_3.** Was this the closest health facility to your home?	1. YES → **Q416B_3**<br>2. NO<br>8. DON'T KNOW → **Q416B_3**
**416A3_3.** What are the reasons you did not go to the facility nearest to your home?<br><br>PROBE: "Anything else?"<br><br>**RECORD ALL MENTIONED**	A. INCONVENIENT OPERATING HOURS<br>B. BAD REPUTATION<br>C. DON'T LIKE STAFF<br>D. NO MEDICINE<br>E. PREFERS TO REMAIN ANONYMOUS (NO ONE KNOWS ME AT THIS FACILITY)<br>F. IT IS MORE EXPENSIVE<br>G. REFERRED FROM ANOTHER FACILITY<br>H. FACILITY NOT OPEN<br>I. FACILITY OF POOR QUALITY<br>J. FACILITY DOES NOT PROVIDE DESIRED SERVICES<br>K. PROVIDERS OFTEN AWAY<br>L. FACILITY DOES NOT ACCEPT INSURANCE<br>M. PROVIDER TREATS PATIENTS UNKINDLY<br>N. BAD EXPERIENCE AT THE FACILITY IN THE PAST<br>O. OTHER (SPECIFY) ________________________<br>Y. DON'T KNOW / REMEMBER
**416B_3.** Were you referred for the third-to-last-birth?	1. YES<br>2. NO
**417A_3.** How long did it take you, one-way, to get from your home to the health facility where you delivered?	1. ___ ___ MINUTES<br><br>OR<br><br>2. ___ ___ HOURS<br><br>888. DON'T REMEMBER
**417B_3.** How did you get to the health facility?<br><br>PROBE: Any other way?<br><br>**RECORD ALL MENTIONED**	A. WALKED / ON FOOT<br>B. AMBULANCE BICYCLE<br>C. AMBULANCE MOTORCYCLE<br>D. AMBULANCE CAR / TRUCK<br>E. SIMPLE BICYCLE<br>F. SIMPLE MOTORCYCLE<br>G. SIMPLE CAR / TRUCK<br>H. BOAT<br>I. BUS / MINIBUS<br>J. BAJAJ<br>K. CART<br>L. LITTER (HOMEMADE GURNEY)<br>M. OTHER (SPECIFY) _______________

**417C_3.** How much did you pay for transport to the facility, in total?	___ ___ ___ ___ ___ ___ TSH<br>000000. NONE<br>777777. PAID IN-KIND<br>888888. DOESN'T KNOW
**417D_3.** Did you pay any formal or informal fees for deliver care for your third-to-last birth?	1. YES<br>2. NO → **Q417G_3**
**417E_3.** How much did you pay in <u>formal fees</u> for delivery care services at the facility?	___ ___ ___ ___ ___ ___ TSH<br>000000. NONE<br>888888. DOESN'T KNOW
**417F_3.** How much did you pay in <u>informal fees</u> for delivery care services at the facility?	___ ___ ___ ___ ___ ___ TSH<br>000000. NONE<br>888888. DOESN'T KNOW
**417G_3.** Did the facility provide supplies for childbirth?	1. YES<br>2. NO → **Q417I_3**
**417H_3.** How much did you pay for supplies at the facility?	___ ___ ___ ___ ___ ___ TSH<br>000000. NONE<br>777777. PAID IN-KIND<br>888888. DOESN'T KNOW
**417I_3.** How much did you pay for any supplies that you brought?	___ ___ ___ ___ ___ ___ TSH<br>000000. NONE<br>777777. PAID IN-KIND<br>888888. DOESN'T KNOW
**417J_3.** How much did you pay for any medications?	___ ___ ___ ___ ___ ___ TSH<br>000000. NONE<br>777777. PAID IN-KIND<br>888888. DOESN'T KNOW
**417K_3.** How much did you pay for accommodation near the facility?	___ ___ ___ ___ ___ ___ TSH<br>000000. NONE<br>777777. PAID IN-KIND<br>888888. DOESN'T KNOW
**417L_3.** How much did you pay for food at the facility?	___ ___ ___ ___ ___ ___ TSH<br>000000. NONE<br>777777. PAID IN-KIND<br>888888. DOESN'T KNOW
**417M_3.** How much did you pay for care for your dependents back home?	___ ___ ___ ___ ___ ___ TSH<br>000000. NONE<br>777777. PAID IN-KIND<br>888888. DOESN'T KNOW
**417N_3.** How much did you pay for any other expenses related to your last delivery? (SPECIFY OTHER EXPENSES)<br>____	___ ___ ___ ___ ___ ___ TSH<br>000000. NONE<br>777777. PAID IN-KIND<br>888888. DOESN'T KNOW
**417N2_3.** Did you give gifts or any non-monetary payments for services during your delivery?	1. YES<br>2. NO<br>8. DOESN'T KNOW / REMEMBER
**417O_3.** Thinking back to the <u>way staff treated you</u> during your third-to-last delivery, would you say you were satisfied, somewhat satisfied, or not satisfied?	1. SATISFIED<br>2. SOMEWHAT SATISFIED<br>3. NOT SATISFIED<br>8. DOESN'T KNOW / REMEMBER

417P_3. When you came to the facility for delivery, did the staff….

READ A-L. CIRCLE YES OR NO FOR EACH.

Question		YES	NO
A. Care for you with a kind approach?	A. KIND APPROACH	1	2
B. Treat you in a friendly manner?	B. FRIENDLY MANNER	1	2
C. Show concern and empathy?	C. CONCERN/EMPATHY	1	2
D. Treat you with respect as an individual?	D. TREAT WITH RESPECT	1	2
E. Call you by my name?	E. CALL BY NAME	1	2
F. Respond to your needs whether or not you asked?	F. RESPOND TO NEEDS	1	2
G. Slap you during delivery for different reasons?	G. SLAP DURING DELIV.	1	2
H. Shout at you because you had not done what you were told?	H. SHOUT	1	2
I. Keep you waiting for a long time before receiving service?	I. WAITING LONG TIME	1	2
J. Allow you to practice cultural rituals in the facility?	J. ALLOW RITUALS	1	2
K. Delay service provision due to health facility's internal problems?	K. DELAY SERVICE	1	2
L. Not treat you well because of your personal attributes?	L. NOT TREAT WELL	1	2

**418_3.** Was the baby delivered by caesarean section; that is, did they cut your belly open to take the baby out?	1. YES<br>2. NO
**418A_3.** Did you have a companion or support person accompany you to the facility where you delivered?	1. YES<br>2. NO → **Q418F_3**<br>8. DOESN'T KNOW / REMEMBER → **Q418F_3**
**418B_3.** Was your companion or support person allowed to:<br>A. Be with you during labor?<br>B. Be with you during delivery?<br>C. Be with you after delivery?	<u>**YES**</u> <u>**NO**</u><br>A. DURING LABOR 1 2<br>B. DURING DELIVERY 1 2<br>C. AFTER DELIVERY 1 2
**418C_3.** Who was your companion or support person?<br>PROBE: Anyone else?<br>**RECORD ALL MENTIONED.**	A. HUSBAND OR PARTNER<br>B. MOTHER<br>C. SISTER<br>D. MOTHER-IN-LAW<br>E. OTHER RELATIVE<br>F. TRADITIONAL BIRTH ATTENDANT<br>G. NEIGHBOR<br>H. FRIEND<br>I. OTHER (SPECIFY) ____________________________
**418F_3. INTERVIEWER: DID THE DELIVER OCCUR IN A FACILITY? CHECK Q416A_3 = 1, 2, 3, OR 4.**	**1. YES → Q419B_3**<br>**2. NO → CONTINUE**
**419A_3.** Why did you decide to not deliver in a health facility?<br>PROBE: Any other reason?<br>**RECORD ALL MENTIONED**	A. COST TOO MUCH TO DELIVER AT FACILITY<br>B. FACILITY NOT OPEN<br>C. FACILITY FAR AWAY<br>D. TRANSPORTATION UNAVAILABLE<br>E. TRANSPORTATION EXPENSIVE<br>F. DON'T TRUST FACILITY<br>G. EXPECT POOR QUALITY SERVICE/CARE<br>H. FEAR OF ABUSE/DISRESPECT AT FACILITY<br>I. LACK OF ALTERNATIVE CARE FOR FAMILY<br>J. NO ACCOMODATIONS NEAR FACILITY<br>K. NO FEMALE PROVIDER AT FACILITY<br>L. BABY CAME EARLIER THAN EXPECTED<br>M. HUSBAND/FAMILY DID NOT ALLOW<br>N. NOT NECESSARY<br>O. NOT CUSTOMARY<br>P. OTHER (SPECIFY) ____________________________
**419A1_3.** What was the <u>most important</u> reason?<br>**WRITE THE LETTER FOR THE MOST IMPORTANT REASON MENTIONED IN Q419A_3.**	☐
**419B_3.** Who made the decision about where to deliver the baby?	1. PREGNANT WOMAN HERSELF<br>2. HUSBAND/PARTNER<br>3. BOTH WOMAN AND HUSBAND/PARTNER<br>4. HUSBAND'S FATHER<br>5. HUSBAND'S MOTHER<br>6. PREGNANT WOMAN'S FATHER<br>7. PREGNANT WOMAN'S MOTHER<br>8. OTHER (SPECIFY) ____________________________
**420_3.** Who assisted with the delivery?<br>PROBE: Anyone else?<br>**RECORD ALL MENTIONED. IF RESPONDENT SAYS NO ONE ASSISTED, PROBE TO DETERMINE WHETHER ANY ADULTS WERE PRESENT AT DELIVERY.**	A. DOCTOR / ASSISTANT MEDICAL OFFICER<br>B. CLINICAL OFFICER<br>C. ASSISTANT CLINICAL OFFICER<br>D. TRAINED NURSE / MIDWIFE<br>E. MCH AIDE<br>F. MEDICAL ATTENDANT<br>G. NURSE ASSISTANT<br>H. VILLAGE HEALTH WORKER<br>I. TBA<br>J. RELATIVE/FRIEND<br>K. OTHER (SPECIFY) ____________________________<br>L. NO ONE<br>M. DOESN'T KNOW

**421_3.** During the <u>first 6 weeks</u> after the birth, did you have any of the following complications?<br>**READ A–K. CIRCLE YES OR NO FOR EACH OPTION.**	**YES** / **NO**
A. Severe Bleeding	A. SEVERE BLEEDING 1 2
B. Bad-smelling Vaginal Discharge	B. BAD-SMELLING VAGINAL DISCHARGE 1 2
C. Infection of Surgical Wound	C. INFECTION OF SURGICAL WOUND 1 2
D. Faint/coma	D. FAINT/COMA 1 2
E. High Fever (39-40c)	E. HIGH FEVER (39-40C) 1 2
F. Painful Urination	F. PAINFUL URINATION 1 2
G. Painful Uterus (pelvic pain)	G. PAINFUL UTERUS (PELVIC PAIN) 1 2
H. Breast Infection	H. BREAST INFECTION 1 2
I. Continuous leaking of urine from the vagina	I. CONTINUOUS LEAKING OF URINE FROM VAGINA 1 2
J. Continuous leaking of faeces from the vagina	J. CONTINUOUS LEAKING OF FAECES FROM VAGINA 1 2
K. Any other complication?	K. OTHER (SPECIFY) _______________ 1 2
**422_3.** Did you have any seizures or convulsions while pregnant, in labor or immediately after delivery?<br>**PROBE: Were you told that you had eclampsia?**	1. YES<br>2. NO<br>8. DOES NOT REMEMBER
**423A_3. REVIEW 416A_3: IF DELIVERY OCCURRED IN A FACILITY (416A_3=1-4), ASK:** After you left the facility, did any health care provider or a traditional birth attendant check on <u>your</u> health?<br>**IF DELIVERY OCCURRED OUTSIDE A FACILITY (416A_3=5-8), ASK:** After the baby was born, did any health care provider or a traditional birth attendant check on <u>your</u> health?	1. YES<br>2. NO →**Q424A_3**<br>8. DOES NOT REMEMBER →**Q424A_3**
**423B_3.** How long after delivery did the <u>first check</u> take place?<br>**INTERVIEWER: IF 'SAME DAY', PROBE TO BE SURE THE CHECK OCCURRED DURING A SEPARATE VISIT.**	1. ___ ___ DAYS<br>2. ___ ___ WEEKS<br>000. SAME DAY<br>888. DON'T REMEMBER
**423C_3.** Where did the first check take place?	1. HOSPITAL (SPECIFY) ________________________<br>2. HEALTH CENTER (SPECIFY) ________________<br>3. DISPENSARY (SPECIFY) ______________________<br>4. HOME<br>5. CBD WORKER<br>6. OTHER (SPECIFY) ____________________________
**424A_3.** At any time during that pregnancy, including after delivery, were you counseled about family planning?	1. YES<br>2. NO → **Q425F_3**<br>8. DOES NOT KNOW → **Q425F_3**
**424B_3.** Did you receive counseling about family planning during…?<br>**READ A-C.**	**YES** / **NO**
A. Antenatal care	A. ANTENATAL CARE 1 2
B. Time of delivery	B. AT THE TIME OF DELIVERY 1 2
C. Postpartum check-up	C. POSTPARTUM CHECK UP 1 2

425F_3. INTERVIEWER: REVIEW Q401_3 AND RECORD THE PREGNANCY OUTCOME OF THE THIRD-TO-LAST BIRTH.
PREGNANCY OUTCOME: [ ]

425FF_3. INTERVIEWER: VERIFY 425F_3 AND PREGNANCY HISTORY ON P.5, AND CIRCLE ONE ANSWER:

1. **IF THE PREGNANCY OUTCOME WAS NOT A LIVE BIRTH (CODE 4, 5, OR 3 IF <u>FIRST TWIN</u> WAS BORN DEAD), SKIP TO Q431_3.**
2. **IF THE PREGNANCY OUTCOME IS A LIVE BIRTH (CODE 1), CONTINUE.**
3. **IF THE PREGNANCY OUTCOME IS TWINS (BOTH ALIVE) (CODE 2), CONTINUE.**
4. **IF THE PREGNANCY OUTCOME IS TWINS (1 LIVE BIRTH, 1 STILLBIRTH) (CODE 3), CONTINUE IF THIS <u>FIRST TWIN</u> WAS BORN ALIVE (CHECK PREGNANCY HISTORY ON P. 5).**

**426A_3.** Was (NAME) weighed at birth?	1. YES<br>2. NO → **Q426C_3**<br>8. DOESN'T KNOW → **Q426C_3**
**426B_3.** How much did (NAME) weigh?	____ . _____ KILOGRAMS → **GO TO Q427_3**<br>88. DON'T KNOW
**426C_3.** Do you know if (NAME) weighed less than 2.5 kg or was considered too small?	1. YES, WAS LESS THAN 2.5 kg<br>2. NO, WAS MORE THAN 2.5 kg<br>8. DOESN'T KNOW / REMEMBER

**426D_3.** Immediately after the birth, was (NAME) put directly on the bare skin of your chest?	1. YES<br>2. NO<br>8. DOES NOT KNOW
**426E_3.** After you delivered, did the facility give you a birth notification form?	1. YES<br>2. NO<br>8. DOES NOT KNOW
**427_3.** Does (NAME) have a birth certificate?	1. YES<br>2. NO<br>8. DOES NOT KNOW
**428A_3.** Did you ever breastfeed (NAME)?	1. YES<br>2. NO **→ Q429A_3**
**428B_3.** How long after birth did you first put (NAME) to the breast?	1. ___ ___ HOURS<br><br>OR<br>2. ___ ___ DAYS<br>000. WITHIN ONE HOUR<br>888. DO NOT REMEMBER
**428C_3. CHECK 320: IS THE <u>CHILD FROM THE THIRD-TO-LAST BIRTH</u> STILL LIVING?**	**1. YES**<br>**2. NO → Q429A_3**
**428D_3.** Are you still breastfeeding (NAME)?	1. YES<br>2. NO **→ Q428H_3**
**428E_3.** Now I would like to ask you about liquids or foods (NAME) had <u>yesterday</u> during the day or night. Did (NAME) drink/eat:<br>**READ A–E. CIRCLE YES OR NO FOR EACH.**	<u>**YES**</u> <u>**NO**</u>
A. Plain water?	A. PLAIN WATER 1 2
B. Commercially produced infant formula?	B. FORMULA 1 2
C. Any [BRAND NAME OF COMMERCIALLY FORTIFIED BABY FOOD, e.g. Cerelac]?	C. BABY CEREAL 1 2
D. Any milk from animals?	D. ANIMAL MILK 1 2
E. Any (other) porridge like ugali?	E. PORRIDGE/UGALI 1 2
**428F_3. INTERVIEWER: CHECK Q428E_3. DID THE CHILD FROM THE LAST BIRTH HAVE ANY DRINK OR FOOD OTHER THAN BREAST MILK (ANY "YES" IN Q428E_3)?**	**1. YES → Q429A_3**<br>**2. NO**
**428G_3.** Aside from breastmilk, did (NAME) have anything at all to eat or drink yesterday or last night?<br>**RECORD RESPONSE. THEN GO TO Q429A_3.**	1. YES<br>2. NO<br>8. DOES NOT KNOW } **Q429A_3**
**428H_3.** For how many months did you breastfeed (NAME)?	[ ][ ] MONTHS<br><br>88. DO NOT REMEMBER
**429A_3.** In the two months after (NAME) was born, did any health care provider or traditional birth attendant check on <u>the baby's</u> health?	1. YES<br>2. NO **→ Q431_3**<br>3. NO, BABY DIED **→ Q431_3**<br>8. DOES NOT REMEMBER **→ Q431_3**
**429B_3.** How many days or weeks after (NAME's) birth did the first health check take place?	1. ___ ___ DAYS OR<br>2. ___ ___ WEEKS<br>000. SAME DAY<br>888. DO NOT REMEMBER
**430_3.** Was the health check because (NAME) was sick or was it a routine health exam?	1. HEALTH CHECK FOR SICKNESS<br>2. ROUTINE HEALTH CHECK<br>8. DOES NOT REMEMBER
**431_3.** How many months after (NAME's) birth did your period (menstruation) return?	[ ][ ] MONTHS<br><br>66. NOT YET RESUMED
**432_3.** How many months after (NAME's) birth did you resume sexual relations?	[ ][ ] MONTHS<br><br>66. NOT YET RESUMED
**433F_3. INTERVIEWER, WAS THE THIRD-TO-LAST BIRTH A TWIN BIRTH (Q425F_3=2, 3, OR 5)?**	**1. YES**<br>**2. NO → SECTION V**
**433FF_3. INTERVIEWER, WAS THE <u>SECOND TWIN</u> FROM THIS BIRTH BORN ALIVE (Q425F_3=2, OR Q425F_3=3 IF SECOND TWIN WAS BORN ALIVE)?**	**1. BOTH TWINS ALIVE**<br>**2. FIRST TWIN DEAD, SECOND TWIN ALIVE**<br>**3. FIRST TWIN ALIVE, SECOND TWIN DEAD → SECTION V**<br>**4. BOTH TWINS DEAD → SECTION V**

**434A_3.** Now I would like to talk about the second twin, (NAME). Was (NAME) also weighed at birth?	1. YES<br>2. NO → **Q434C_3**<br>8. DOES NOT KNOW → **Q434C_3**
**434B_3.** How much did (NAME) weigh?	___ . ___ KILOGRAMS → **Q435_3**<br><br>88. DON'T KNOW
**434C_3.** Do you know if (NAME) weighed less than 2.5 kg or was considered too small?	1. YES, WAS LESS THAN 2.5 kg<br>2. NO, WAS MORE THAN OR EQUAL TO 2.5 kg<br>8. DOESN'T KNOW / REMEMBER
**434D_3.** Immediately after the birth, was (NAME) put directly on the bare skin of your chest?	1. YES<br>2. NO<br>8. DOES NOT KNOW
**434E_3.** After you delivered, did the facility give you a birth notification form?	1. YES<br>2. NO<br>8. DOES NOT KNOW
**435_3.** Does (NAME) have a birth certificate?	1. YES<br>2. NO<br>8. DOES NOT KNOW
**436A_3.** Did you ever breastfeed (NAME)?	1. YES<br>2. NO → **Q437A_3**
**436B_3.** How long after birth did you first put (NAME) to the breast?	1. ___ ___ HOURS OR<br><br>2. ___ ___ DAYS<br><br>000. WITHIN ONE HOUR<br>888. DO NOT REMEMBER
**436C_3. INTERVIEWER, CHECK Q320: IS THE SECOND TWIN OF THE THIRD-TO-LAST BIRTH STILL LIVING?**	**1. YES**<br>**2. NO → Q437A_3**
**436D_3.** Are you still breastfeeding (NAME)?	1. YES<br>2. NO → **Q436H_3**

		YES	NO
**436E_3.** Now I would like to ask you about liquids or foods (NAME) had yesterday during the day or night. Did (NAME) drink/eat: **READ A–E. CIRCLE YES OR NO FOR EACH.**			
A. Plain water?	A. PLAIN WATER	1	2
B. Commercially produced infant formula?	B. FORMULA	1	2
C. Any milk from animals?	C. ANIMAL MILK	1	2
D. Tea, juice, or other liquids?	D. OTHER LIQUIDS	1	2
E. Any [BRAND NAME OF COMMERCIALLY FORTIFIED BABY FOOD, E.G. Cerelac]?	E. BABY CEREAL	1	2
F. Any (other) porridge like ugali?	F. PORRIDGE/UGALI	1	2

**436F_3. INTERVIEWER CHECK Q436E: DID THE CHILD FROM THE LAST BIRTH HAVE ANY DRINK OR FOOD OTHER THAN BREAST MILK (ANY "YES" IN Q436E_3)?**	**1. CHILD HAD ANY OF THE FOOD/DRINK → Q437A_3**<br>2. **CHILD DID NOT HAVE ANY FOOD/DRINK → CONTINUE**
**436G_3.** Aside from breastmilk, did (NAME) have anything at all to eat or drink yesterday or last night?<br>**RECORD RESPONSE. THEN GO TO Q437A_3.**	1. YES → **Q437A_3**<br>2. NO → **Q437A_3**<br>8. DOES NOT KNOW → **Q437A_3**
**436H_3.** For how many months did you breastfeed (NAME)?	[ ][ ] MONTHS<br><br>88. DO NOT REMEMBER
**437A_3.** In the two months after (NAME) was born, did any health care provider or traditional birth attendant check on his/her health?	1. YES<br>2. NO → **SECTION V**<br>3. NO, BABY DIED → **SECTION V**<br>8. DOES NOT REMEMBER → **SECTION V**
**437B_3.** How many days or weeks after (NAME's) birth did the first health check take place?	1. ___ ___ DAYS OR<br><br>2. ___ ___ WEEKS<br><br>000. SAME DAY<br>888. DO NOT REMEMBER
**438_3.** Was the health check because (NAME) was sick or was it a routine health exam?	1. HEALTH CHECK FOR SICKNESS<br>2. ROUTINE HEALTH CHECK<br>8. DOES NOT REMEMBER

SECTION V– CONTRACEPTIVE AWARENESS AND USE

Now I am going to ask you some questions about Family Planning; this is about methods that couples use to delay or avoid pregnancy.

**READ THE NAME OF EACH METHOD AND CIRCLE "1" OR "2" IN Q501, AS APPROPRIATE.**<br><br>**ONLY ASK 501A and 502 IF 501=1 (YES)**<br>**IF 501=2 (NO), MOVE TO NEXT METHOD.**	**501.** Have you heard of (**METHOD**)?	**502.** Have you ever used (**METHOD**)?	**502A.** What was your main source of information on this method?<br>**(RECORD CODE FROM LIST BELOW TABLE)**
**METHOD**			
**A. Female sterilization, tubal ligation**<br>PROBE: Women can have an operation to avoid having more children.	1. YES → **Continue to 502**<br>2. NO	1. YES<br>2. NO } **502A**	___ ___
**B. Male sterilization, vasectomy**<br>PROBE: Men can have an operation to avoid having more children.	1. YES → **Continue to 502**<br>2. NO	1. YES<br>2. NO } **502A**	___ ___
**C. IUD**<br>PROBE: Women can have a loop or coil placed inside them by a doctor or nurse.	1. YES → **Continue to 502**<br>2. NO	1. YES<br>2. NO } **502A**	___ ___
**D. Injectables**<br>PROBE: Women can have an injection by a health provider that stops them from becoming pregnant for one or more months.	1. YES → **Continue to 502**<br>2. NO	1. YES<br>2. NO } **502A**	___ ___
**E. Implants** (e.g. Implanon, Nexplanon , Jadelle, Norplant)<br>PROBE: Women can have several small rods placed in their upper arm by a doctor or nurse, which can prevent pregnancy for one or more months.	1. YES → **Continue to 502**<br>2. NO	1. YES<br>2. NO } **502A**	___ ___
**F. Pill**<br>PROBE: Women can take a pill every day to avoid becoming pregnant.	1. YES → **Continue to 502**<br>2. NO	1. YES<br>2. NO } **502A**	___ ___
**G. Male Condom**<br>PROBE: Men can put a rubber sheath on their penis before sexual intercourse.	1. YES → **Continue to 502**<br>2. NO	1. YES<br>2. NO } **502A**	___ ___
**H. Female Condom**<br>PROBE: Women can place a sheath in their vagina before sexual intercourse.	1. YES → **Continue to 502**<br>2. NO	1. YES<br>2. NO } **502A**	___ ___
**I. Diaphragm**<br>PROBE: Women can place a shallow cup in their vagina before sexual intercourse.	1. YES → **Continue to 502**<br>2. NO	1. YES<br>2. NO } **502A**	___ ___
**J. Foam or Jelly**<br>PROBE: Women can place a suppository, jelly or cream in their vagina before sexual intercourse.	1. YES → **Continue to 502**<br>2. NO	1. YES<br>2. NO } **502A**	___ ___
**K. Lactational Amenorrhea Method (LAM)**	1. YES → **Continue to 502**<br>2. NO	1. YES<br>2. NO } **502A**	___ ___
**L. Rhythm**<br>PROBE: Every month that a woman is sexually active she can avoid pregnancy by not having sexual intercourse on the days of the month she is most likely to get pregnant.	1. YES → **Continue to 502**<br>2. NO	1. YES<br>2. NO } **502A**	___ ___
**M. Withdrawal**<br>PROBE: Men can be careful and pull out before climax.	1. YES → **Continue to 502**<br>2. NO	1. YES<br>2. NO } **502A**	___ ___
**N. Emergency Contraception**<br>PROBE: As an emergency measure after unprotected intercourse, women can take special pills at any time within five days to prevent pregnancy.	1. YES → **Continue to 502**<br>2. NO	1. YES<br>2. NO } **502A**	___ ___
**O.** Have you heard of any other ways or methods that women or men can use to avoid pregnancy?<br>(SPECIFY)______________________________	1. YES → **Continue to 502**<br>2. NO → **Q503A**	1. YES<br>2. NO } **502A**	___ ___

CODES FOR Q502A:

1. PARENT(S) / GUARDIAN(S)
2. SISTER(S) / BROTHERS (S)
3. OTHER RELATIVE
4. TEACHER
5. PEERS / FRIENDS
6. CLINIC STAFF
7. COMMUNITY HEALTH WORKER
8. PHARMACIST
9. RELIGIOUS LEADER
10. BOOKS
11. NEWSPAPER, MAGAZINES, BROCHURES, FLYER
12. WAZAZI NIPENDENI
13. RADIO
14. TELEVISION
15. INTERNET
20. OTHER ____________________
88. DON'T REMEMBER

**503A.** In the last 12 months,, were you visited by a community health worker or facility outreach worker who talked to you about family planning?	1. YES, COMMUNITY HEALTH WORKER<br>2. YES, FACILITY OUTREACH WORKER<br>3. YES, BOTH<br>4. NO<br>8. NOT SURE
**503B.** In the last 12 months, have you visited a health facility for care for yourself or your children?	1. YES<br>2. NO → **Q504F**<br>8. NOT SURE → **Q504F**
**503C.** Did any staff member at the health facility speak to you about family planning?	1. YES<br>2. NO<br>8. NOT SURE
**504F. INTERVIEWER, CHECK QUESTION Q502. HAS RESPONDENT EVER USED AT LEAST ONE METHOD OF CONTRACEPTION (AT LEAST ONE "1" CIRCLED IN Q502)?**	**1. EVER USED METHODS (AT LEAST ONE "1" IN Q502_A—Q502_N)**<br>**2. NEVER USED A CONTRACEPTIVE METHOD → Q539**
**505.** How old were you when you first used contraception?	☐☐ YEARS<br>88. DOES NOT REMEMBER
**506.** How many living children did you have when you first used contraception?	☐☐ CHILDREN<br>00. NO LIVING CHILDREN<br>88. DOES NOT REMEMBER
**507.** Are you currently (last 30 days) doing something or using any method to delay or avoid getting pregnant?	1. YES<br>2. NO → **Q530**<br>8. NOT SURE → **Q530**
**508.** Which method are you using?<br><br>**CIRCLE ALL MENTIONED**<br><br>**IF MORE THAN ONE METHOD MENTIONED, FOLLOW SKIP INSTRUCTION FOR HIGHEST METHOD IN LIST.**	A. FEMALE STERILIZATION, TUBAL LIGATION → **Q512**<br>B. MALE STERILIZATION, VASECTOMY → **Q512**<br>C. IUD → **Q514**<br>D. INJECTABLES → **Q509**<br>E. IMPLANT → **Q510**<br>F. PILL → **Q511**<br>G. MALE CONDOM<br>H. FEMALE CONDOM<br>I. DIAPHRAGM<br>J. FOAM/JELLY<br>K. LAM<br>L. RHYTHM<br>M. WITHDRAWAL<br>X. OTHER MODERN METHOD (SPECIFY) ___________<br>Y. OTHER TRADITIONAL METHOD (SPECIFY) ___________<br>(G–X) } **Q514**
**509.** What is the brand name of the injectables you are using?	1. THREE MONTH (DEPO PROVERA)<br>2. OTHER (SPECIFY) ________________<br>8. DOES NOT KNOW / NOT SURE<br>} **Q514**
**510.** What is the brand name of the implant you are using?	1. THREE YEARS (IMPLANON)<br>2. FIVE YEARS (JADELLE)<br>3. OTHER (SPECIFY) ________________<br>8. DOES NOT KNOW<br>} **Q514**
**511.** What is the brand name of the pills you are using?<br><br>**IF DON'T KNOW, ASK TO SEE PACKAGE**	1. MICROGYNON<br>2. LO FEMANAL<br>3. SAFE PLAN<br>4. MACROVAL<br>5. MICROLUT<br>6. FAMILIA<br>7. FLEXIP<br>8. OTHER (SPECIFY) ________________<br>9. DOES NOT KNOW/NOT SURE<br>} **Q514**

**512.**	In what facility did the sterilization take place?	1. HOSPITAL (SPECIFY) ______<br>2. HEALTH CENTER (SPECIFY) ______<br>3. OTHER (SPECIFY) ______<br>8. DOES NOT REMEMBER
**513.**	In what month and year was the sterilization performed?	[ ][ ] MONTH [ ][ ][ ][ ] YEAR<br>88. DNR MONTH 8888. DNR YEAR } **Q533**
**514.**	In what month and year did you start using (**CURRENT METHOD IN Q508**) continuously (without stopping)?<br>**(IF NEEDED, PROBE USING THE PREGNANCY HISTORY: "How long after your last birth did you start using your current method continuously?")**	[ ][ ] MONTH [ ][ ][ ][ ] YEAR<br>88. DNR MONTH 8888. DNR YEAR
**515F.**	**INTERVIEWER: REVIEW Q508.**	**1. HIGHEST RANKED METHOD IS A NATURAL OR TRADITIONAL METHOD (K, L, M, Y) → Q524**<br>**2. HIGHEST RANKED METHOD IS A MODERN METHOD**
**515G.**	Where do you (or your partner) obtain the contraceptive method you are currently using as a couple?<br><br>**(IF MULTIPLE PLACES RECORD <u>THE MAIN SOURCE</u>)**	1. GOVERNMENT HOSPITAL (SPECIFY) ______<br>2. GOVERNMENT HEALTH CENTER (SPECIFY) ______<br>3. GOVERNMENT DISPENSARY(SPECIFY) ______<br>4. RELIGIOUS/PRIVATE HEALTH FACILITY<br>5. PHARMACY<br>6. NGO<br>7. VCT CENTER<br>8. CBD/ VILLAGE HEALTH WORKER<br>9. FAMILY/FRIEND/NEIGHBOR<br>10. MARKET/SHOP/BAR } **Q521A** (4–10)<br>20. OTHER (SPECIFY) ______ → **Q524**<br>88. DOES NOT REMEMBER → **Q524**
**516.**	Who gets the supplies? You, your partner, or both?	1. HERSELF<br>2. HER PARTNER<br>3. BOTH<br>8. DOES NOT REMEMBER
**517.**	Can you get family planning services there at any time or do they only offer family planning services at special times during the day or on certain days?	1. CAN GET FAMILY PLANNING SERVICES AT ANY TIME → **Q521A**<br>2. CAN ONLY GET FAMILY PLANNING SERVICES AT CERTAIN TIMES/DAYS<br>8. DOES NOT KNOW/NOT SURE
**518.**	Are the special times or days convenient for you?	1. YES → **Q521A**<br>2. NO<br>8. NOT SURE

519. What <u>time of day</u> is convenient for you to get family planning services? **(READ A–E).**

Question	Response	**<u>YES</u>**	**<u>NO</u>**
A. Early Morning (Until 10.00AM)	A. EARLY MORNING	1	2
B. Late morning (10.00AM-Noon)	B. LATE MORNING	1	2
C. Early Afternoon (12.00PM-3PM)	C. EARLY AFTERNOON	1	2
D. Late Afternoon (3.00PM-6.00PM)	D. LATE AFTERNOON	1	2
E. Evenings (6.00PM or Later)	E. EVENINGS	1	2

520. Which <u>day, or days of the week</u> are convenient for you? **CIRCLE ALL MENTIONED**

	**<u>MENTIONED</u>**	**<u>NOT MENTIONED</u>**
A. MONDAY	1	2
B. TUESDAY	1	2
C. WEDNESDAY	1	2
D. THURSDAY	1	2
E. FRIDAY	1	2
F. SATURDAY	1	2
G. SUNDAY	1	2

**521A.**	You obtained (**CURRENT METHOD FROM 508**) from (**SOURCE OF METHOD IN 515G**) in (**DATE FROM 514**).<br><br>At that time, were you told about side effects or problems you might have with the method?	1. YES → **Q521C**<br>2. NO<br>8. NOT SURE

**521B.** Were you ever told by a health or family planning worker about side effects or problems you might have with the method?	1. YES<br>2. NO → **Q522A**<br>8. NOT SURE
**521C.** Were you told what to do if you experienced side effects or problems?	1. YES<br>2. NO<br>8. NOT SURE
**522A.** Do you think the waiting time where you get the method is too long or not too long?	1. TOO LONG<br>2. NOT TOO LONG<br>8. DOES NOT KNOW/NOT SURE
**522B.** How long does it take you to get to the place where you or your partner gets the contraceptive supplies?	1. LESS THAN 30 MIN<br>2. 30-59 MINUTES<br>3. 1-2 HOURS<br>4. 2-3 HOURS<br>5. MORE THAN 3 HOURS<br>6. SUPPLIES OFFERED AT HOME OR WORK PLACE<br>7. OTHER (SPECIFY) ___<br>8. DOES NOT KNOW / NOT SURE
**523.** Do you or your partner pay for the contraceptive method you now use?	1. YES<br>2. NO<br>8. NOT SURE
**524.** Would you prefer to use a different method of family planning from the one you are currently using?	1. YES<br>2. NO → **Q533**<br>8. NOT SURE → **Q533**
**525.** What method would you prefer to use?<br><br>(**OTHER THAN THE METHOD SPECIFIED IN Q508)**	1. FEMALE STERILIZATION, TUBAL LIGATION<br>2. MALE STERILIZATION, VASECTOMY<br>3. IUD<br>4. INJECTABLES<br>5. IMPLANT<br>6. PILL<br>7. MALE CONDOM<br>8. FEMALE CONDOM<br>9. DIAPHRAGM<br>10. FOAM/JELLY<br>11. LACTATIONAL AMENHORREA (LAM)<br>12. RHYTHM<br>13. WITHDRAWAL<br>14. OTHER MODERN METHOD (SPECIFY) ___<br>15. OTHER TRADITIONAL METHOD (SPECIFY) ___
**526.** Do you know where to obtain this method? (OR INFORMATION ABOUT A TRADITIONAL METHOD IF IT IS RHYTHM OR WITHDRAWAL.)	1. YES<br>2. NO → **Q529**<br>8. NOT SURE → **Q529**
**527.** Where can you obtain this preferred method?<br><br>(**OR INFORMATION ABOUT A TRADITIONAL METHOD.)**	1. GOVERNMENT HOSPITAL (SPECIFY) ___<br>2. GOVERNMENT HEALTH CENTER (SPECIFY) ___<br>3. GOVERNMENT DISPENSARY (SPECIFY) ___<br>4. RELIGIOUS/PRIVATE HEALTH FACILITY<br>5. PHARMACY<br>6. NGO<br>7. VCT CENTER<br>8. CBD/ VILLAGE HEALTH WORKER<br>9. FRIEND/NEIGHBOR<br>10 MARKET/SHOP/BAR<br>20. OTHER (SPECIFY) ___<br>88. DOES NOT REMEMBER

**528.** How much time would you or your partner have to travel to obtain the supplies or information about the method?	1. LESS THAN 30 MIN<br>2. 30-59 MINUTES<br>3. 1-2 HOURS<br>4. 2-3 HOURS<br>5. MORE THAN 3 HOURS<br>6. SUPPLIES OFFERED AT HOME OR WORK PLACE<br>7. OTHER (SPECIFY)___________________<br>8. DOES NOT KNOW / NOT SURE
**529.** What is the most important reason why you do not use the preferred method?	1. DOCTOR WILL NOT PRESCRIBE IT<br>2. COST<br>3. NOT AVAILABLE/DIFFICULT ACCESS/ UNRELIABLE SOURCE<br>4. SOURCE TOO FAR AWAY<br>5. DOES NOT KNOW HOW/WHERE TO OBTAIN IT<br>6. HUSBAND/PARTNER OBJECTS TO IT<br>7. RELIGIOUS REASONS<br>8. FEAR OF SIDE EFFECTS<br>9. STILL THINKING ABOUT IT/ NOT MADE UP HER MIND<br>10. DIFFICULT TO USE<br>11. FEAR OF SURGERY (IUD, TUBAL LIGATION, IMPLANT)<br>20. OTHER (SPECIFY)_______________<br>88. DOES NOT KNOW<br>(all) → **Q533**
**530.** What was the last contraceptive method you or your partner had used?<br><br>**CIRCLE ONE METHOD ONLY**	1. FEMALE STERILIZATION, TUBAL LIGATION<br>2. MALE STERILIZATION, VASECTOMY<br>3. IUD<br>4. INJECTABLES<br>5. IMPLANT<br>6. PILL<br>7. MALE CONDOM<br>8. FEMALE CONDOM<br>9. DIAPHRAGM<br>10. FOAM/JELLY<br>11. LACTATIONAL AMENHORREA (LAM)<br>12. RHYTHM<br>13. WITHDRAWAL<br>14 OTHER MODERN METHOD (SPECIFY) ___________________<br>15. OTHER TRADITIONAL METHOD (SPECIFY) ________________
**531.** In what month and year did you stop using (**LAST METHOD IN Q530**)? **RECORD LAST MONTH OF USE**	[ ][ ] MONTH [ ][ ][ ][ ] YEAR<br><br>88. DR MONTH 8888. DR YEAR
**532. REVIEW Q530: IF LAM, RHYTHM, OR WITHDRAWAL (Q530=11, 12, 13, OR 15), THEN CIRCLE 11 WITHOUT ASKING.**<br><br>**OTHERWISE, ASK:**<br><br>Where did you obtain that method?	1. GOVERNMENT HOSPITAL (SPECIFY) ______________________<br>2 GOVERNMENT HEALTH CENTER (SPECIFY) _______________<br>3. GOVERNMENT DISPENSARY(SPECIFY) ___________________<br>4. RELIGIOUS/PRIVATE HEALTH FACILITY<br>5. PHARMACY<br>6. NGO<br>7. VCT CENTER<br>8. CBD/ VILLAGE HEALTH WORKER<br>9. FRIEND/NEIGHBOR<br>10. MARKET/SHOP/BAR<br>11. DOES NOT APPLY (NON-SUPPLY METHOD)<br>20. OTHER (SPECIFY) ___________________________________<br>88. DOES NOT REMEMBER
**533.** Now, I would like to ask you some questions on your pregnancies and family planning history during the last few years. First, I will verify with you the pregnancies you may have had since January 2014. **REFER TO THE PREGNANCY HISTORY (Q315-317) AND COMPLETE COLUMN 1 IN THE PREGNANCY AND CONTRACEPTIVE USE CALENDAR ON THE NEXT PAGE.**	
**534F. CHECK Q513, Q514 AND Q531 TO RECORD IF RESPONDENT HAD USED ANY CONTRACEPTIVE METHOD SINCE JANUARY 2014**	**1. HAD USED CONTRACEPTION IN YEAR 2014 OR LATER**<br>**2. HAD NEVER USED CONTRACEPTION OR HAD USED IT BEFORE JAN. 2014 → COMPLETE COLUMN 2 WITH “0”; LEAVE COLUMN 3 BLANK**

535. **COMPLETE THE REST OF THE CONTRACEPTIVE CALENDAR FOR THOSE WHO HAVE USED CONTRACEPTION SINCE JANUARY 2014**
USE CALENDAR TO PROBE FOR PERIODS OF USE AND NONUSE, STARTING WITH EARLIEST USE, BEGINNING WITH JANUARY 2014. USE NAMES OF CHILDREN, DATES OF BIRTH, AND PERIODS OF PREGNANCY AS REFERENCE POINTS.
IN COLUMN 2, ENTER METHOD USE CODE OR ‘0’ FOR NONUSE IN EACH BLANK MONTH. ILLUSTRATIVE QUESTIONS:

- When was the last time you used a method? Which method was that?
- When did you start using that method? How long after the birth of (NAME)?
- How long did you use the method then?

IN COLUMN 3, ENTER CODES FOR DISCONTINUATION ONE MONTH AFTER THE LAST MONTH OF USE. ILLUSTRATIVE QUESTIONS:

- Why did you stop using the (METHOD)?
- Did you become pregnant while using (METHOD), or did you stop to get pregnant, or did you stop for some other reason?

COLUMN 1 (PREGNANCY OUTCOME
0. PREGNANT THAT MONTH
1. LIVE BIRTH
2. MULTIPLE LIVE BIRTHS
3. MULTIPLE (LIVE BIRTH WITH STILLBIRTH)
4. STILLBIRTH (SINGLE)
5. MULTIPLE STILLBIRTH
6. MISCARRIAGE / ABORTION
7. ECTOPIC

COLUMN 2 (METHOD USED)
0. NO METHOD
1. FEMALE STERILIZATION, TUBAL LIGATION
2. MALE STERILIZATION, VASECTOMY
3. IUD
4. INJECTABLES
5. IMPLANTS
6. PILL
7. MALE CONDOM
8. FEMALE CONDOM
9. DIAPHRAGM
10. FOAM/JELLY ________
11. LACTATIONAL AMENORRHEA METHOD
12. RHYTHM METHOD
13. WITHDRAWAL
14. OTHER MODERN________________________
15. OTHER TRADITIONAL
88. DOES NOT REMEMBER

COLUMN 3 (MAIN REASON THE USE WAS STOPPED)
1. GOT PREGNANT WHILE USING
2. WANTED TO GET PREGNANT
3. HUSBAND OBJECTED
4. SIDE EFFECTS
5. HEALTH CONCERNS
6. STOPPED TO “REST THE BODY”
7. PHYSICIAN DECISION
8. SUPPLY/AVAILABILITY
9. DIFFICULT/INCONVENIENT TO USE
10. MARRIAGE/RELATIONSHIP ENDED
11. WANTED TO TRY OTHER METHOD
12. SPORADIC SEXUAL ACTIVITY
13. NOT SEXUALLY ACTIVE
14. NO LONGER ABLE TO GET PREGNANT
15. PERIOD RETURNED (LAM)
16. NEGLIGENCE
20. OTHER (SPECIFY)___________________________
88. DOES NOT KNOW

DATE	1	2	3
**2014**			
1 Jan			
2 Feb			
3 Mar			
4 Apr			
5 May			
6 Jun			
7 Jul			
8 Aug			
9 Sep			
10 Oct			
11 Nov			
12 Dec			
**2015**			
1 Jan			
2 Feb			
3 Mar			
4 Apr			
5 May			
6 Jun			
7 Jul			
8 Aug			
9 Sep			
10 Oct			
11 Nov			
12 Dec			
**2016**			
1 Jan			
2 Feb			
3 Mar			
4 Apr			
5 May			
6 Jun			
7 Jul			
8 Aug			
9 Sep			
10 Oct			
11 Nov			
12 Dec			

536F. INTERVIEWER, CHECK THE CALENDAR AND DETERMINE IF:
1. RESPONDENT USED CONTRACEPTION IN JANUARY 2014 → Q537
2. RESPONDENT DID NOT USE CONTRACEPTION IN JAN. 2014 →Q538F

537. You said that in January of 2014 you were using ___ ___ **(WRITE METHOD CODE USED IN COLUMN 2 IN JAN 2014**). When did you start using that method?

[ ][ ] MONTH [ ][ ][ ][ ] YEAR

88. DNR MONTH 8888. DNR YEAR

**538F.** **INTERVIEWER: VERIFY Q507, Q508, AND COLUMN 2 IN THE CALENDAR FOR THE MONTH OF THE INTERVIEW**	**1. CURRENTLY USING MALE/FEMALE STERILIZATION (A or B) → Q552F**<br>**2. CURRENTLY USING ANOTHER METHOD (C-Y) → Q544**<br>**3. NOT CURRENTLY USING A CONTRACEPTIVE METHOD → CONTINUE**
**539.** Do you think you are physically able to get pregnant at the present time?	1. YES → **Q541**<br>2. CURRENTLY PREGNANT → **Q542**<br>3. NO<br>8. NOT SURE
**540.** What is the main reason why you think you cannot get pregnant?	1. DOES NOT HAVE A PARTNER/NO SEXUAL RELATIONS → **Q542**<br>2. RESPONDENT CURRENTLY IS BREAST-FEEDING /POSTPARTUM → **Q542**<br>3. PELVIC INFLAMMATORY DISEASE (PID)<br>4. HORMONAL DYSFUNCTION<br>5. HYSTERECTOMY (SURGICAL REMOVAL OF UTERUS)<br>6. PREMENOPAUSE/ MENOPAUSE<br>7. OVARIAN CYSTS/ OVARIAN DYSFUNCTION<br>8. RESPONDENT HAD BOTH TUBES REMOVED OR OBSTRUCTED<br>9. SHE DID NOT SUCCEED TO GET PREGNANT IN THE PAST 2 YEARS<br>10. PARTNER IS INFERTILE<br>(3–10) → **Q544**<br>11. CURRENTLY USES A METHOD (**GO BACK TO Q507 AND CORRECT SECTION**)<br>12. OTHER (SPECIFY) ____________________<br>88. DOES NOT KNOW<br>99. REFUSED TO ANSWER
**541.** Why are you not using a method to prevent pregnancy now?<br><br>PROBE: Any other reason?<br><br>**RECORD ALL MENTIONED**	A. SHE WANTS TO GET PREGNANT<br>B. NO SEXUAL RELATIONS<br>C. HEALTH / MEDICAL REASONS<br>D. POSTPARTUM / BREASTFEEDING<br>E. RESPONDENT OPPOSES CONTRACEPTIVES<br>F. PARTNER OPPOSES<br>G. PARTNER WANTS HER TO BECOME PREGNANT<br>H. HEALTH CONCERNS<br>I. FEAR OF SIDE EFFECTS<br>J. SOURCES FAR AWAY<br>K. LACK OF KNOWLEDGE OF METHODS<br>L. LACK OF KNOWLEDGE OF A SOURCE<br>M. LACK OF ACCESS/TOO FAR<br>N. CAN'T AFFORD COST<br>O. HEALTH CARE PROVIDER / PHARMACIST WON'T GIVE THEM<br>P. RELIGION AGAINST<br>Q. FATALISTIC<br>R. OTHER (SPECIFY) _______________<br>Y. NOT SURE
**542.** Do you think you will use a contraceptive method any time in the future?	1. YES<br>2. NO → **Q544**<br>8. NOT SURE → **Q544**
**543.** What method would you want to use most?<br><br>**PROBE FOR THE MOST PREFERRED METHOD.**<br>**CIRCLE ONLY ONE.**	1. FEMALE STERILIZATION, TUBAL LIGATION<br>2. MALE STERILIZATION, VASECTOMY<br>3. IUD<br>4. INJECTABLES<br>5. IMPLANT<br>6. PILL<br>7. MALE CONDOM<br>8. FEMALE CONDOM<br>9. DIAPHRAGM<br>10. FOAM/JELLY<br>11. LAM<br>12. RHYTHM<br>13. WITHDRAWAL<br>14. OTHER MODERN METHOD __________(SPECIFY)<br>15. OTHER TRADITIONAL METHOD:_____________(SPECIFY)<br>88. UNDECIDED

**544.** Now I have some questions about the future. Would you like to have (a/another) child, or would you prefer not to have any (more) children? (**IF CURRENTLY PREGNANT ADD: "after this pregnancy?")**	1. WANT A(ANOTHER) CHILD<br>2. NOT ABLE TO GET PREGNANT → **Q552F**<br>3. NO MORE CHILDREN → **Q547**<br>4. GOD'S WILL, FATE → **Q547**<br>8. NOT SURE → **Q547**
**545. IF NOT PREGNANT, ASK:**<br>How many additional children would you like to have?<br><br>**IF CURRENTLY PREGNANT, ASK:**<br>After this pregnancy, how many more children would you like to have?	[ ][ ] CHILDREN<br><br>66. AS MANY AS POSSIBLE<br>77. GOD'S WILL, FATE<br>88. NOT SURE
**546. IF NOT PREGNANT, ASK:**<br>How long would you like to wait from now before the birth of a/another child?<br><br>**IF CURRENTLY PREGNANT, ASK:**<br><br>How long would you like to wait for the birth of another child, after the birth of the child you are currently expecting?	1. [ ][ ] MONTHS<br><br>2. [ ][ ] YEARS<br><br>000. RIGHT AWAY, AS SOON AS POSSIBLE<br>666. AFTER MARRIAGE<br>777. GOD'S WILL<br>888. NOT SURE
**547.** Do you think you (or your partner) would be interested in having an operation to prevent you from having any more children?<br><br>**IF THE RESPONDENT WANTS TO HAVE MORE CHILDREN (Q544=1) ADD:**<br><br>**"After having all the children you want?"**	1. YES<br>2. NO → **Q551**<br>3. NOT ABLE TO GET PREGNANT → **Q552F**<br>8. NOT SURE
**548.** Do you know where to go for this operation?	1. YES → **Q550**<br>2. NO<br>8. NOT SURE
**549.** Do you know where to get information about this operation?	1. YES<br>2. NO → **Q552F**
**550.** Where? **[IF MORE THAN ONE PLACE MENTIONED, MARK THE ONE SHE WOULD MOST LIKELY USE].**	1. GOVERNMENT HOSPITAL (SPECIFY) ___________<br>2. GOVERNMENT HEALTH CENTER (SPECIFY) ___________<br>3. GOVERNMENT DISPENSARY (SPECIFY)___________<br>4. RELIGIOUS/PRIVATE HEALTH FACILITY<br>5. PHARMACY<br>6. NGO<br>7. CBD/ VILLAGE HEALTH WORKER<br>8. OTHER (SPECIFY) ___________<br>} **Q552F**
**551.** Why would you not be interested in this operation?	1. HEALTH RISKS/FEAR OF SIDE EFFECTS<br>2. FEAR OF OPERATION (SURGERY OR ANESTHESIA)<br>3. THINKING ABOUT IT<br>4. PLANS FOR HER/PARTNER TO HAVE STERILIZATION SOON<br>5. TOO YOUNG<br>6. TOO OLD (APPROACHING MENOPAUSE)<br>7. NOT SEXUALLY ACTIVE/NO PARTNER<br>8. PARNTNER OPPOSES<br>9. MIGHT WANT ANOTHER CHILD<br>10. LACK OF INFORMATION ABOUT THE METHOD OR WHERE TO OBTAIN IT<br>11. MEDICAL FACILITY TOO FAR AWAY<br>12. COST/LACK OF MONEY TO PAY FOR IT<br>13. DOCTOR REFUSED TO DO THE OPERATION/ADVICED AGAINST<br>14. RELIGIOUS REASONS<br>15. PREFERS (OR USES) OTHER CONTRACEPTIVE METHODS<br>20. OTHER (SPECIFY) ___________<br>88. DOES NOT KNOW

**552F. CHECK Q320: DOES THE WOMAN HAVE ANY LIVING CHILDREN?**	**1. YES**<br>**2. NO → Q553B**
**553A.** If you could go back to the time you did not have any children and could choose exactly the number of children to have in your whole life, how many would that be?<br>**PROBE FOR A NUMERIC RESPONSE.**	[ ][ ] CHILDREN<br>00. NONE<br>77. FATE, UP TO GOD<br>88. NOT SURE<br>96. OTHER (SPECIFY) ____________________<br>(all above) → **Q554F**
**553B.** If you could choose exactly the number of children to have in your whole life, how many would that be?<br>**PROBE FOR A NUMERIC RESPONSE.**	[ ][ ] CHILDREN<br>00. NONE<br>77. FATE, UP TO GOD<br>88. NOT SURE<br>96. OTHER (SPECIFY) ____________________
**554F. INTERVIEWER: CHECK Q507, Q508. CIRCLE AN ANSWER ABOUT CURRENT CONTRACEPTIVE USE.**	**1. NOT CURRENTLY USING A CONTRACEPTIVE METHOD**<br>**2. USING FEMALE STERILIZATION OR MALE STERILIZATION (A, B)**<br>**3. USING A TRADITIONAL/NATURAL METHOD (K, L, M, Y)**<br>(1–3) → **SECTION VI**<br>**4. USING OTHER MODERN METHOD → CONTINUE**
**555F. INTERVIEWER: CHECK Q201.**	**1. NOT CURRENTLY IN UNION (Q201=3) → SECTION VI**<br>**2. WOMAN CURRENTLY IN UNION (Q201=1 OR 2) → CONTINUE**
**556.** Does your husband/partner know that you are using a method of family planning?	1. YES<br>2. NO<br>8. NOT SURE
**557.** Would you say that using contraception is mainly your decision, mainly your husband's/partner's decision, or did you both decide together?	1. MAINLY RESPONDENT<br>2. MAINLY HUSBAND/PARTNER<br>3. JOINT DECISION<br>4. OTHER (SPECIFY) __________________

SECTION VI: SEXUAL ACTIVITY AND CHILDBEARING

CHECK FOR THE PRESENCE OF OTHERS. BEFORE CONTINUING, MAKE EVERY EFFORT TO ENSURE PRIVACY.

**600A.**	Now I would like to ask some questions about sexual activity in order to gain a better understanding of some important life issues.<br><br>How long ago did you last have sexual intercourse?	1. ___ ___ DAYS<br>2. ___ ___ WEEKS<br>3. ___ ___ MONTHS<br>4. ___ ___ YEARS<br>000. NEVER HAD SEXUAL INTERCOURSE → **SECTION VII**<br>888. DOES NOT REMEMBER
**600B.**	At the time of your last sexual intercourse, did you or your partner use any contraceptive method?	1. YES<br>2. NO → **Q600D**<br>8. DOES NOT REMEMBER →**Q600D**<br>9. REFUSED → **Q600D**
**600C.**	What was this method?<br><br>**INTERVIEWER:**<br>**VERIFY RESPONSE WITH Q502, 507, 508, AND CALENDAR**	1. FEMALE STERILIZATION, TUBAL LIGATION<br>2. MALE STERILIZATION, VASECTOMY<br>3. IUD<br>4. INJECTABLES<br>5. IMPLANT<br>6. PILL<br>7. MALE CONDOM<br>8. FEMALE CONDOM<br>9. DIAPHRAGM<br>10. FOAM/JELLY<br>11. LAM<br>12. RHYTHM<br>13. WITHDRAWAL<br>14. OTHER MODERN METHOD (SPECIFY) _____________<br>15. OTHER TRADITIONAL METHOD (SPECIFY) __________
**600D.**	How old were you when you had sexual intercourse for the very first time?	[ ][ ] AGE IN YEARS<br>88. DOES NOT REMEMBER<br>99. REFUSED
**601F.**	**INTERVIEWER: CHECK AGE OF RESPONDENT (Q102)**	**1. 15–24 YEARS**<br>**2. 25–49 YEARS → SECTION VII**
**603.**	What was your relationship with the person with whom you first had sexual intercourse?	1. HUSBAND/LIVE-IN PARTNER<br>2. FIANCÉ<br>3. BOYFRIEND<br>4. FRIEND<br>5. ACQUAINTANCE<br>6. RELATIVE<br>7. JUST MET<br>8. RAPE/INCEST → **Q611**<br>20. OTHER (SPECIFY) __________<br>88. DOES NOT REMEMBER<br>99. REFUSED
**604.**	How old was the person you first had sexual intercourse with?<br>**PROBE: "Was this person older than you, younger than you, or about the same age as you?" AND OBTAIN AN ESTIMATE.**	[ ][ ] AGE IN YEARS → **Q605**<br>88. DOES NOT REMEMBER<br>99. REFUSED<br>**IF "88" OR "99" ASK Q604A; OTHERWISE GO TO Q605.**
**604A.**	How much older or younger was the person with whom you had your first sexual experience? Was he…. **(READ 1–4)**<br>1. More than 10 years older?<br>2. 5-10 years older?<br>3. Less than 5 years older?<br>4. Younger?	1. MORE THAN 10 YEARS OLDER<br>2. 5-10 YEARS OLDER<br>3. LESS THAN 5 YEARS OLDER<br>4. YOUNGER<br>8. DOESN'T KNOW
**605.**	How would you describe the first time you had sexual intercourse? Would you say that you wanted to have sex, you did not want to have sex but it happened anyway, or were you forced to have sex?	1. WANTED TO HAVE SEX<br>2. DID NOT WANT BUT IT HAPPENED ANYWAY<br>3. FORCED TO HAVE SEX<br>8. DOES NOT REMEMBER

**606.**	The first time you had sexual intercourse did you or your partner use a contraceptive method?	1. YES<br>2. NO → **Q610**<br>8. DOES NOT REMEMBER → **Q610**<br>9. REFUSED → **Q610**
**607.**	What was this method?	1. FEMALE STERILIZATION, TUBAL LIGATION<br>2. MALE STERILIZATION, VASECTOMY<br>3. IUD<br>4. INJECTABLES<br>5. IMPLANT<br>6. PILL<br>7. MALE CONDOM<br>8. FEMALE CONDOM<br>9. DIAPHRAGM<br>10. FOAM/JELLY<br>11. LAM<br>12. RHYTHM → **Q609**<br>13. WITHDRAWAL → **Q609**<br>14. OTHER MODERN METHOD (SPECIFY) ________________<br>15. OTHER TRADITIONAL METHOD (SPECIFY) ____________
**608.**	Where did you get this method?	1. GOVERNMENT HOSPITAL (SPECIFY) __________________<br>2. GOVERNMENT HEALTH CENTER (SPECIFY) ____________<br>3. GOVT. DISPENSARY (SPECIFY) _______________________<br>4. RELIGIOUS/PRIVATE HEALTH FACILITY<br>5. PHARMACY<br>6. NGO<br>7. CBD/ VILLAGE HEALTH WORKER<br>8. OTHER (SPECIFY) __________________________________
**609.**	Whose decision was it to use the method? Was it **…**<br>1. Your decision?<br>2. Your partner's decision?<br>3. Decision made together?	1. YOUR DECISION<br>2. YOUR PARTNER'S DECISION<br>3. DECISION MADE TOGETHER<br>8. DOES NOT REMEMBER<br>(1, 2, 3, 8) → **Q611**
**610.**	What was the main reason for not using a contraceptive method at the time of first sexual intercourse?	1. SEX WAS NOT EXPECTED<br>2. DID NOT KNOW ABOUT CONTRACEPTION<br>3. DID NOT KNOW WHERE TO GET CONTRACEPTIVES<br>4. DID NOT THINK IT WAS NECESSARY<br>5. PARTNER OBJECTED<br>6. RESPONDENT OBJECTED<br>7. SHE WANTED TO BECOME PREGNANT<br>8. NEGLIGENCE<br>9. COULD NOT GET A METHOD/ NOT AVAILABLE<br>10. TOO EXPENSIVE<br>11. FORCED SEX<br>20. OTHER (SPECIFY)______________________<br>88. DOES NOT KNOW / DOES NOT REMEMBER
**611.**	What is your preferred source of information about family life or sex education topics?<br><br>**CIRCLE ONLY ONE.**	1. PARENT(S) / GUARDIAN(S)<br>2. SISTER(S) OR BROTHER(S)<br>3. OTHER RELATIVE<br>4. TEACHERS<br>5. PEERS / FRIENDS<br>6. CLINIC STAFF<br>7. COMMUNITY HEALTH WORKER<br>8. RELIGIOUS LEADER<br>9. RADIO PROGRAM<br>10. YOUTH TV PROGRAM<br>11. OTHER TELEVISION PROGRAMS OR MESSAGES<br>12. PRINTED MATERIALS (BOOKS, BROCHURES, POSTERS)<br>13. WAZAZI NIPENDENI (MOBILE PHONE PROGRAM)<br>14. INTERNET<br>20. OTHER (SPECIFY):_________________________<br>88. DOES NOT KNOW
**611F.**	**INTERVIEWER: CHECK Q314 (P. 4) AND RECORD IF RESPONDENT HAD EVER HAD A LIVE BIRTH**	**1. YES**<br>**2. NO → GO TO SECTION VII**
**612.**	When pregnant with your first child, were you still in school?	1. YES<br>2. NO → **GO TO SECTION VII**
**613.**	What level and grade of schooling were you in?	1. PRIMARY _____ _____<br>2. SECONDARY _____ _____<br>77. TERTIARY<br>88. OTHER
**613A.**	Did you have to leave school because of the pregnancy?	1. YES<br>2. NO → **GO TO SECTION VII**
**613B.**	After the first child was born, did you return to school?	1. YES<br>2. NO

SECTION VII – ATTITUDES TOWARDS CONTRACEPTION AND CHILDBEARING

**700.**	Now I would like to talk about something else. Have you ever tried to obtain a contraceptive method and been refused?	1. YES<br>2. NO → **Q703B**<br>8. DOES NOT REMEMBER → **Q703B**
**701.**	How many times have you been refused?	[ ][ ] TIMES<br><br>88. DOES NOT REMEMBER
**702.**	In what place were you refused a contraceptive method?<br>**PROBE: Any place else?**<br><br>**CIRCLE '1' FOR ALL RESPONSES MENTIONED CIRCLE '2' IF NOT MENTIONED**	MEN-TIONED / NOT MEN-TIONED<br>A. GOVERNMENT HOSPITAL (SPECIFY) _________ 1 2<br>B. GOV'T HEALTH CENTER (SPECIFY)__________ 1 2<br>C. GOV'T DISPENSARY (SPECIFY)______________ 1 2<br>D. RELIGIOUS / PRIVATE HEALTH FACILITY 1 2<br>E. PHARMACY 1 2<br>F. NGO 1 2<br>G. CBD/ VILLAGE HEALTH WORKER 1 2<br>H. OTHER (SPECIFY) ___________________ 1 2
**703.**	What was the gender of the <u>last person</u> who refused to provide a contraceptive method?	1. MALE<br>2. FEMALE<br>8. DOES NOT REMEMBER
**703B.**	If you wanted to get information on family planning, who would you like to talk to most?	1. PARENT(S) / GUARDIAN(S)<br>2. SISTER(S) OR BROTHER(S)<br>3. OTHER RELATIVE<br>4. TEACHERS<br>5. PEERS / FRIENDS<br>6. CLINIC STAFF<br>7. COMMUNITY HEALTH WORKER<br>8. RELIGIOUS LEADER<br>9. PHARMACIST<br>10. RADIO PROGRAM OR MESSAGES<br>11. TELEVISION PROGRAMS OR MESSAGES<br>12. PRINTED MATERIALS (BOOKS, BROCHURES, POSTERS)<br>13. WAZAZI NIPENDENI (MOBILE PHONE PROGRAM)<br>14. INTERNET<br>20. OTHER (SPECIFY) _______________________<br>88. DOES NOT KNOW
**703C.**	Is it acceptable to you for information on family planning to be provided: **(READ A-F)**<br>A. On the radio?<br>B. On the television?<br>C. In a newspaper or magazine?<br>D. In school?<br>E. In religious settings (church/mosque/etc?)<br>F. Through a mobile phone?	YES / NO<br>A. RADIO 1 2<br>B. TELEVISION 1 2<br>C. NEWSPAPER/MAG 1 2<br>D. SCHOOL 1 2<br>E. CHURCH/MOSQUE 1 2<br>F. MOBILE PHONE 1 2
**703D.**	In the <u>last 6 months</u> have you heard about family planning: **(READ A-L)**<br>A. On the radio?<br>B. On the television?<br>C. In a newspaper or magazine?<br>D. From a poster?<br>E. From billboards?<br>F. At community events?<br>G. From live drama?<br>H. From a doctor or nurse?<br>I. From a community health worker?<br>J. Through a mobile phone?	YES / NO<br>A. RADIO 1 2<br>B. TELEVISION 1 2<br>C. NEWSPAPER/MAG 1 2<br>D. POSTER 1 2<br>E. BILLBOARDS 1 2<br>F. COMM. EVENTS 1 2<br>G. LIVE DRAMA 1 2<br>H. DOCTOR/NURSE 1 2<br>I. COMM. HW 1 2<br>J. MOBILE PHONE 1 2
**703E.**	Do you personally own a cell phone?	1. YES<br>2. NO
**704A.**	Have you heard of the Wazazi Nipendeni safe motherhood text message services that sends free health messages by mobile phone to pregnant women and new mothers?	1. YES<br>2. NO → **Q706**<br>8. DON'T KNOW → **Q706**

No.	Question	Coding categories	YES	NO
**704A1.**	How did you learn about the Wazazi Nipendeni service? **(READ A-F)**		**YES**	**NO**
	A. On the radio?	A. RADIO	1	2
	B. On television?	B. TELEVISION	1	2
	C. In a newspaper or magazine?	C. NEWSPAPER/MAGAZINE	1	2
	D. On a poster?	D. POSTER	1	2
	E. On a billboard?	E. BILLBOARDS	1	2
	F. From a facility health worker?	F. FACILITY HEALTH WORKER	1	2
	G. From a community health volunteer?	G. COMM. HEALTH VOLUNTEER	1	2
	H. From a friend?	H. FRIEND	1	2
	I. From a family member?	I. FAMILY MEMBER	1	2
	J. Other (specify) ____________________________	J. OTHER (SPECIFY) _________________	1	2
**704B.**	Have you ever enrolled to receive the messages from the Wazazi Nipendeni services?	1. YES<br>2. NO → **Q704C**<br>8. DON'T KNOW → **Q704C**		
**704B1.**	Have you ever been enrolled in Wazazi Nipendeni by		**YES**	**NO**
	A. Facility health worker?	A. FACILITY HEALTH WORKER	1	2
	B. Community health volunteer?	B. COMM. HEALTH VOL.	1	2
	C. Family member?	C. FAMILY MEMBER	1	2
	D. Friend/neighbor?	D. FRIEND/NEIGHBOR	1	2
	E. Self?	E. SELF	1	2
	F. Other (specify) ___________________________?	F. OTHER (SPECIFY) _________________	1	2
**704B2.**	Have you received the following messages through Wazazi Nipendeni? **(READ A-J)**		**YES**	**NO**
	A. Antenatal care reminder text	A. ANTENATAL CARE	1	2
	B. Reminder about needing to test for HIV	B. TEST FOR HIV	1	2
	C. Reminder to go for malaria preventive treatment during pregnancy	C. MALARIA PREV. TREATMENT	1	2
	D. Sleeping under insecticide-treated mosquito nets during pregnancy	D. SLEEP WITH MOSQUITO NET	1	2
	E. Information on danger signs during pregnancy	E. DANGER SIGNS IN PREG.	1	2
	F. Nutrition during pregnancy	F. NURTITION DURING PREG.	1	2
	G. Reminder to deliver in a health facility	G. DELIVER IN FACILITY	1	2
	H. Reminder about breastfeeding within one hour of birth	H. BREASTFEED WITHIN 1 HOUR	1	2
	I. Reminder to exclusively breastfeed for 6 months	I. EXCLUSIVELY BF FOR 6 MOS	1	2
	J. Reminder that if a mother is HIV positive, her baby has to get tested 6 weeks after birth	J. HIV TEST FOR BABY	1	2
**704B3.**	How often do you have access to the phone through which you enrolled in Wazazi Nipendeni?	1. ALWAYS (7 DAYS PER WEEK)<br>2. 4-6 DAYS PER WEEK<br>3. 3 OR FEWER DAYS PER WEEK		
**704B4.**	How did you feel about the Wazazi Nipendei service? Were you say you were very satisfied, somewhat satisfied, or not satisfied?	1. VERY SATISFIED<br>2. SOMEWHAT SATISFIED<br>3. NOT SATISFIED<br>8. DON'T KNOW		
**704B5.**	Have you ever recommended this service to someone else?	1. YES } **Q706**<br>2. NO }		
**704C.**	Have you considered registering to receive Wazazi Nipendeni text messages?	1. YES<br>2. NO		
**706.**	Now I would like you to read this sentence to me.<br><br>**SHOW CARD TO RESPONDENT. HAVE HER READ ONE SENTENCE. USE A DIFFERENT SENTENCE FOR THE NEXT ELIGIBLE WOMAN IN THE HOUSEHOLD.**<br><br>**IF RESPONDENT CANNOT READ WHOLE SENTENCE, PROBE:**<br><br>Can you read any part of the sentence to me?<br><br>**CARD READS:**<br>PARENTS LOVE THEIR CHILDREN.<br>FARMING IS HARD WORK.<br>THE CHILD IS READING A BOOK.<br>CHILDREN WORK HARD AT SCHOOL.	1. CANNOT READ AT ALL<br><br>2. ABLE TO READ ONLY PARTS OF SENTENCE<br><br>3. ABLE TO READ WHOLE SENTENCE<br><br>4. NO CARD WITH REQUIRED LANGUAGE<br><br>5. BLIND/VISUALLY IMPAIRED		

**707.** In the last 6 months, have you heard or seen a message on radio or television giving information about … **(READ A-E)**	**RADIO ONLY**	**TV ONLY**	**YES BOTH**	**NO, NEITHER**	**DOES NOT REMEMBER**	
A. Delivering your baby in a health facility?	1	2	3	4	8	
B. Developing a Birth Plan in pregnancy?	1	2	3	4	8	
C. HIV/AIDS?	1	2	3	4	8	
D. Other STDs?	1	2	3	4	8	
E. Domestic violence	1	2	3	4	8	
**708.** In your opinion, how would you describe the following contraceptive methods with regard to their effectiveness in preventing pregnancy? If the method is used correctly, would you say **(READ METHOD A-D)** is very effective, effective, somewhat effective, or not effective?	**VERY EFFECTIVE**	**EFFECTIVE**	**SOMEWHAT EFFECTIVE**	**NOT EFFECTIVE**	**DOES NOT KNOW**	
A. Pill	1	2	3	4	8	
B. Injection	1	2	3	4	8	
C. Condom	1	2	3	4	8	
D. Implants / IUDs	1	2	3	4	8	
**709.** Couples may use condoms to avoid getting sexually transmitted diseases. How effective do you think a properly used condom is for this purpose?	**VERY EFFECTIVE**	**EFFECTIVE**	**SOMEWHAT EFFECTIVE**	**NOT EFFECTIVE**	**DOES NOT KNOW**	
	1	2	3	4	8	
**710.** In your opinion, how would you describe the following contraceptive methods with regard to their risk of developing health problems? If the method is used correctly, would you say (**READ METHOD A-D**) is very safe, safe, somewhat safe, or not safe?	**VERY SAFE**	**SAFE**	**SOMEWHAT SAFE**	**NOT SAFE**	**DOES NOT KNOW**	
A. Pill	1	2	3	4	8	
B. Injection	1	2	3	4	8	
C. Condom	1	2	3	4	8	
D. Implants / IUDs	1	2	3	4	8	
**711.** To what extent do you agree or disagree with the following statements? Do you strongly disagree, disagree, neither disagree nor agree, agree, or strongly agree? (**READ STATEMENTS A-E.)**	**STRONGLY DISAGREE**	**DISAGREE**	**NEITHER DISAGREE NOR AGREE**	**AGREE**	**STRONGLY AGREE**	**DOES NOT KNOW**
A. Family planning is important for the welfare of a family.	1	2	3	4	5	8
B. Use of birth control pills or injections will reduce my ability to get pregnant in the future.	1	2	3	4	5	8
C. The decision to use contraception or not is one that should be made equally by the man and woman.	1	2	3	4	5	8
D. Families should only have children if they can afford to take care of their needs, including food, health care, clothing, and schooling.	1	2	3	4	5	8
E. Families should have many children because some of them will die.	1	2	3	4	5	8

**712.** How old do you think it is best for a child to be before another child is born?	[ ][ ] MONTHS<br>66. FATE, UP TO GOD<br>77. MORE THAN 5 YEARS<br>88. NOT SURE
**713.** How old do you think a child should be before the mother stops breastfeeding him / her?	[ ][ ] MONTHS<br>77. NO AGE LIMIT, AS LONG AS POSSIBLE<br>88. NOT SURE

714. **THANK THE RESPONDENT FOR PARTICIPATING IN THE SURVEY AND RECORD THE TIME THE INTERVIEW HAD ENDED:**

[ ][ ] **HOUR** [ ][ ] **MIN**

INTERVIEWER'S OBSERVATIONS

TO BE FILLED IN AFTER COMPLETING INTERVIEW

**715. WAS THE INTERVIEW CONDUCTED IN COMPLETE PRIVACY?**	**1. YES**<br>**2. NO**
**716. DID THE WOMAN CARE FOR A BABY OR SMALL CHILD DURING THE INTERVIEW?**	**1. YES**<br>**2. NO**
**717. DID ANYONE INTERRUPT THE INTERVIEW?**	**1. YES**<br>**2. NO → Q720**
**718. WHO INTERRUPTED THE INTERVIEW?**<br><br>**CIRCLE ALL THAT APPLY**	**A. HUSBAND/WIFE**<br>**B. SON/DAUGHTER**<br>**C. SON-IN-LAW/DAUGHTER-IN-LAW**<br>**D. GRANDCHILD**<br>**E. PARENT/PARENT/IN-LAW**<br>**F. BROTHER/SISTER**<br>**G. OTHER RELATIVE**<br>**H. NEIGHBOR, NOT RELATED**<br>**I. FRIEND, NOT RELATED**<br>**J. OTHER (SPECIFY) ___________________**
**719. HOW MANY TIMES WAS THE INTERVIEW INTERRUPTED?**	**____ ____ TIMES**
**720. DID THE RESPONDENT DISPLAY ANY OF THE FOLLOWING BEHAVIOR AT ANY POINT DURING THE INTERVIEW?**<br><br>**CIRCLE YES/NO FOR EACH**	**YES / NO**<br>**1. SHY** 1 2<br>**2. EMBARRASSED** 1 2<br>**3. NERVOUS, SCARED** 1 2<br>**4. CONFUSED** 1 2<br>**5. OPEN, WILLING TO TALK** 1 2<br>**6. INTERESTED** 1 2<br>**7. EAGER, EXCITED** 1 2

COMMENTS ABOUT RESPONDENT:
____
____

COMMENTS ON SPECIFIC QUESTIONS:
____
____

OTHER COMMENTS:
____
____

SUPERVISOR'S OBSERVATIONS:
____
____

NAME OF SUPERVISOR: ____
